# Performance Analysis of the Direct Position Determination Method in the Presence of Array Model Errors

**DOI:** 10.3390/s17071550

**Published:** 2017-07-02

**Authors:** Ding Wang, Hongyi Yu, Zhidong Wu, Cheng Wang

**Affiliations:** 1 National Digital Switching System Engineering & Technological Research Center, Zhengzhou 450002, China; maxyucn@sohu.com (H.Y.); 3092004009wzd@163.com (Z.W.); wangcheng1988zz@gmail.com (C.W.); 2 Zhengzhou Information Science and Technology Institute, Zhengzhou 450002, China

**Keywords:** direct position determination (DPD), array signal processing, array model errors, mean square error (MSE), success probability (SP), Cramér-Rao bound (CRB)

## Abstract

The direct position determination approach was recently presented as a promising technique for the localization of a transmitting source with accuracy higher than that of the conventional two-step localization method. In this paper, the theoretical performance of a direct position determination estimator proposed by Weiss is examined for situations in which the array model errors are present. Our study starts from a matrix eigen-perturbation result, which expresses the perturbation of eigenvalues as a function of the disturbance added to the Hermitian matrix. The first-order asymptotic expression of the positioning errors is presented, from which an analytical expression for the mean square error of the direct localization is available. Additionally, explicit formulas for computing the probabilities of a successful localization are deduced. Finally, Cramér–Rao bound expressions for the position estimation are derived for two cases: (1) array model errors are absent and (2) array model errors are present. The obtained Cramér-Rao bounds provide insights into the effects of the array model errors on the localization accuracy. Simulation results support and corroborate the theoretical developments made in this paper.

## 1. Introduction

The techniques of emitter localization using direction of arrival (DOA) measurements [1,2,3,4,5] play an important role in many areas, including vehicle navigation, localization and tracking of acoustic sources, and location services of satellite communications. In such localization systems, a single moving observer or multiple stationary observers are used to determinate the positions of the emitters. Generally, each observer is equipped with an antenna array for measuring the DOAs of the transmitted sources, and the emitter can then be located at the intersection of a set of lines of bearing [6,7,8]. The location procedure described above is typically called the two-step method. In the first step, the signal parameters (e.g., DOA [1,2,3,4,5], time difference of arrival (TDOA) [9,10], time of arrival (TOA) [11,12], frequency difference of arrival (FDOA) [13,14], frequency of arrival (FOA) [15], and received signal strength (RSS) [16,17]) are separately measured at several stations. In the second step, a central station uses the measurements to estimate the position coordinates of the sources. The two-step procedure is also known as the decentralized approach [18]. Note that although the two-step procedure is widely applied to the modern localization system, it is difficult to yield the optimal position estimate from the point of view of statistical characteristics. The reason is that the signal parameters are obtained by ignoring the constraint that all measurements must correspond to a common source position. As a result, information loss between the two steps is unavoidable. Although it can be proved by the extended invariance principle (EXIP) [19] that the two-step method provides an asymptotically efficient estimate under certain conditions, these requirements cannot be easily met in practical scenarios.

To improve the accuracy of two-step location methods, a promising technique, called the direct position determination (DPD) approach, is proposed over the past few years. DPD is a centralized and single-step estimation technique in which the estimator uses exactly the same data as classical two-step methods but searches for the source location directly. Generally, the DPD method outperforms conventional two-step methods under low-signal-to-noise conditions and when there are relatively few samples; moreover, it does not encounter the association problem. More importantly, the DPD technique can be applied to many wireless positioning systems. Specifically, the DPD method for locating a narrowband radio emitter based on a Doppler shift is presented in [20,21], and DPD methods for locating a wideband source based on a time delay metric are proposed in [22,23,24]. Furthermore, DPD estimators using both the Doppler frequency and time delay are developed in [25,26,27,28]. Note that in the DPD methods mentioned above, multiple platforms each equipped with a single-antenna receiver are used for position determination, and as a result, the DOA information of the impinging signals cannot be exploited. In [29], a DPD method based on multiple static stations each equipped with an antenna array is first proposed. In this single-step location method, the array response is modeled as a function of the source position, and only a two-dimensional search is required although there are many stray parameters in the array signal model. Following the work of [29], other DPD estimators for special localization scenarios are developed in the literature. In particular, DPD methods for multiple radio emitters are presented in [30,31], and some high-resolution DPD methods are given in [32,33]. DPD estimators for the cases of known waveforms and multipath environments are developed in [34] and [35,36], respectively. In addition, DPD methods tailored to special signals (e.g., orthogonal frequency division multiplexing signals, cyclostationary signals, and intermittent emissions) are proposed in [37,38,39]. It is noteworthy that all experiment results in [20,21,22,23,24,25,26,27,28,29,30,31,32,33,34,35,36,37,38,39] demonstrate that the single-step approach outperforms the two-step method for a low signal-to-noise ratio (SNR) and small number of samples. Meanwhile, although this kind of localization method may require more computations and communication bandwidth, novel information technology [40,41,42] can be used to overcome these difficulties. For example, the cloud computing and cloud storage technology [40,41] can be used to reduce the computation loads, and the compressive sensing technology [42] is helpful for reducing the communication bandwidth.

In the field of array signal processing, super-resolution DOA estimation methods are known to be sensitive to uncertainties in the array manifold. In recent decades, much attention has been paid to the analysis of the sensitivity of classical DOA estimation algorithms to array model errors. In [43,44,45,46,47,48,49,50,51], the statistic performance of the multiple signal classification algorithm and its extensions in the presence of array model errors is studied. An analysis of the estimation of signal parameters via rotational invariance techniques under random sensor uncertainties is performed in [52], and a sensitivity analysis of the weighted subspace fitting algorithm under the combined effects of array model errors and finite samples is presented in [53]. The statistical performance of the maximum likelihood algorithm is also investigated in [54,55] assuming that array calibration errors exist. Additionally, efficient parameter estimation algorithms are proposed with an uncalibrated array [56,57] or partly calibrated array [58,59,60].

Array model errors are typically caused by gain/phase uncertainties, mutual coupling, and sensor position perturbations. Note that all DPD methods presented in [29,30,31,32,33,35,36,37,38,39] also rely on the accurate knowledge of the array manifold and, therefore, it seems reasonable to expect that their localization accuracy is also severely degraded by array uncertainties. Although the estimation performance of the DPD method in the presence of array model errors is rigorously analyzed in [34,61,62], these theoretical studies are simply performed for the case where signal waveforms are known. However, this is rarely realistic for non-cooperative communications. In this paper, the location performance of the DPD method in the presence of array model errors is examined when signal waveforms are not known in advance. Our theoretical analysis focuses on the DPD estimator in [29] because of its fundamental role in the field of direct localization. Because the objective function of this DPD estimator is formulated as the maximum eigenvalue of a Hermitian matrix, the theoretical development begins with a matrix eigen-perturbation result, which expresses the perturbation of eigenvalues as a function of the disturbance added to the Hermitian matrix. Subsequently, the first-order asymptotic expression of the localization errors is given, from which the analytical formula for the mean square error (MSE) of the DPD estimator is available. Furthermore, two exact formulations for the calculation of the probabilities of a successful localization are also deduced, which offers another statistical perspective on the study of the estimation performance. Finally, Cramér-Rao bound (CRB) expressions for the position estimation are derived for two cases: (a) array model errors do not exist and (b) array model errors are present and follow a Gaussian distribution. The obtained CRBs provide further insights into how array model errors affect the localization performance.

The remainder of this paper is organized as follows. Section 2 lists the notational conventions that will be used throughout the paper. In Section 3, the signal model for direct localization is formulated. Section 4 briefly describes the DPD method, which is first proposed in [29]. Section 5 discusses the statistical assumption and effects of the array model errors. In Section 6, the analytical formula for the MSE of the DPD method is derived in presence of array model errors. Section 7 provides two explicit formulas for the calculation of the probabilities of a successful localization. In Section 8, the CRB expressions for the position estimation are derived for two cases. Numerical simulations are presented in Section 9 to investigate the usefulness of the theoretical expressions for performance prediction. Conclusions are drawn in Section 10. The proofs of the main results are given in the Appendixes.

## 2. Notation and Nomenclature

The notational conventions that will be used throughout this paper are summarized in Table 1. The variables and parameters that are used in this paper will be defined when they first appear in the following.

## 3. Signal Models for Direct Position Determination

### 3.1. Time-Domain Signal Model

Consider an emitter and N base stations intercepting the transmitted signal. Each base station is equipped with an antenna array consisting of M elements. The transmitter’s position is denoted by an L×1 vector of coordinates p. In practice, L is equal to two or three, and cannot be larger than three. We consider the case where there is no multipath or non-line-of-sight (NLOS) phenomenon. The complex envelopes of the signal observed by the *n*th base station are then modeled by [29]

(1) $$x_{n}(t)=\beta_{n}a_{n}(p)s(t-\tau_{n}(p\;)-t_{0})+\varepsilon_{n}(t)\;(1\leq n\leq N)\;,$$

where

- $a_{n}(p)$ is the *n*th array response to the signal transmitted from position p,
- $s(t-\tau_{n}(p\;)-t_{0})$ is the unknown signal waveform transmitted at unknown time $t_{0}$,
- $\tau_{n}(p\;)$ is the signal propagation time from the emitter to the *n*th base station (i.e., distance divided by signal propagation speed),
- $\beta_{n}$ is an unknown complex scalar representing the channel attenuation between the transmitter and the *n*th base station,
- $\varepsilon_{n}(t)$ is temporally white, circularly symmetric complex Gaussian random noise with zero mean and covariance matrix $\sigma_{\varepsilon }^{2}I_{M}$.

Assuming the observation vector $x_{n}(t)$ is sampled with period T, the *k*th sampled data can be expressed as

(2) $$x_{n,k}=\beta_{n}a_{n}(p)s(kT-\tau_{n}(p\;)-t_{0})+\varepsilon_{n,k}\;(1\leq k\leq K)\;,$$

where K is the number of snapshots.

### 3.2. Frequency-Domain Signal Model

To determinate the emitter position directly from all observations, it is desirable to separate the propagation delay $\tau_{n}(p\;)$ and transmit time $t_{0}$ from the signal waveform. This is easily achieved using the frequency-domain representation of the problem. Taking the discrete Fourier transform (DFT) of (2) produces [29]

(3) $${\bar{x}}_{n,k}=\beta_{n}a_{n}(p){\bar{s}}_{k}\cdot \exp\{-j\omega_{k}(\tau_{n}(p)+t_{0})\}+{\bar{\varepsilon }}_{n,k}\;(1\leq n\leq N\;;\;1\leq k\leq K)\;,$$

where

- $\omega_{k}=2\pi (k-1)/(KT)$ is the *k*th known discrete frequency point,
- ${\bar{s}}_{k}$ is the *k*th Fourier coefficient of the unknown signal corresponding to frequency $\omega_{k}$,
- ${\bar{\varepsilon }}_{n,k}$ is the *k*th Fourier coefficient of the random noise corresponding to frequency $\omega_{k}$.

It must be emphasized that the unknown and deterministic parameter set in (3) consists of p , $t_{0}$, $\beta_{n}$ and ${\bar{s}}_{k}$. However, only the location vector p  is of interest for the DPD approach. In addition, because the DFT is an orthogonal linear transformation, the distribution of the random noise vector ${\bar{\varepsilon }}_{n,k}$ is the same as that of $\varepsilon_{n,k}$, with first- and second-order moments given by.

(4) $$\left\{ \begin{array}{l} E[{\bar{\varepsilon }}_{n,k}]=O_{M\times 1}\;,\\ E[{\bar{\varepsilon }}_{n,k}{\bar{\varepsilon }}_{n,k}^{T}]=O_{M\times M}\;,\;E[{\bar{\varepsilon }}_{n,k}{\bar{\varepsilon }}_{n,k}^{H}]=\sigma_{\varepsilon }^{2}I_{M}\;,\\ E[{\bar{\varepsilon }}_{n,k}{\bar{\varepsilon }}_{n,l}^{T}]=E[{\bar{\varepsilon }}_{n,k}{\bar{\varepsilon }}_{n,l}^{H}]=O_{M\times M}\;, \end{array}\right.\;(1\leq k\;,\;l\leq K\;;\;k\neq l)\;.$$

Note that the DPD technique studied below is derived from (3).

## 4. Direct Position Determination Method

This section introduces the DPD method presented in [29]. The optimization model for direct localization is established according to the least square criterion, which can be formulated as

(5) $$\underset{p,\{\beta_{n}\},\{{\bar{s}}_{k}\}}{\min}\sum_{n=1}^{N}\sum_{k=1}^{K}||{\bar{x}}_{n,k}-\beta_{n}a_{n}(p){\bar{s}}_{k}\cdot \exp\{-j\omega_{k}(\tau_{n}(p)+t_{0})\}|{|}_{2}^{2}=\underset{p,\{\beta_{n}\},\{{\bar{s}}_{k}\}}{\min}\sum_{n=1}^{N}||{\bar{x}}_{n}-({\bar{s}}_{n}^{\prime }⊗a_{n}(p))\beta_{n}|{|}_{2}^{2}\;,$$

where

(6) $$\left\{ \begin{array}{l} {\bar{x}}_{n}={\left[{\bar{x}}_{n,1}^{H}\;{\bar{x}}_{n,2}^{H}\;\cdots \;{\bar{x}}_{n,K}^{H}\right]}^{H}\;,\\ {\bar{s}}_{n}^{\prime }={\left[{\bar{s}}_{1}\cdot \exp\{-j\omega_{1}(\tau_{n}(p)+t_{0})\}\;{\bar{s}}_{2}\cdot \exp\{-j\omega_{2}(\tau_{n}(p)+t_{0})\}\;\cdots \;{\bar{s}}_{K}\cdot \exp\{-j\omega_{K}(\tau_{n}(p)+t_{0})\}\right]}^{T}\;. \end{array}\right.$$

Obviously, (5) is a multidimensional nonlinear minimization problem. A direct minimization involves a search over the parameter space and is computationally prohibitive. The technique of the separation of variables can be applied to simplify the optimization problem.

First, the channel attenuation scalar $\beta_{n}$ that minimizes (5) is given by

(7) $$\beta_{n}=\frac{1}{||a_{n}(p){\left|\right|}_{2}^{2}\cdot ||{\bar{s}}_{n}^{\prime }|{|}_{2}^{2}}\cdot {\left({\bar{s}}_{n}^{\prime }⊗a_{n}(p)\right)}^{H}{\bar{x}}_{n}\;.$$

It can be assumed, without loss of generality, that $||a_{n}(p)|{|}_{2}=||{\bar{s}}_{n}^{\prime }{\left|\right|}_{2}=1$. Then, substituting (7) into (5) and applying algebraic manipulations leads to the concentrated problem [29]

(8) $$\underset{p,\bar{s}}{\max}\;{\bar{s}}^{H}\left(\sum_{n=1}^{N}A_{n}^{H}(p){\bar{x}}_{n}{\bar{x}}_{n}^{H}A_{n}(p)\right)\bar{s}\;,$$

where

(9) $$\left\{ \begin{array}{l} A_{n}(p)=\mathrm{blkdiag}[a_{n,1}^{\prime }(p)\;a_{n,2}^{\prime }(p)\;\cdots \;a_{n,K}^{\prime }(p)]\\ \bar{s}={\left[{\bar{s}}_{1}\cdot \exp\{-j\omega_{1}t_{0}\}\;{\bar{s}}_{2}\cdot \exp\{-j\omega_{2}t_{0}\}\;\cdots \;{\bar{s}}_{K}\cdot \exp\{-j\omega_{K}t_{0}\}\right]}^{T} \end{array}\right.$$

with $a_{n,k}^{\prime }(p)=a_{n}(p)\cdot \exp\{-j\omega_{k}\tau_{n}(p)\}$. According to quadratic form theory, the cost function in (8) is maximized by selecting the vector $\bar{s}$ as the eigenvector corresponding to the largest eigenvalue of matrix $\sum_{n=1}^{N}A_{n}^{H}(p){\bar{x}}_{n}{\bar{x}}_{n}^{H}A_{n}(p)$. Therefore, (8) reduces to

(10) $$\underset{p}{\max}\;J(p)=\underset{p}{\max}\;\lambda_{\max}\{B(p)B^{H}(p)\}=\underset{p}{\max}\;\lambda_{\max}\{B^{H}(p)B(p)\}\;,$$

where

(11) $$B(p)=[A_{1}^{H}(p){\bar{x}}_{1}\;A_{2}^{H}(p){\bar{x}}_{2}\;\cdots \;A_{N}^{H}(p){\bar{x}}_{N}]=A^{H}(p)\bar{X}$$

with

(12) $$\left\{ \begin{array}{l} A(p)={\left[A_{1}^{H}(p)\;A_{2}^{H}(p)\;\cdots \;A_{N}^{H}(p)\right]}^{H}\;,\\ \bar{X}=\mathrm{blkdiag}[{\bar{x}}_{1}\;{\bar{x}}_{2}\;\cdots \;{\bar{x}}_{N}]\;. \end{array}\right.$$

It is important to stress that the second equality in (10) holds owing to the fact that given any matrix Z, the non-zero eigenvalues of $Z^{H}Z$ and $ZZ^{H}$ are identical [63]. Moreover, note that the dimensions of matrices $B(p)B^{H}(p)$ and $B^{H}(p)B(p)$ are respectively K×K and N×N. In practice, K is typically much greater than N and it is therefore more computationally efficient to perform the eigendecomposition on $B^{H}(p)B(p)$ instead of $B(p)B^{H}(p)$. Because the cost function in (10) is not a closed-form expression for p, the most straightforward method of solving (10) is to perform a grid search, as recommended in [29].

Note that when the location is estimated in multipath environments, the localization accuracy may obviously improve if the information contained in the non-line-of-sight signal components is exploited with the aid of appropriate channel modeling [35,36]. As a consequence, the signal model in (1) and (3) and the estimation criterion in (5) must be further adjusted to give a desired solution for the multipath model. Indeed, our performance analysis method also applies to the case of multipath propagation, but we only consider the single-path signal model in this paper owing to limited space.

## 5. Statistical Assumption and Effects of Array Model Errors

Assume that the actual array response, which differs from the nominal value, can be expressed as

(13) $${\hat{a}}_{n}(p)=a_{n}(p)+{\tilde{\varphi }}_{n}\;,$$

where ${\tilde{\varphi }}_{n}$ is the array model error. It must be emphasized that ${\tilde{\varphi }}_{n}$ is modeled as a stochastic variable rather than a deterministic variable throughout this paper. Moreover, there exist a variety of statistical assumptions that could be used to describe ${\tilde{\varphi }}_{n}$ in the literature. To make our results applicable to a more general situation, ${\left\{{\tilde{\varphi }}_{n}\right\}}_{1\leq n\leq N}$ is modeled as a set of independent complex Gaussian vectors with first- and second-order moments given by [43,44,45,46,47,48,49,50,51,52,53,54,55]

(14) $$\left\{ \begin{array}{l} E[{\tilde{\varphi }}_{n}]=O_{M\times 1}\;,\\ E[{\tilde{\varphi }}_{n}{\tilde{\varphi }}_{n}^{T}]=\Phi_{n}^{\left(1\right)}\;,\;E[{\tilde{\varphi }}_{n}{\tilde{\varphi }}_{n}^{H}]=\Phi_{n}^{\left(2\right)}\\ E[{\tilde{\varphi }}_{n}{\tilde{\varphi }}_{m}^{T}]=E[{\tilde{\varphi }}_{n}{\tilde{\varphi }}_{m}^{H}]=O_{M\times M}\;, \end{array}\right.\;,\;(1\leq n\;,\;m\leq N\;;\;n\neq m)\;.$$

Furthermore, array model error ${\tilde{\varphi }}_{n}$ is uncorrelated to sensor noise ${\left\{{\bar{\varepsilon }}_{n,k}\right\}}_{1\leq k\leq K}$ for each base station. It is noteworthy that (14) will be used to determine the MSE and the CRB of the DPD estimator investigated in this paper.

When array model errors exist, the frequency-domain signal model in (3) becomes

(15) $${\hat{\bar{x}}}_{n,k}={\bar{x}}_{n,k,0}+\beta_{n}{\tilde{\varphi }}_{n}{\bar{s}}_{k}\cdot \exp\{-j\omega_{k}(\tau_{n}(p)+t_{0})\}+{\bar{\varepsilon }}_{n,k}\;,$$

where ${\bar{x}}_{n,k,0}$ is the true value of ${\hat{\bar{x}}}_{n,k}$ in the absence of sensor noise and array model errors, and can be expressed as

(16) $${\bar{x}}_{n,k,0}=\beta_{n}a_{n}(p){\bar{s}}_{k}\cdot \exp\{-j\omega_{k}(\tau_{n}(p)+t_{0})\}\;.$$

Defining the vectors and matrices

(17) $$\left\{ \begin{array}{l} {\hat{\bar{x}}}_{n}={\left[{\hat{\bar{x}}}_{n,1}^{H}\;{\hat{\bar{x}}}_{n,2}^{H}\;\cdots \;{\hat{\bar{x}}}_{n,K}^{H}\right]}^{H}\;,\;{\bar{x}}_{n,0}={\left[{\bar{x}}_{n,1,0}^{H}\;{\bar{x}}_{n,2,0}^{H}\;\cdots \;{\bar{x}}_{n,K,0}^{H}\right]}^{H}\;,\;{\bar{\varepsilon }}_{n}={\left[{\bar{\varepsilon }}_{n,1}^{H}\;{\bar{\varepsilon }}_{n,2}^{H}\;\cdots \;{\bar{\varepsilon }}_{n,K}^{H}\right]}^{H}\;,\\ \hat{\bar{X}}=\mathrm{blkdiag}[{\hat{\bar{x}}}_{1}\;{\hat{\bar{x}}}_{2}\;\cdots \;{\hat{\bar{x}}}_{N}]\;,\;{\bar{X}}_{0}=\mathrm{blkdiag}[{\bar{x}}_{1,0}\;{\bar{x}}_{2,0}\;\cdots \;{\bar{x}}_{N,0}]\;,\;\bar{E}=\mathrm{blkdiag}[{\bar{\varepsilon }}_{1}\;{\bar{\varepsilon }}_{2}\;\cdots \;{\bar{\varepsilon }}_{N}]\;, \end{array}\right.$$

it is easily verified from (15) and (17) that

(18) $${\hat{\bar{x}}}_{n}={\bar{x}}_{n,0}+{\bar{\varepsilon }}_{n}+(r_{n}⊗I_{M}){\tilde{\varphi }}_{n}\;(1\leq n\leq N)\;,$$

where

(19) $$r_{n}=\beta_{n}\cdot {\left[{\bar{s}}_{1}\cdot \exp\{-j\omega_{1}(\tau_{n}(p)+t_{0})\}\;{\bar{s}}_{2}\cdot \exp\{-j\omega_{2}(\tau_{n}(p)+t_{0})\}\;\cdots \;{\bar{s}}_{K}\cdot \exp\{-j\omega_{K}(\tau_{n}(p)+t_{0})\}\right]}^{T}\;.$$

From (17) and (18) we get

(20) $$\begin{array}{l} \mathrm{blkdiag}[{\hat{\bar{x}}}_{1}\;{\hat{\bar{x}}}_{2}\;\cdots \;{\hat{\bar{x}}}_{N}]=\mathrm{blkdiag}[{\bar{x}}_{1,0}\;{\bar{x}}_{2,0}\;\cdots \;{\bar{x}}_{N,0}]+\mathrm{blkdiag}[{\bar{\varepsilon }}_{1}\;{\bar{\varepsilon }}_{2}\;\cdots \;{\bar{\varepsilon }}_{N}]\\ +\mathrm{blkdiag}[(r_{1}⊗I_{M}){\tilde{\varphi }}_{1}\;(r_{2}⊗I_{M}){\tilde{\varphi }}_{2}\;\cdots \;(r_{N}⊗I_{M}){\tilde{\varphi }}_{N}]\Leftrightarrow \hat{\bar{X}}={\bar{X}}_{0}+\bar{E}+\tilde{\Psi }\;, \end{array}$$

where

(21) $$\tilde{\Psi }=\mathrm{blkdiag}[(r_{1}⊗I_{M}){\tilde{\varphi }}_{1}\;(r_{2}⊗I_{M}){\tilde{\varphi }}_{2}\;\cdots \;(r_{N}⊗I_{M}){\tilde{\varphi }}_{N}]\;.$$

In the presence of array model errors, the emitter position is actually determined by

(22) $$\underset{p}{\max}\;\hat{J}(p)=\underset{p}{\max}\;\lambda_{\max}\{{\hat{B}}^{H}(p)\hat{B}(p)\}\;,$$

where $\hat{B}(p)=A^{H}(p)\hat{\bar{X}}$. We assume the optimal solution to (22) is $\hat{p}$ and its estimate error is $\tilde{p}=\hat{p}-p$. It is evident that the estimate error $\tilde{p}$ depends on both sensor noise and array model errors. In subsequent sections, the statistical performance of $\tilde{p}$ is derived under the combined effects of the two sources of error.

For convenience in later formulae, we proceed by defining two error vectors

(23) $${\bar{\varepsilon }}_{c}=\left[\begin{array}{c} \bar{\varepsilon }\\ {\bar{\varepsilon }}^{*} \end{array}\right]\;,\;{\tilde{\varphi }}_{c}=\left[\begin{array}{c} \tilde{\varphi }\\ {\tilde{\varphi }}^{*} \end{array}\right]\;,$$

where

(24) $$\bar{\varepsilon }={\left[{\bar{\varepsilon }}_{1}^{H}\;{\bar{\varepsilon }}_{2}^{H}\;\cdots \;{\bar{\varepsilon }}_{N}^{H}\right]}^{H}=\bar{E}1_{N\times 1}\;,\;\tilde{\varphi }={\left[{\tilde{\varphi }}_{1}^{H}\;{\tilde{\varphi }}_{2}^{H}\;\cdots \;{\tilde{\varphi }}_{N}^{H}\right]}^{H}\;.$$

Obviously, ${\bar{\varepsilon }}_{c}$ and ${\tilde{\varphi }}_{c}$ are related to sensor noise and array model errors, respectively. Further, we define two permutation matrices

(25) $$\Pi_{\bar{\varepsilon }}=\left[\begin{array}{cc} O_{MNK\times MNK} & I_{MNK}\\ I_{MNK} & O_{MNK\times MNK} \end{array}\right]\;,\;\Pi_{\tilde{\varphi }}=\left[\begin{array}{cc} O_{MN\times MN} & I_{MN}\\ I_{MN} & O_{MN\times MN} \end{array}\right]\;,$$

It can then be easily checked from (23) and (25) that ${\bar{\varepsilon }}_{c}=\Pi_{\bar{\varepsilon }}{\bar{\varepsilon }}_{c}^{*}$ and ${\tilde{\varphi }}_{c}=\Pi_{\tilde{\varphi }}{\tilde{\varphi }}_{c}^{*}$. In addition, it is straightforward to deduce from (17), (21), and (24) that $\bar{E}=O(||\bar{\varepsilon }|{|}_{2})$ and $\tilde{\Psi }=O(||\tilde{\varphi }|{|}_{2})$.

## 6. MSE of Direct Position Determination Method in Presence of Array Model Errors

In this section, the MSE for the DPD method stated above is addressed in the presence of uncertainties in the model of the array manifold.

### 6.1. Perturbation Analysis on the Eigenvalues of Positive Semidefinite Matrix

Because the cost function in (22) is expressed as the maximal eigenvalue of some positive semidefinite matrix, an eigenvalue perturbation result is formally stated in a proposition as follows.

**Proposition** **1.** *Let* $Z\in C^{N\times N}$ *be a positive semidefinite matrix with eigenvalues* $\lambda_{1}\leq \lambda_{2}\leq \cdots \leq \lambda_{N}$*, associated with unit eigenvectors* $u_{1}\;,\;u_{2}\;,\;\cdots \;,\;u_{N}$*, respectively. Moreover,* $\lambda_{n}$ *differs from the other eigenvalues. Assume* Z *is corrupted by a Hermitian error matrix* $\tilde{Z}\in C^{N\times N}$*, and the corresponding perturbed matrix is denoted* $\hat{Z}$*; i.e.,* $\hat{Z}=Z+\tilde{Z}\in C^{N\times N}$*. If the eigenvalues of matrix* $\hat{Z}$ *are denoted* ${\hat{\lambda }}_{1}\leq {\hat{\lambda }}_{2}\leq \cdots \leq {\hat{\lambda }}_{N}$*, then the relationship between* ${\hat{\lambda }}_{n}$ *and* $\lambda_{n}$ *can be described by*

(26) $${\hat{\lambda }}_{n}=\lambda_{n}+u_{n}^{H}\tilde{Z}u_{n}+u_{n}^{H}\tilde{Z}U_{n}\tilde{Z}u_{n}+o(||\tilde{Z}|{|}_{2}^{2})\;,$$

where

(27) $$U_{n}=\sum_{\begin{array}{c} i=1\\ i\neq n \end{array}}^{N}\frac{u_{i}u_{i}^{H}}{\lambda_{n}-\lambda_{i}}\;.$$

The proof of Proposition 1 can be found in [21]. Note that Proposition 1 plays a fundamental role in our subsequent analysis.

### 6.2. Second-Order Perturbation Analysis on the Cost Function

Generally, first-order analysis is applied to predict the statistical performance of an estimator. The reason is that this analysis method gives the linear relationship between the estimation errors and measurement noise as well as model errors. As a result, the theoretical MSE of the estimator can be obtained according to statistical assumptions of the two sources of error. Moreover, first-order analysis is valid in most cases, provided that the error levels are not too high. In this paper, we employ this approach to derive the performance of the DPD estimator described above. For this purpose, first-order perturbation analysis is performed on the first derivative of the objective function in (22), or alternatively, second-order perturbation analysis is performed on the cost function in (22). Herein, because the analytical expression for the derivative of the cost function is rather complex, we prefer the second approach.

First, performing second-order perturbation analysis on matrix $\hat{B}(\hat{p})=A^{H}(\hat{p})\hat{\bar{X}}$ leads to

(28) $$\hat{B}(\hat{p})=A^{H}(\hat{p})\hat{\bar{X}}=B_{0}+{\tilde{B}}^{\left(1\right)}+{\tilde{B}}^{\left(2\right)}+o(||\tilde{\xi }|{|}_{2}^{2})\;,$$

where $\tilde{\xi }={\left[{\tilde{p}}^{T}\;{\bar{\varepsilon }}^{T}\;{\tilde{\varphi }}^{T}\right]}^{T}$ consists of all error vectors, and

(29) $$\left\{ \begin{array}{l} B_{0}=A^{H}(p){\bar{X}}_{0}\\ {\tilde{B}}^{\left(1\right)}=A^{H}(p)\bar{E}+A^{H}(p)\tilde{\Psi }+\sum_{l=1}^{L}<\tilde{p}{>}_{l}\cdot {\dot{A}}_{l}^{H}(p){\bar{X}}_{0}=O(||\xi |{|}_{2})\\ {\tilde{B}}^{\left(2\right)}=\sum_{l=1}^{L}<\tilde{p}{>}_{l}\cdot {\dot{A}}_{l}^{H}(p)\bar{E}+\sum_{l=1}^{L}<\tilde{p}{>}_{l}\cdot {\dot{A}}_{l}^{H}(p)\tilde{\Psi }+\frac{1}{2}\cdot \sum_{l_{1}=1}^{L}\sum_{l_{2}=1}^{L}<\tilde{p}{>}_{l_{1}}\cdot <\tilde{p}{>}_{l_{2}}\cdot {\ddot{A}}_{l_{1}l_{2}}^{H}(p){\bar{X}}_{0}=O(||\xi |{|}_{2}^{2}) \end{array}\right.$$

with

(30) $${\dot{A}}_{l}(p)=\frac{\partial A(p)}{\partial <p{>}_{l}}\;,\;{\ddot{A}}_{l_{1}l_{2}}(p)=\frac{\partial^{2}A(p)}{\partial <p{>}_{l_{1}}\partial <p{>}_{l_{2}}}\;.$$

The explicit expressions for ${\dot{A}}_{l}(p)$ and ${\ddot{A}}_{l_{1}l_{2}}(p)$ are given in Appendix A. It is seen from (28) and (29) that ${\tilde{B}}^{\left(1\right)}$ and ${\tilde{B}}^{\left(2\right)}$ collect all first- and second-order perturbation terms, respectively. It is deduced from (28) that

(31) $${\hat{B}}^{H}(\hat{p})\hat{B}(\hat{p})=C_{0}+{\tilde{C}}^{\left(1\right)}+{\tilde{C}}^{\left(2\right)}+o(||\tilde{\xi }|{|}_{2}^{2})\;,$$

where

(32) $$\left\{ \begin{array}{l} C_{0}=B_{0}^{H}B_{0}\;,\;{\tilde{C}}^{\left(1\right)}=B_{0}^{H}{\tilde{B}}^{\left(1\right)}+{\tilde{B}}^{(1)H}B_{0}=O(||\xi |{|}_{2})\;,\\ {\tilde{C}}^{\left(2\right)}={\tilde{B}}^{(1)H}{\tilde{B}}^{\left(1\right)}+B_{0}^{H}{\tilde{B}}^{\left(2\right)}+{\tilde{B}}^{(2)H}B_{0}=O(||\xi |{|}_{2}^{2})\;. \end{array}\right.$$

From (31) and (32) we observe that ${\tilde{C}}^{\left(1\right)}$ and ${\tilde{C}}^{\left(2\right)}$ consist of all first- and second-order perturbation terms, respectively.

Let $\lambda_{1}\leq \lambda_{2}\leq \cdots \leq \lambda_{N}$ and $u_{1},u_{2},\cdots ,u_{N}$ be the eigenvalues and relevant unit eigenvectors of matrix $C_{0}$, respectively. Additionally, it is not unreasonable to assume that the source location parameters are identifiable, which means that $C_{0}$ has unique maximal eigenvalue $\lambda_{N}$. Meanwhile, it is noteworthy that the eigenvalue perturbation theory is extensively applied to the performance analysis in array signal processing for DOA estimation. To our best knowledge, there is no relevant mathematical tool that can be used to prove that the eigenvalues of $C_{0}$ are distinct. However, a large number of numerical investigations demonstrate that the possibility of the case of equal eigenvalues is small enough that we can ignore it. As a result, we define the matrix

(33) $$U_{N}=\sum_{n=1}^{N-1}\frac{u_{n}u_{n}^{H}}{\lambda_{N}-\lambda_{n}}\;.$$

By combining Proposition 1 and (31), the cost-function value at point $\hat{p}$ is given by

(34) $$\begin{array}{ll} \hat{J}(\hat{p}) & =\lambda_{\max}\{{\hat{B}}^{H}(\hat{p})\hat{B}(\hat{p})\}=\lambda_{\max}\{C_{0}\}+u_{N}^{H}({\tilde{C}}^{\left(1\right)}+{\tilde{C}}^{\left(2\right)})u_{N}+u_{N}^{H}{\tilde{C}}^{\left(1\right)}U_{N}{\tilde{C}}^{\left(1\right)}u_{N}+o({\left||\tilde{\xi }|\right|}_{2}^{2})\\ & =\lambda_{N}+{\tilde{\lambda }}_{N}^{\left(1\right)}+{\tilde{\lambda }}_{N}^{\left(2\right)}+o({\left||\tilde{\xi }|\right|}_{2}^{2})\;, \end{array}$$

where

(35) $$\left\{ \begin{array}{l} {\tilde{\lambda }}_{N}^{\left(1\right)}=u_{N}^{H}B_{0}^{H}{\tilde{B}}^{\left(1\right)}u_{N}+{\left(u_{N}^{H}B_{0}^{H}{\tilde{B}}^{\left(1\right)}u_{N}\right)}^{H}=O(||\tilde{\xi }|{|}_{2})\;,\\ {\tilde{\lambda }}_{N}^{\left(2\right)}=u_{N}^{H}B_{0}^{H}{\tilde{B}}^{\left(1\right)}U_{N}B_{0}^{H}{\tilde{B}}^{\left(1\right)}u_{N}+{\left(u_{N}^{H}B_{0}^{H}{\tilde{B}}^{\left(1\right)}U_{N}B_{0}^{H}{\tilde{B}}^{\left(1\right)}u_{N}\right)}^{H}+u_{N}^{H}{\tilde{B}}^{(1)H}(I_{K}+B_{0}U_{N}B_{0}^{H}){\tilde{B}}^{\left(1\right)}u_{N}\\ \;+u_{N}^{H}B_{0}^{H}{\tilde{B}}^{\left(1\right)}U_{N}{\tilde{B}}^{(1)H}B_{0}u_{N}+u_{N}^{H}B_{0}^{H}{\tilde{B}}^{\left(2\right)}u_{N}+{\left(u_{N}^{H}B_{0}^{H}{\tilde{B}}^{\left(2\right)}u_{N}\right)}^{H}=O(||\tilde{\xi }|{|}_{2}^{2})\;. \end{array}\right.$$

It is seen from (35) that ${\tilde{\lambda }}_{N}^{\left(1\right)}$ and ${\tilde{\lambda }}_{N}^{\left(2\right)}$ group together all the first- and second-order error terms, respectively. The proof of (34) and (35) can be found in Appendix B. In the following, we express ${\tilde{\lambda }}_{N}^{\left(1\right)}$ and ${\tilde{\lambda }}_{N}^{\left(2\right)}$ as functions of ${\bar{\varepsilon }}_{c}$, ${\tilde{\varphi }}_{c}$, and $\tilde{p}$.

First, inserting the second equality in (29) into the first equality in (35) produces

(36) $${\tilde{\lambda }}_{N}^{\left(1\right)}=h_{1}^{H}(p){\bar{\varepsilon }}_{c}+h_{2}^{H}(p){\tilde{\varphi }}_{c}+h_{3}^{T}(p)\tilde{p}\;,$$

where

(37) {h1(p)=f1[B0uN,uN]+Πε¯(f1[B0uN,uN])∗ ,h2(p)=f2[B0uN,uN]+Πφ˜(f2[B0uN,uN])∗ ,h3(p)=2⋅Re{f3[B0uN,uN]} ,

in which ${\left\{f_{k}[\cdot \;,\;\cdot ]\right\}}_{1\leq k\leq 3}$ can be regarded as a set of vector functions, whose functional forms are given by

(38) $$\left\{ \begin{array}{l} f_{1}[z_{1},z_{2}]=\left[\begin{array}{c} \mathrm{diag}[z_{2}^{*}⊗1_{MK\times 1}]\cdot A(p)z_{1}\\ O_{MNK\times 1} \end{array}\right]\;,\\ f_{2}[z_{1},z_{2}]=\left[\begin{array}{c} \mathrm{blkdiag}[<z_{2}^{*}{>}_{1}\cdot (r_{1}^{H}⊗I_{M})\;<z_{2}^{*}{>}_{2}\cdot (r_{2}^{H}⊗I_{M})\;\cdots \;<z_{2}^{*}{>}_{N}\cdot (r_{N}^{H}⊗I_{M})]\cdot A(p)z_{1}\\ O_{MN\times 1} \end{array}\right]\;,\\ f_{3}[z_{1},z_{2}]={\left[z_{1}^{H}{\dot{A}}_{1}^{H}(p){\bar{X}}_{0}z_{2}\;z_{1}^{H}{\dot{A}}_{2}^{H}(p){\bar{X}}_{0}z_{2}\;\cdots \;z_{1}^{H}{\dot{A}}_{L}^{H}(p){\bar{X}}_{0}z_{2}\right]}^{H}\;, \end{array}\right.\;(\forall z_{1}\in C^{K\times 1}\;,\;z_{2}\in C^{N\times 1})\;.$$

The proof of (36) to (38) is provided in Appendix C. Secondly, substituting the second and third equalities in (29) into the second equality in (35) leads to

(39) $${\tilde{\lambda }}_{N}^{\left(2\right)}={\bar{\varepsilon }}_{c}^{H}H_{1}(p){\bar{\varepsilon }}_{c}+{\tilde{\varphi }}_{c}^{H}H_{2}(p){\tilde{\varphi }}_{c}+{\tilde{p}}^{T}H_{3}(p)\tilde{p}+{\bar{\varepsilon }}_{c}^{H}H_{4}(p){\tilde{\varphi }}_{c}+{\bar{\varepsilon }}_{c}^{H}H_{5}(p)\tilde{p}+{\tilde{\varphi }}_{c}^{H}H_{6}(p)\tilde{p}\;,$$

where

(40) $$\left\{ \begin{array}{cc} H_{1}(p) & =F_{a1}[B_{0}u_{N},U_{N}B_{0}^{H},u_{N}]+{\left(F_{a1}[B_{0}u_{N},U_{N}B_{0}^{H},u_{N}]\right)}^{H}+F_{b1}[u_{N},I_{K}+B_{0}U_{N}B_{0}^{H},u_{N}]\\ & \;+F_{c1}[B_{0}u_{N},U_{N},B_{0}u_{N}]\;,\\ H_{2}(p) & =F_{a2}[B_{0}u_{N},U_{N}B_{0}^{H},u_{N}]+{\left(F_{a2}[B_{0}u_{N},U_{N}B_{0}^{H},u_{N}]\right)}^{H}+F_{b2}[u_{N},I_{K}+B_{0}U_{N}B_{0}^{H},u_{N}]\\ & \;+F_{c2}[B_{0}u_{N},U_{N},B_{0}u_{N}]\;,\\ H_{3}(p) & =F_{a3}[B_{0}u_{N},U_{N}B_{0}^{H},u_{N}]+{\left(F_{a3}[B_{0}u_{N},U_{N}B_{0}^{H},u_{N}]\right)}^{H}+F_{b3}[u_{N},I_{K}+B_{0}U_{N}B_{0}^{H},u_{N}]\\ & \;+F_{c3}[B_{0}u_{N},U_{N},B_{0}u_{N}]+G_{3}[B_{0}u_{N},u_{N}]+{\left(G_{3}[B_{0}u_{N},u_{N}]\right)}^{*}\;,\\ H_{4}(p) & =F_{a4}[B_{0}u_{N},U_{N}B_{0}^{H},u_{N}]+\Pi_{\bar{\varepsilon }}{\left(F_{a4}[B_{0}u_{N},U_{N}B_{0}^{H},u_{N}]\right)}^{*}\Pi_{\tilde{\varphi }}+F_{b4}[u_{N},I_{K}+B_{0}U_{N}B_{0}^{H},u_{N}]\\ & \;+F_{c4}[B_{0}u_{N},U_{N},B_{0}u_{N}]\;,\\ H_{5}(p) & =F_{a5}[B_{0}u_{N},U_{N}B_{0}^{H},u_{N}]+\Pi_{\bar{\varepsilon }}{\left(F_{a5}[B_{0}u_{N},U_{N}B_{0}^{H},u_{N}]\right)}^{*}+F_{b5}[u_{N},I_{K}+B_{0}U_{N}B_{0}^{H},u_{N}]\\ & \;+F_{c5}[B_{0}u_{N},U_{N},B_{0}u_{N}]+G_{1}[B_{0}u_{N},u_{N}]+\Pi_{\bar{\varepsilon }}{\left(G_{1}[B_{0}u_{N},u_{N}]\right)}^{*}\;,\\ H_{6}(p) & =F_{a6}[B_{0}u_{N},U_{N}B_{0}^{H},u_{N}]+\Pi_{\tilde{\varphi }}{\left(F_{a6}[B_{0}u_{N},U_{N}B_{0}^{H},u_{N}]\right)}^{*}+F_{b6}[u_{N},I_{K}+B_{0}U_{N}B_{0}^{H},u_{N}]\\ & \;+F_{c6}[B_{0}u_{N},U_{N},B_{0}u_{N}]+G_{2}[B_{0}u_{N},u_{N}]+\Pi_{\tilde{\varphi }}{\left(G_{2}[B_{0}u_{N},u_{N}]\right)}^{*}\;, \end{array}\right.$$

in which ${\left\{F_{ak}[\cdot \;,\;\cdot ,\cdot ]\right\}}_{1\leq k\leq 6}$, ${\left\{F_{bk}[\cdot \;,\;\cdot ,\cdot ]\right\}}_{1\leq k\leq 6}$, ${\left\{F_{ck}[\cdot \;,\;\cdot ,\cdot ]\right\}}_{1\leq k\leq 6}$, and ${\left\{G_{k}[\cdot \;,\;\cdot ]\right\}}_{1\leq k\leq 3}$ can be viewed as matrix functions, which are given by

(41) $$\left\{ \begin{array}{l} F_{a1}[z_{1},Z,z_{2}]=\sum_{k=1}^{K}\Pi_{\bar{\varepsilon }}{\left(f_{1}[z_{1},Zi_{K}^{\left(k\right)}]\right)}^{*}{\left(f_{1}[i_{K}^{\left(k\right)},z_{2}]\right)}^{H}\;,\\ F_{a2}[z_{1},Z,z_{2}]=\sum_{k=1}^{K}\Pi_{\tilde{\varphi }}{\left(f_{2}[z_{1},Zi_{K}^{\left(k\right)}]\right)}^{*}{\left(f_{2}[i_{K}^{\left(k\right)},z_{2}]\right)}^{H}\;,\\ F_{a3}[z_{1},Z,z_{2}]=\sum_{k=1}^{K}{\left(f_{3}[z_{1},Zi_{K}^{\left(k\right)}]\right)}^{*}{\left(f_{3}[i_{K}^{\left(k\right)},z_{2}]\right)}^{H}\;,\\ F_{a4}[z_{1},Z,z_{2}]=\sum_{k=1}^{K}\Pi_{\bar{\varepsilon }}({\left(f_{1}[z_{1},Zi_{K}^{\left(k\right)}]\right)}^{*}{\left(f_{2}[i_{K}^{\left(k\right)},z_{2}]\right)}^{H}+{\left(f_{1}[i_{K}^{\left(k\right)},z_{2}]\right)}^{*}{\left(f_{2}[z_{1},Zi_{K}^{\left(k\right)}]\right)}^{H})\;,\\ F_{a5}[z_{1},Z,z_{2}]=\sum_{k=1}^{K}\Pi_{\bar{\varepsilon }}({\left(f_{1}[z_{1},Zi_{K}^{\left(k\right)}]\right)}^{*}{\left(f_{3}[i_{K}^{\left(k\right)},z_{2}]\right)}^{H}+{\left(f_{1}[i_{K}^{\left(k\right)},z_{2}]\right)}^{*}{\left(f_{3}[z_{1},Zi_{K}^{\left(k\right)}]\right)}^{H})\;,\\ F_{a6}[z_{1},Z,z_{2}]=\sum_{k=1}^{K}\Pi_{\tilde{\varphi }}({\left(f_{2}[z_{1},Zi_{K}^{\left(k\right)}]\right)}^{*}{\left(f_{3}[i_{K}^{\left(k\right)},z_{2}]\right)}^{H}+{\left(f_{2}[i_{K}^{\left(k\right)},z_{2}]\right)}^{*}{\left(f_{3}[z_{1},Zi_{K}^{\left(k\right)}]\right)}^{H})\;, \end{array}\right.\;(\forall z_{1}\in C^{K\times 1}\;,\;z_{2}\in C^{N\times 1}\;,\;Z\in C^{N\times K})\;,$$

(42) $$\left\{ \begin{array}{l} F_{b1}[z_{1},Z,z_{2}]=\sum_{k=1}^{K}f_{1}[Zi_{K}^{\left(k\right)},z_{1}]\cdot {\left(f_{1}[i_{K}^{\left(k\right)},z_{2}]\right)}^{H}\;,\\ F_{b2}[z_{1},Z,z_{2}]=\sum_{k=1}^{K}f_{2}[Zi_{K}^{\left(k\right)},z_{1}]\cdot {\left(f_{2}[i_{K}^{\left(k\right)},z_{2}]\right)}^{H}\;,\\ F_{b3}[z_{1},Z,z_{2}]=\sum_{k=1}^{K}f_{3}[Zi_{K}^{\left(k\right)},z_{1}]\cdot {\left(f_{3}[i_{K}^{\left(k\right)},z_{2}]\right)}^{H}\;,\\ F_{b4}[z_{1},Z,z_{2}]=\sum_{k=1}^{K}\left(f_{1}[Zi_{K}^{\left(k\right)},z_{1}]\cdot {\left(f_{2}[i_{K}^{\left(k\right)},z_{2}]\right)}^{H}+\Pi_{{\bar{\varepsilon }}_{c}}{\left(f_{1}[i_{K}^{\left(k\right)},z_{2}]\right)}^{*}{\left(f_{2}[Zi_{K}^{\left(k\right)},z_{1}]\right)}^{T}\Pi_{\tilde{\varphi }}\right)\;,\\ F_{b5}[z_{1},Z,z_{2}]=\sum_{k=1}^{K}\left(f_{1}[Zi_{K}^{\left(k\right)},z_{1}]\cdot {\left(f_{3}[i_{K}^{\left(k\right)},z_{2}]\right)}^{H}+\Pi_{{\bar{\varepsilon }}_{c}}{\left(f_{1}[i_{K}^{\left(k\right)},z_{2}]\right)}^{*}{\left(f_{3}[Zi_{K}^{\left(k\right)},z_{1}]\right)}^{T}\right)\;,\\ F_{b6}[z_{1},Z,z_{2}]=\sum_{k=1}^{K}\left(f_{2}[Zi_{K}^{\left(k\right)},z_{1}]\cdot {\left(f_{3}[i_{K}^{\left(k\right)},z_{2}]\right)}^{H}+\Pi_{\tilde{\varphi }}{\left(f_{2}[i_{K}^{\left(k\right)},z_{2}]\right)}^{*}{\left(f_{3}[Zi_{K}^{\left(k\right)},z_{1}]\right)}^{T}\right)\;, \end{array}\right.\;(\forall z_{1}\in C^{N\times 1}\;,\;z_{2}\in C^{N\times 1}\;,\;Z\in C^{K\times K})\;,$$

(43) $$\left\{ \begin{array}{l} F_{c1}[z_{1},Z,z_{2}]=\sum_{n=1}^{N}f_{1}[z_{2},i_{N}^{\left(n\right)}]\cdot {\left(f_{1}[z_{1},Zi_{N}^{\left(n\right)}]\right)}^{H}\;,\\ F_{c2}[z_{1},Z,z_{2}]=\sum_{n=1}^{N}f_{2}[z_{2},i_{N}^{\left(n\right)}]\cdot {\left(f_{2}[z_{1},Zi_{N}^{\left(n\right)}]\right)}^{H}\;,\\ F_{c3}[z_{1},Z,z_{2}]=\sum_{n=1}^{N}f_{3}[z_{2},i_{N}^{\left(n\right)}]\cdot {\left(f_{3}[z_{1},Zi_{N}^{\left(n\right)}]\right)}^{H}\;,\\ F_{c4}[z_{1},Z,z_{2}]=\sum_{n=1}^{N}\left(f_{1}[z_{2},i_{N}^{\left(n\right)}]\cdot {\left(f_{2}[z_{1},Zi_{N}^{\left(n\right)}]\right)}^{H}+\Pi_{{\bar{\varepsilon }}_{c}}{\left(f_{1}[z_{1},Zi_{N}^{\left(n\right)}]\right)}^{*}{\left(f_{2}[z_{2},i_{N}^{\left(n\right)}]\right)}^{T}\Pi_{{\tilde{\varphi }}_{c}}\right)\;,\\ F_{c5}[z_{1},Z,z_{2}]=\sum_{n=1}^{N}\left(f_{1}[z_{2},i_{N}^{\left(n\right)}]\cdot {\left(f_{3}[z_{1},Zi_{N}^{\left(n\right)}]\right)}^{H}+\Pi_{{\bar{\varepsilon }}_{c}}{\left(f_{1}[z_{1},Zi_{N}^{\left(n\right)}]\right)}^{*}{\left(f_{3}[z_{2},i_{N}^{\left(n\right)}]\right)}^{T}\right)\;,\\ F_{c6}[z_{1},Z,z_{2}]=\sum_{n=1}^{N}\left(f_{2}[z_{2},i_{N}^{\left(n\right)}]\cdot {\left(f_{3}[z_{1},Zi_{N}^{\left(n\right)}]\right)}^{H}+\Pi_{{\tilde{\varphi }}_{c}}{\left(f_{2}[z_{1},Zi_{N}^{\left(n\right)}]\right)}^{*}{\left(f_{3}[z_{2},i_{N}^{\left(n\right)}]\right)}^{T}\right)\;, \end{array}\right.\;(\forall z_{1}\in C^{K\times 1}\;,\;z_{2}\in C^{K\times 1}\;,\;Z\in C^{N\times N})\;,$$

(44) $$\left\{ \begin{array}{l} G_{1}[z_{1},z_{2}]=\left[\begin{array}{c} O_{MNK\times L}\\ \mathrm{diag}[z_{2}⊗1_{MK\times 1}]\cdot [{\dot{A}}_{1}^{*}(p)z_{1}^{*}\;{\dot{A}}_{2}^{*}(p)z_{1}^{*}\;\cdots \;{\dot{A}}_{L}^{*}(p)z_{1}^{*}] \end{array}\right]\;,\\ G_{2}[z_{1},z_{2}]=\left[\begin{array}{c} O_{MN\times L}\\ \begin{array}{c} \mathrm{blkdiag}[<z_{2}{>}_{1}\cdot (r_{1}^{T}⊗I_{M})\;<z_{2}{>}_{2}\cdot (r_{2}^{T}⊗I_{M})\;\cdots \;<z_{2}{>}_{N}\cdot (r_{N}^{T}⊗I_{M})]\\ \times [{\dot{A}}_{1}^{*}(p)z_{1}^{*}\;{\dot{A}}_{2}^{*}(p)z_{1}^{*}\;\cdots \;{\dot{A}}_{L}^{*}(p)z_{1}^{*}] \end{array} \end{array}\right]\;,\\ G_{3}[z_{1},z_{2}]=\frac{1}{2}\cdot \left[\begin{array}{cccc} z_{1}^{H}{\ddot{A}}_{11}^{H}(p){\bar{X}}_{0}z_{2} & z_{1}^{H}{\ddot{A}}_{12}^{H}(p){\bar{X}}_{0}z_{2} & \cdots & z_{1}^{H}{\ddot{A}}_{1L}^{H}(p){\bar{X}}_{0}z_{2}\\ z_{1}^{H}{\ddot{A}}_{21}^{H}(p){\bar{X}}_{0}z_{2} & z_{1}^{H}{\ddot{A}}_{22}^{H}(p){\bar{X}}_{0}z_{2} & \cdots & z_{1}^{H}{\ddot{A}}_{2L}^{H}(p){\bar{X}}_{0}z_{2}\\ \vdots & \vdots & \ddots & \vdots \\ z_{1}^{H}{\ddot{A}}_{L1}^{H}(p){\bar{X}}_{0}z_{2} & z_{1}^{H}{\ddot{A}}_{L2}^{H}(p){\bar{X}}_{0}z_{2} & \cdots & z_{1}^{H}{\ddot{A}}_{LL}^{H}(p){\bar{X}}_{0}z_{2} \end{array}\right]\;, \end{array}\right.\;(\forall z_{1}\in C^{K\times 1}\;,\;z_{2}\in C^{K\times 1})\;.$$

The proof of (39) to (44) is provided in Appendix D. Substituting (36) and (39) back into (34) yields

(45) $$\begin{array}{cc} \hat{J}(\hat{p}) & =\lambda_{\max}\{{\hat{B}}^{H}(\hat{p})\hat{B}(\hat{p})\}\approx \lambda_{N}+h_{1}^{H}(p){\bar{\varepsilon }}_{c}+h_{2}^{H}(p){\tilde{\varphi }}_{c}+h_{3}^{T}(p)\tilde{p}\\ & +{\bar{\varepsilon }}_{c}^{H}H_{1}(p){\bar{\varepsilon }}_{c}+{\tilde{\varphi }}_{c}^{H}H_{2}(p){\tilde{\varphi }}_{c}+{\tilde{p}}^{T}H_{3}(p)\tilde{p}+{\bar{\varepsilon }}_{c}^{H}H_{4}(p){\tilde{\varphi }}_{c}+{\bar{\varepsilon }}_{c}^{H}H_{5}(p)\tilde{p}+{\tilde{\varphi }}_{c}^{H}H_{6}(p)\tilde{p}\;. \end{array}$$

Evidently, Equation (45) can be considered as the second-order perturbation expression with respect to the error vectors ${\bar{\varepsilon }}_{c}$, ${\tilde{\varphi }}_{c}$, and $\tilde{p}$. From (45), we get the linear relationship between the localization error $\tilde{p}$ and sensor noise ${\bar{\varepsilon }}_{c}$ as well as array model error ${\tilde{\varphi }}_{c}$. The MSE of the DPD estimator can then be derived according to the statistical assumptions on the two sources of error.

### 6.3. MSE of Direct Position Determination Method

In light of the maximum principle, the true position p and estimated position $\hat{p}$ satisfy the relations

(46) $$\left\{ \begin{array}{l} {\frac{\partial \hat{J}(p)}{\partial p}|}_{\begin{array}{l} \bar{\varepsilon }=O_{MNK\times 1}\\ \tilde{\varphi }=O_{MN\times 1} \end{array}}=O_{L\times 1}\;,\\ \frac{\partial \hat{J}(\hat{p})}{\partial \hat{p}}=O_{L\times 1}\;. \end{array}\right.$$

Obviously, the first equality in (46) leads to

(47) ∂J^(p)∂p|ε¯=OMNK×1φ˜=OMN×1=h3(p)=2⋅Re{f3[B0uN,uN]}=OL×1 .

Additionally, using (45) and the second equality in (46), the localization error $\tilde{p}=\hat{p}-p$ is obtained by

(48) $$\begin{array}{cc} \tilde{p} & =\arg\underset{z\in R^{L\times 1}}{\max}\left\{\begin{array}{c} h_{1}^{H}(p){\bar{\varepsilon }}_{c}+h_{2}^{H}(p){\tilde{\varphi }}_{c}+h_{3}^{T}(p)z+{\bar{\varepsilon }}_{c}^{H}H_{1}(p){\bar{\varepsilon }}_{c}+{\tilde{\varphi }}_{c}^{H}H_{2}(p){\tilde{\varphi }}_{c}\\ +z^{T}H_{3}(p)z+{\bar{\varepsilon }}_{c}^{H}H_{4}(p){\tilde{\varphi }}_{c}+{\bar{\varepsilon }}_{c}^{H}H_{5}(p)z+{\tilde{\varphi }}_{c}^{H}H_{6}(p)z \end{array}\right\}\\ & =\arg\underset{z\in R^{L\times 1}}{\max}\{h_{3}^{T}(p)z+z^{T}H_{3}(p)z+{\bar{\varepsilon }}_{c}^{H}H_{5}(p)z+{\tilde{\varphi }}_{c}^{H}H_{6}(p)z\}\;, \end{array}$$

which further implies

(49) $$\begin{array}{cc} \tilde{p} & =-\frac{1}{2}H_{3}^{-1}(p)H_{5}^{T}(p){\bar{\varepsilon }}_{c}^{*}-\frac{1}{2}H_{3}^{-1}(p)H_{6}^{T}(p){\tilde{\varphi }}_{c}^{*}-\frac{1}{2}H_{3}^{-1}(p)h_{3}(p)\\ & =-\frac{1}{2}H_{3}^{-1}(p)H_{5}^{T}(p){\bar{\varepsilon }}_{c}^{*}-\frac{1}{2}H_{3}^{-1}(p)H_{6}^{T}(p){\tilde{\varphi }}_{c}^{*}=O\left({\left\|\left[\begin{array}{c} \bar{\varepsilon }\\ \tilde{\varphi } \end{array}\right]\right\|}_{2}\right)\;. \end{array}$$

The second equality in (49) follows from (47). In (49), the linear relationship between the localization error $\tilde{p}$ and the sensor noise ${\bar{\varepsilon }}_{c}$ as well as the array model error ${\tilde{\varphi }}_{c}$ is formulated. It is easily observed from (49) that the positioning error vector $\tilde{p}$ consists of two terms. The first term is associated with the sensor noise, which can be described as

(50) $${\tilde{p}}_{1}=-\frac{1}{2}H_{3}^{-1}(p)H_{5}^{T}(p){\bar{\varepsilon }}_{c}^{*}=O(||\bar{\varepsilon }|{|}_{2})\;.$$

The second term is due to the array model errors, which can be written as

(51) $${\tilde{p}}_{2}=-\frac{1}{2}H_{3}^{-1}(p)H_{6}^{T}(p){\tilde{\varphi }}_{c}^{*}=O(||\tilde{\varphi }|{|}_{2})\;.$$

According to the statistical assumptions in Section 3 and Section 5, it is concluded that the localization error $\tilde{p}$ is asymptotically Gaussian distributed with a zero mean and a covariance matrix given by

(52) $$P=E[\tilde{p}{\tilde{p}}^{T}]=\frac{1}{4}H_{3}^{-1}(p)H_{5}^{T}(p)\cdot E[{\bar{\varepsilon }}_{c}^{*}{\bar{\varepsilon }}_{c}^{H}]\cdot H_{5}(p)H_{3}^{-T}(p)+\frac{1}{4}H_{3}^{-1}(p)H_{6}^{T}(p)\cdot E[{\tilde{\varphi }}_{c}^{*}{\tilde{\varphi }}_{c}^{H}]\cdot H_{6}(p)H_{3}^{-T}(p)\;,$$

where the second equality follows from (49) and the fact that ${\bar{\varepsilon }}_{c}$ and ${\tilde{\varphi }}_{c}$ are statistically independent. Furthermore, (4), (14), and (23) together imply that

(53) $$E[{\bar{\varepsilon }}_{c}^{*}{\bar{\varepsilon }}_{c}^{H}]=\left[\begin{array}{cc} O_{MNK\times MNK} & \sigma_{\varepsilon }^{2}I_{MNK}\\ \sigma_{\varepsilon }^{2}I_{MNK} & O_{MNK\times MNK} \end{array}\right]\;,\;E[{\tilde{\varphi }}_{c}^{*}{\tilde{\varphi }}_{c}^{H}]=\left[\begin{array}{cc} \mathrm{blkdiag}[\Phi_{1}^{(1)*}\;\Phi_{2}^{(1)*}\;\cdots \;\Phi_{N}^{(1)*}] & \mathrm{blkdiag}[\Phi_{1}^{(2)*}\;\Phi_{2}^{(2)*}\;\cdots \;\Phi_{N}^{(2)*}]\\ \mathrm{blkdiag}[\Phi_{1}^{\left(2\right)}\;\Phi_{2}^{\left(2\right)}\;\cdots \;\Phi_{N}^{\left(2\right)}] & \mathrm{blkdiag}[\Phi_{1}^{\left(1\right)}\;\Phi_{2}^{\left(1\right)}\;\cdots \;\Phi_{N}^{\left(1\right)}] \end{array}\right]\;.$$

Inserting (53) back into (52) leads to

(54) $$\begin{array}{cc} P & =\frac{1}{4}H_{3}^{-1}(p)H_{5}^{T}(p)\cdot \left[\begin{array}{cc} O_{MNK\times MNK} & \sigma_{\varepsilon }^{2}I_{MNK}\\ \sigma_{\varepsilon }^{2}I_{MNK} & O_{MNK\times MNK} \end{array}\right]\cdot H_{5}(p)H_{3}^{-T}(p)\\ & +\frac{1}{4}H_{3}^{-1}(p)H_{6}^{T}(p)\cdot \left[\begin{array}{cc} \mathrm{blkdiag}[\Phi_{1}^{(1)*}\;\Phi_{2}^{(1)*}\;\cdots \;\Phi_{N}^{(1)*}] & \mathrm{blkdiag}[\Phi_{1}^{(2)*}\;\Phi_{2}^{(2)*}\;\cdots \;\Phi_{N}^{(2)*}]\\ \mathrm{blkdiag}[\Phi_{1}^{\left(2\right)}\;\Phi_{2}^{\left(2\right)}\;\cdots \;\Phi_{N}^{\left(2\right)}] & \mathrm{blkdiag}[\Phi_{1}^{\left(1\right)}\;\Phi_{2}^{\left(1\right)}\;\cdots \;\Phi_{N}^{\left(1\right)}] \end{array}\right]\cdot H_{6}(p)H_{3}^{-T}(p)\;. \end{array}$$

From (54) we see that the covariance matrix P is composed of two parts. The first part, due to the sensor noises, is expressed as

(55) $$P_{1}=\frac{1}{4}H_{3}^{-1}(p)H_{5}^{T}(p)\cdot \left[\begin{array}{cc} O_{MNK\times MNK} & \sigma_{\varepsilon }^{2}I_{MNK}\\ \sigma_{\varepsilon }^{2}I_{MNK} & O_{MNK\times MNK} \end{array}\right]\cdot H_{5}(p)H_{3}^{-T}(p)\;.$$

The second part, due to the array model errors, is given by

(56) $$P_{2}=\frac{1}{4}H_{3}^{-1}(p)H_{6}^{T}(p)\cdot \left[\begin{array}{cc} \mathrm{blkdiag}[\Phi_{1}^{(1)*}\;\Phi_{2}^{(1)*}\;\cdots \;\Phi_{N}^{(1)*}] & \mathrm{blkdiag}[\Phi_{1}^{(2)*}\;\Phi_{2}^{(2)*}\;\cdots \;\Phi_{N}^{(2)*}]\\ \mathrm{blkdiag}[\Phi_{1}^{\left(2\right)}\;\Phi_{2}^{\left(2\right)}\;\cdots \;\Phi_{N}^{\left(2\right)}] & \mathrm{blkdiag}[\Phi_{1}^{\left(1\right)}\;\Phi_{2}^{\left(1\right)}\;\cdots \;\Phi_{N}^{\left(1\right)}] \end{array}\right]\cdot H_{6}(p)H_{3}^{-T}(p)\;.$$

**Remark** **1.** *It is evident that the trace of* P *can be viewed as the MSE of localization errors under the combined effects of sensor noise and array model errors.*

**Remark** **2.** *When* $\Phi_{n}^{\left(1\right)}\to O$ *and* $\Phi_{n}^{\left(2\right)}\to O$*, the trace of* P *can be viewed as the MSE of the localization errors when no array model errors are present. Moreover, the value of the trace of* P *approaches the CRB for the case of none of array model errors, which will be shown in Section 8.1. This is because the DPD method studied here is derived from the maximum likelihood (ML) criterion, which provides an asymptotically efficient solution.*

**Remark** **3.** *When* $\sigma_{\varepsilon }^{2}\to 0$*, the trace of* P *can be used to quantify the sensitivity of positioning accuracy to array model errors, and represents the additional estimation errors resulting from uncertainties in the array manifold.*

**Remark** **4.** *It is easily seen from (55) and (56) that both* $P_{1}$ *and* $P_{2}$ *rely on matrix* $H_{3}(p)$*, which is the* p*-corner of the Hessian matrix of the cost function. If this matrix has a large condition number, the positioning accuracy might be high and, conversely, if this matrix is nearly singular, the location error may be extremely large.*

**Remark** **5.** *From (54), it is observed that covariance matrix* P *is related to* $H_{3}(p)$, $H_{5}(p)$*, and* $H_{6}(p)$*. According to (38) and (40)–(44), the ijth element of matrix* $H_{3}(p)$ *is given by*

(57) <H3(p)>ij=uNH(X¯0HA˙i(p)B0UNX¯0HA˙j(p)B0+B0HA˙iH(p)X¯0UNB0HA˙jH(p)X¯0+B0HA˙jH(p)X¯0UNX¯0HA˙i(p)B0+X¯0HA˙i(p)(IK+B0UNB0H)A˙jH(p)X¯0)uN+Re{uNHB0HA¨ijH(p)X¯0uN} .

In addition, the expressions for matrices $H_{5}(p)$ and $H_{6}(p)$ can be obtained from (38) and (40)–(44). However, the two formulas are complicated and we therefore have to omit them because of space limitations.

## 7. Success Probability of Direct Position Determination Method in Presence of Array Model Errors

The aim of this section is to deduce the success probability (SP) of the DPD method when array model errors exist. Two quantitative criterions are introduced to justify whether the localization is successful. Additionally, two analytical expressions for the SP of positioning are derived.

### 7.1. The First Success Probability of Direct Position Determination

**Definition** **1.** *If condition “*$|<\tilde{p}{>}_{1}|\leq \Delta_{1}\;,\;|<\tilde{p}{>}_{2}|\leq \Delta_{2}\;,\;\cdots \;,\;|<\tilde{p}{>}_{L}|\leq \Delta_{L}$*” is satisfied, then the localization is successful.*

It must be emphasized that the set of parameters ${\left\{\Delta_{l}\right\}}_{1\leq l\leq L}$ in Definition 1 shall be appropriately chosen according to the practical scenario. The difference in these parameters reflects the importance of localization accuracy in distinct orientation. If the importance for each direction is identical, then these parameters can be set to the same value.

According to Definition 1, the joint probability density function of positioning error vector $\tilde{p}$ is required for the calculation of the first localization SP. Applying the results in Section 6.3, the probability density function of random vector $\tilde{p}$ is given by

(58) $$f_{\tilde{p}}(z)={\left(2\pi \right)}^{-L/2}\cdot |\det[P]{|}^{-1/2}\cdot \exp\{-z^{T}P^{-1}z/2\}\;.$$

Consequently, the first localization SP can be determined by

(59) $$\begin{array}{l} \mathrm{Pr}\{|<\tilde{p}{>}_{1}|\leq \Delta_{1}\;,\;|<\tilde{p}{>}_{2}|\leq \Delta_{2}\;,\;\cdots \;,\;|<\tilde{p}{>}_{L}|\leq \Delta_{L}\}\\ =\int_{-\Delta_{L}}^{\Delta_{L}}\cdots \int_{-\Delta_{2}}^{\Delta_{2}}\int_{-\Delta_{1}}^{\Delta_{1}}{\left(2\pi \right)}^{-L/2}\cdot |\det[P]{|}^{-1/2}\cdot \exp\{-z^{T}P^{-1}z/2\}\;dz_{1}dz_{2}\cdots dz_{L}\;. \end{array}$$

It is apparent from (59) that the first SP can be approximately obtained via numerical integration over a cube in high dimensional Euclidean space.

However, the high-dimensional numerical integration is not attractive from a computational viewpoint. If possible, it is preferable to get an explicit formula. Obviously, this is a non-trivial task and we only consider two-dimensional (2-D) localization scenarios (i.e., L=2) for simplicity of mathematical analysis. First, an explicit formula with which to evaluate the joint probability of the Gaussian distribution is formally concluded in a proposition as below.

**Proposition** **2.** *Consider two joint Gaussian random variables* $z_{1}$ *and* $z_{2}$*. The mean and variance of* $z_{1}$ *are* $m_{1}$ *and* $v_{11}$*, respectively. The mean and variance of* $z_{2}$ *are* $m_{2}$ *and* $v_{22}$*, respectively. In addition, the covariance of the two random variables is* $v_{12}$*. It follows that*

(60) $$\mathrm{Pr}\{z_{1}\leq \alpha_{1}\;,\;z_{2}\leq \alpha_{2}\}=\Gamma_{0}[\alpha_{10}/\sqrt{v_{11}}]\cdot \Gamma_{0}[(\alpha_{2}-E[{\bar{z}}_{2}])\;/\sqrt{\mathrm{var}[{\bar{z}}_{2}]}]\;,$$

where

(61) $$\left\{ \begin{array}{c} E[{\bar{z}}_{2}]=m_{2}-\frac{v_{12}\cdot \exp\{-\alpha_{10}^{2}/(2v_{11})\}}{\sqrt{2\pi v_{11}}\cdot \Gamma_{0}[\alpha_{10}/\sqrt{v_{11}}]}\\ \mathrm{var}[{\bar{z}}_{2}]=v_{22}-\frac{v_{12}^{2}}{\sqrt{2\pi }v_{11}\cdot \Gamma_{0}[\alpha_{10}/\sqrt{v_{11}}]}\cdot \left(\frac{\alpha_{10}\cdot \exp\{-\alpha_{10}^{2}/(2v_{11})\}}{\sqrt{v_{11}}}+\frac{\exp\{-\alpha_{10}^{2}/v_{11}\}}{\sqrt{2\pi }\cdot \Gamma_{0}[\alpha_{10}/\sqrt{v_{11}}]}\;\right) \end{array}\right.$$

with $\alpha_{10}=\alpha_{1}-m_{1}$ and $\Gamma_{0}[x]=\int_{-\infty }^{x}\frac{1}{\sqrt{2\pi }}\cdot \exp\{-t^{2}/2\}\cdot dt$.

Appendix E shows the proof of Proposition 2, which is along the lines of incomplete conditional moments theory presented in [46]. Note that Proposition 2 plays a significant role in the subsequent derivation process.

When L=2, it can be verified by algebraic manipulation that

(62) $$\begin{array}{l} \mathrm{Pr}\{|<\tilde{p}{>}_{1}|\leq \Delta_{1}\;,\;|<\tilde{p}{>}_{2}|\leq \Delta_{2}\}\\ =\mathrm{Pr}\{-\Delta_{1}\leq \;<\tilde{p}{>}_{1}\;\leq \Delta_{1}\}-\mathrm{Pr}\{<\tilde{p}{>}_{2}\;\geq \Delta_{2}\}-\mathrm{Pr}\{<\tilde{p}{>}_{2}\;\leq -\Delta_{2}\}+\mathrm{Pr}\{-<\tilde{p}{>}_{1}\;\leq -\Delta_{1}\;,\;-<\tilde{p}{>}_{2}\;\leq -\Delta_{2}\}\\ +\mathrm{Pr}\{<\tilde{p}{>}_{1}\;\leq -\Delta_{1}\;,\;-<\tilde{p}{>}_{2}\;\leq -\Delta_{2}\}+\mathrm{Pr}\{-<\tilde{p}{>}_{1}\;\leq -\Delta_{1}\;,\;<\tilde{p}{>}_{2}\;\leq -\Delta_{2}\}+\mathrm{Pr}\{<\tilde{p}{>}_{1}\;\leq -\Delta_{1}\;,\;<\tilde{p}{>}_{2}\;\leq -\Delta_{2}\}\;. \end{array}$$

The proof of (62) is shown in Appendix F. Applying the result in Proposition 2 and the definition of $\Gamma_{0}[x]$, we have

(63) $$\begin{array}{l} \mathrm{Pr}\{|<\tilde{p}{>}_{1}|\leq \Delta_{1}\;,\;|<\tilde{p}{>}_{2}|\leq \Delta_{2}\}\\ =\Gamma_{0}[\Delta_{1}/\sqrt{<P{>}_{11}}]-\Gamma_{0}[-\Delta_{1}/\sqrt{<P{>}_{11}}]-2\cdot \Gamma_{0}[-\Delta_{2}/\sqrt{<P{>}_{22}}]\\ +2\cdot \Gamma_{0}[-\Delta_{1}/\sqrt{<P{>}_{11}}]\cdot (\Gamma_{0}[(-\Delta_{2}+\kappa_{1})/\sqrt{\kappa_{2}}]+\Gamma_{0}[(-\Delta_{2}-\kappa_{1})/\sqrt{\kappa_{2}}])\;, \end{array}$$

where

(64) $$\left\{ \begin{array}{c} \kappa_{1}=\frac{<P{>}_{12}\cdot \exp\{-\Delta_{1}^{2}/(2\cdot <P{>}_{11})\}}{\sqrt{2\pi \cdot <P{>}_{11}}\cdot \Gamma_{0}[-\Delta_{1}/\sqrt{<P{>}_{11}}]}\;,\\ \kappa_{2}=<P{>}_{22}-\frac{{\left(<P{>}_{12}\right)}^{2}}{\sqrt{2\pi }\cdot <P{>}_{11}\cdot \Gamma_{0}[-\Delta_{1}/\sqrt{<P{>}_{11}}]}\cdot \left(\frac{-\Delta_{1}\cdot \exp\{-\Delta_{1}^{2}/(2\cdot <P{>}_{11})\}}{\sqrt{<P{>}_{11}}}+\frac{\exp\{-\Delta_{1}^{2}/<P{>}_{11}\}}{\sqrt{2\pi }\cdot \Gamma_{0}[-\Delta_{1}/\sqrt{<P{>}_{11}}]}\;\right)\;. \end{array}\right.$$

**Remark** **6.** *The value of* $\Gamma_{0}[x]$ *for arbitrary* x *is available from a table given in a textbook on probability theory.*

**Remark** **7.** It must be pointed out that the above analytical results cannot be directly applied to the three-dimensional (3-D) case; i.e., L=3. This can even be regarded as an open problem. Nevertheless, we can use numerical methods to compute this kind of SP in 3-D space. Indeed, there exist a number of efficient numerical integration methods with which to calculate the probability in (59), such as the Richardson extrapolation algorithm, Simpson algorithm, and Monte Carlo algorithm.

### 7.2. The Second Success Probability of Direct Position Determination

**Definition** **2.** *If condition “*$\sqrt{\frac{1}{L}\sum_{l=1}^{L}<\tilde{p}{>}_{l}^{2}}\leq \Delta$*” is satisfied, then the localization is successful.*

It is readily seen from Definition 2 that the second SP of positioning is equal to $\mathrm{Pr}\;\{||\tilde{p}||{\;}_{2}^{2}\leq L\Delta^{2}\}$. To proceed, let us express $\tilde{p}$ as $\tilde{p}\overset{d}{=}P^{1/2}{\tilde{p}}_{0}$, where ${\tilde{p}}_{0}$ is a zero-mean Gaussian random vector with covariance matrix $I_{L}$, and $\overset{d}{=}$ indicates that both sides have the same probability distribution. Consequently, $||\tilde{p}||{\;}_{2}^{2}$ can be formulated as the quadratic form of ${\tilde{p}}_{0}$:

(65) $$||\tilde{p}||{\;}_{2}^{2}\overset{d}{=}{\tilde{p}}_{0}^{T}P{\tilde{p}}_{0}\;.$$

In light of the relationship between the cumulative distribution function and characteristic function [64], we have

(66) Pr{||p˜|| 22≤LΔ2}=12−1π⋅∫0+∞1t⋅Im{exp{−jLΔ2t}⋅φ||p˜|| 22(t)} dt ,

where $\varphi_{{||\tilde{p}||\;}_{2}^{2}}(t)$ denotes the characteristic function of $||\tilde{p}||{\;}_{2}^{2}$. Suppose that matrix P has eigenvalues $\gamma_{1}\;,\;\gamma_{2}\;,\;\cdots \;,\;\gamma_{L}$. Applying the property of the characteristic function, it can be proved that

(67) $$\varphi_{{||\tilde{p}||\;}_{2}^{2}}(t)=\prod_{l=1}^{L}{\left(1+4\gamma_{l}^{2}\;t^{2}\right)}^{-1/4}\cdot \exp\{j(\arctan(2\gamma_{l}\;t)/2)\}\;.$$

The substitution of (67) into (66) produces

(68) $$\mathrm{Pr}\{||\tilde{p}||{\;}_{2}^{2}\leq L\Delta^{2}\}=\frac{1}{2}-\frac{1}{\pi }\cdot \int_{0}^{+\infty }\frac{1}{t}\cdot \frac{\sin(\delta_{1}(t))}{\delta_{2}(t)}dt\;,$$

where

(69) $$\left\{ \begin{array}{l} \delta_{1}(t)=\sum_{l=1}^{L}\arctan(2\gamma_{l}t)/2-L\Delta^{2}t\;,\\ \delta_{2}(t)=\prod_{l=1}^{L}{\left(1+4\gamma_{l}^{2}t^{2}\right)}^{1/4}\;. \end{array}\right.$$

**Remark** **8.** *It is clear from (68) that a one-dimension numerical integration over* [0 , +∞) *is required to evaluate the second SP. To this end, the values of the integrand shall be analyzed as* t→0 *and* t→+∞.

**Remark** **9.** Applying L’Hospital’s rule leads to

(70) $$\underset{t\to 0}{\lim}\frac{1}{t}\cdot \frac{\sin\{\delta_{1}(t)\}}{\delta_{2}(t)}=\underset{t\to 0}{\lim}\frac{\cos\{\delta_{1}(t)\}\cdot {\dot{\delta }}_{1}(t)}{\delta_{2}(t)+t{\dot{\delta }}_{2}(t)}={\dot{\delta }}_{1}(0)=\sum_{l=1}^{L}\gamma_{l}-L\Delta^{2}\;.$$

**Remark** **10.** *The numerator of the integrand is bounded and the denominator tends to infinity when* t→+∞ *and, therefore, the integrand will be arbitrarily close to zero when* t→+∞*. The integral upper limit in (68) can then be replaced by a sufficiently large positive number for the sake of simplicity.*

**Remark** **11.** It can be rigorously proved that the first SP is always smaller than the second SP, provided that $\Delta_{1}=\cdots =\Delta_{L}=\Delta$. *The reason is that the first probability is computed by the numerical integral over a cube, while the second probability is equal to the integral over a circumscribed sphere of the cube.*

As a byproduct of (68), we can present a new method of determining the radius of circular error probable (CEP), which is first defined in [65]. We denote $r_{\mathrm{CEP}}$ by the radius of CEP, and it follows from its definition and (68) that

(71) $$\frac{1}{2}=\mathrm{Pr}\{||\tilde{p}||{\;}_{2}^{2}\leq r_{\mathrm{CEP}}^{2}\}=\frac{1}{2}-\frac{1}{\pi }\cdot \int_{0}^{+\infty }\frac{1}{t}\cdot \frac{\sin\left(\sum_{l=1}^{L}\arctan(2\gamma_{l}t)/2-r_{\mathrm{CEP}}^{2}t\right)}{\prod_{l=1}^{L}{\left(1+4\gamma_{l}^{2}t^{2}\right)}^{1/4}}dt\;,$$

which implies that

(72) $$\int_{0}^{+\infty }\frac{1}{t}\cdot \frac{\sin\left(\sum_{l=1}^{L}\arctan(2\gamma_{l}t)/2-r_{\mathrm{CEP}}^{2}t\right)}{\prod_{l=1}^{L}{\left(1+4\gamma_{l}^{2}t^{2}\right)}^{1/4}}dt=0\;.$$

As a consequence, a reasonable criterion for calculating $r_{\mathrm{CEP}}$ is given by

(73) $$\underset{x}{\min}{\left(\int_{0}^{+\infty }\frac{1}{t}\cdot \frac{\sin\left(\sum_{l=1}^{L}\arctan(2\gamma_{l}t)/2-x^{2}t\right)}{\prod_{l=1}^{L}{\left(1+4\gamma_{l}^{2}t^{2}\right)}^{1/4}}dt\right)}^{2}\;,$$

which can be solved via a one-dimensional grid search. In addition, it is noteworthy that although the solution for estimating $r_{\mathrm{CEP}}$ is presented in [65], it is only applicable to 2-D localization scenarios. In contrast, the method proposed here is suitable for not only 2-D localization but also the 3-D scenario.

## 8. Cramér-Rao Bound on Covariance Matrix of Localization Errors

The CRB is a commonly used lower bound on the estimation error covariance of any unbiased estimator. In other words, the difference between the covariance and the CRB is a positive semi-definite matrix. Moreover, the CRB is expected to be a good predictor for the performance of the maximum likelihood estimator (MLE) at a moderate noise level. In this section, we derive the CRB for the estimate of the transmitter’s position in two cases: (1) array model errors are absent and (2) array model errors are present. To this end, we first introduce the following proposition whose proof can be found in [66].

**Proposition** **3.** *Assuming that the CRB matrix for the real vector* z *is equal to* CRB(z)*, and defining a novel real vector as* $z^{\prime }=Jz$*, where* J *is an invertible matrix, the CRB matrix for vector* $z^{\prime }$ *is given by* $\mathrm{CRB}(z^{\prime })=J\cdot \mathrm{CRB}(z)\cdot J^{T}$.

### 8.1. Cramér-Rao Bound on Position Estimate in Absence of Array Model Errors

This subsection is devoted to deriving the CRB for localization in the absence of array model errors. We begin by introducing a parameter vector that gathers all unknowns

(74) ηa=[σε2  pT  (Re{β})T  (Im{β})T  (Re{s¯})T  (Im{s¯})T]T=[σε2  μaT]T ,

where

(75) μa=[pT  βrT  (Re{s¯})T  (Im{s¯})T]T

with βr=[(Re{β})T  (Im{β})T]T. To proceed, the data vector is defined as

(76) $$\bar{x}={\left[{\bar{x}}_{1}^{H}\;{\bar{x}}_{2}^{H}\;\cdots \;{\bar{x}}_{N}^{H}\right]}^{H}={\left[{\bar{x}}_{1,1}^{H}\;{\bar{x}}_{1,2}^{H}\;\cdots \;{\bar{x}}_{1,K}^{H}\;{\bar{x}}_{2,1}^{H}\;{\bar{x}}_{2,2}^{H}\;\cdots \;{\bar{x}}_{2,K}^{H}\;\cdots \;\cdots \;{\bar{x}}_{N,1}^{H}\;{\bar{x}}_{N,2}^{H}\;\cdots \;{\bar{x}}_{N,K}^{H}\right]}^{H}\;,$$

whose mean vector is given by

(77) $${\bar{x}}_{0}=E[\bar{x}]={\left[{\bar{x}}_{1,1,0}^{H}\;{\bar{x}}_{1,2,0}^{H}\;\cdots \;{\bar{x}}_{1,K,0}^{H}\;{\bar{x}}_{2,1,0}^{H}\;{\bar{x}}_{2,2,0}^{H}\;\cdots \;{\bar{x}}_{2,K,0}^{H}\;\cdots \;{\bar{x}}_{N,1,0}^{H}\;{\bar{x}}_{N,2,0}^{H}\;\cdots \;{\bar{x}}_{N,K,0}^{H}\right]}^{H}\;.$$

Then, applying the results in [66,67], the CRB matrix for vector $\mu_{a}$ can be obtained by

(78) CRB(μa)=σε22⋅(Re{ΩμaHΩμa})−1 ,

where

(79) Ωμa=∂x¯0∂μaT=[Ωp  ΩRe{β}  ΩIm{β}  ΩRe{s¯}  ΩIm{s¯}] .

Using (16) and (77) and performing algebraic manipulations, the sub-matrices in (79) are described as

(80) {Ωp=∂x¯0∂pT=diag[β⊗1MK×1]⋅diag[1N×1⊗s¯⊗1M×1]⋅∂a′(p)∂pT ,ΩRe{β}=∂x¯0∂(Re{β})T=diag[1N×1⊗s¯⊗1M×1]⋅blkdiag[a1′(p)  a2′(p)  ⋯  aN′(p)] ,ΩIm{β}=∂x¯0∂(Im{β})T=j⋅∂x¯0∂(Re{β})T=j⋅diag[1N×1⊗s¯⊗1M×1]⋅blkdiag[a1′(p)  a2′(p)  ⋯  aN′(p)] ,ΩRe{s¯}=∂x¯0∂(Re{s¯})T=diag[β⊗1MK×1]⋅A(p) ,ΩIm{s¯}=∂x¯0∂(Im{s¯})T=j⋅∂x¯0∂(Re{s¯})T=j⋅diag[β⊗1MK×1]⋅A(p) ,

where

(81) $$\left\{ \begin{array}{c} a^{\prime }(p)={\left[a_{1}^{\prime H}(p)\;a_{2}^{\prime H}(p)\;\cdots \;a_{N}^{\prime H}(p)\right]}^{H}\;,\\ \frac{\partial a^{\prime }(p)}{\partial p^{T}}={\left[{\left(\frac{\partial a_{1}^{\prime }(p)}{\partial p^{T}}\right)}^{H}\;{\left(\frac{\partial a_{2}^{\prime }(p)}{\partial p^{T}}\right)}^{H}\;\cdots \;{\left(\frac{\partial a_{N}^{\prime }(p)}{\partial p^{T}}\right)}^{H}\right]}^{H}\;,\\ a_{n}^{\prime }(p)={\left[a_{n,1}^{\prime H}(p)\;a_{n,2}^{\prime H}(p)\;\cdots \;a_{n,K}^{\prime H}(p)\right]}^{H}=A_{n}(p)1_{K\times 1}\;(1\leq n\leq N)\;. \end{array}\right.$$

Note that only the p corner of the CRB matrix is of interest here. However, it is easily observed from (78) that matrix $\mathrm{CRB}(\mu_{a})$ does not exhibit a block-diagonal structure, because there might be correlation between the parameters. Hence, it is somewhat difficult to obtain the CRB for position vector p. To overcome this difficulty, we adopt the idea of [59,67] to redefine a parameter vector whose CRB matrix becomes block-diagonal. The new parameter vector is defined as

(82) μ¯a=[pT  βrT  (Re{s¯}+Re{W1}⋅p+Re{W2}⋅βr)T  (Im{s¯}+Im{W1}⋅p+Im{W2}⋅βr)T]T ,

where

(83) {W1=ΩRe{s¯}†Ωp ,W2=ΩRe{s¯}†⋅[ΩRe{β}  ΩIm{β}] .

It is worth highlighting that because the vector ${\bar{\mu }}_{a}$ includes the source location parameters, it is meaningful to derive the CRB matrix for ${\bar{\mu }}_{a}$. In addition, there is a one-to-one mapping between the new and old vectors $\bar{\mu }$ and ${\bar{\mu }}_{a}$. The relationship between them can be written in matrix form as

(84) μ¯a=Jμa=[IOOOOIOORe{W1}Re{W2}IOIm{W1}Im{W2}OI]⋅μa ,

where

(85) J=[IOOOOIOORe{W1}Re{W2}IOIm{W1}Im{W2}OI] .

Then, combining the results in Proposition 3 and (84), the CRB matrix for ${\bar{\mu }}_{a}$ is given by

(86) CRB(μ¯a)=J⋅CRB(μa)⋅JT=σε22⋅(Re{(ΩμaJ−1)H(ΩμaJ−1)})−1 ,

where

(87) J−1=[IOOOOIOO−Re{W1}−Re{W2}IO−Im{W1}−Im{W2}OI] .

Combining (79), (83), and (87) leads to the orthogonal projection matrix

(88) ΩμaJ−1=[Τ⊥[ΩRe{s¯}]⋅ΩpΤ⊥[ΩRe{s¯}]⋅[ΩRe{β}  ΩIm{β}]ΩRe{s¯}j⋅ΩRe{s¯}] ,

where

(89) Τ⊥[ΩRe{s¯}]=I−ΩRe{s¯}ΩRe{s¯}†=I−ΩRe{s¯}(ΩRe{s¯}HΩRe{s¯})−1ΩRe{s¯}H

Inserting (88) back into (86) gives

(90) $$\mathrm{CRB}({\bar{\mu }}_{a})=\frac{\sigma_{\varepsilon }^{2}}{2}\cdot {\left[\begin{array}{cc} V_{1} & O\\ O & V_{2} \end{array}\right]}^{-1}\;,$$

where

(91) {V1=[Re{ΩpH⋅Τ⊥[ΩRe{s¯}]⋅Ωp}Re{ΩpH⋅Τ⊥[ΩRe{s¯}]⋅[ΩRe{β}  ΩIm{β}]}Re{[ΩRe{β}  ΩIm{β}]H⋅Τ⊥[ΩRe{s¯}]⋅Ωp}Re{[ΩRe{β}  ΩIm{β}]H⋅Τ⊥[ΩRe{s¯}]⋅[ΩRe{β}  ΩIm{β}]}] ,V2=[Re{ΩRe{s¯}HΩRe{s¯}}−Im{ΩRe{s¯}HΩRe{s¯}}Im{ΩRe{s¯}HΩRe{s¯}}Re{ΩRe{s¯}HΩRe{s¯}}] .

We define three matrices

(92) {V1,1=ΩpHΩp−ΩpHΩRe{s¯}(ΩRe{s¯}HΩRe{s¯})−1ΩRe{s¯}HΩp ,V1,2=[1  j]⊗(ΩpHΩRe{β}−ΩpHΩRe{s¯}(ΩRe{s¯}HΩRe{s¯})−1ΩRe{s¯}HΩRe{β}) ,V1,3=[1j−j1]⊗(ΩRe{β}HΩRe{β}−ΩRe{β}HΩRe{s¯}(ΩRe{s¯}HΩRe{s¯})−1ΩRe{s¯}HΩRe{β}) .

The details of calculating the matrices in (92) are provided in Appendix G. Invoking the partitioned matrix inversion formula, the CRB matrix for position vector p is given by

(93) CRB(p)=σε22⋅((Re{V1,1})−1+(Re{V1,1})−1⋅Re{V1,2}⋅(Re{V1,3}−Re{V1,2H}⋅(Re{V1,1})−1⋅Re{V1,2})−1⋅Re{V1,2H}⋅(Re{V1,1})−1) .

**Remark** **12.** *The diagonal elements of* CRB(p) *give the bounds for the estimation variance of the components in* p *when the array manifold is perfectly calibrated.*

**Remark** **13.** *The trace of* CRB(p) *is the bound for the localization MSE in the absence of array model errors.*

**Remark** **14.** *Although there is no rigorous proof, it is expected that the trace of* $P_{1}$ *is asymptotically close to that of* CRB(p)*. The reason for this is that the least square estimator in (5) is equivalent to the MLE, which is statistically efficient under the Gaussian noise model.*

**Remark** **15.** *By comparing the trace of* CRB(p) *with that of* P*, we can assess the expected degradation of the emitter location accuracy with respect to the amount of array model error. If the difference is significant, it can be concluded that the DPD method in* [29] *is sensitive to array model errors.*

### 8.2. Cramér-Rao Bound on Position Estimate in Presence of Array Model Errors

This goal of this subsection is to derive the CRB for the position estimate in the presence of array uncertainties. Because in the present case the full parameter set contains both the deterministic parameters p, β, $\bar{s}$, and $\sigma_{\varepsilon }^{2}$ and the stochastic parameter $\tilde{\varphi }$, the CRB derivation should follow the Bayesian theory frame [68,69,70]. It is noteworthy that the CRB derivation can also be used for stochastic parameters, as processed in [68,69,70]. To this end, a novel parameter vector that comprises all the deterministic and stochastic unknowns is introduced

(94) ηb=[σε2  pT  (Re{β})T  (Im{β})T  (Re{s¯})T  (Im{s¯})T  (Re{φ˜})T  (Im{φ˜})T]T=[σε2  μbT]T ,

where

(95) μb=[pT  (Re{β})T  (Im{β})T  (Re{s¯})T  (Im{s¯})T  (Re{φ˜})T  (Im{φ˜})T]T .

By performing similar algebraic manipulation in [68,69], the CRB matrix for vector $\mu_{b}$ is formulated as

(96) CRB(μb)=(2σε2⋅Re{ΩμbHΩμb}+[OOOΦ−1])−1 ,

where

(97) Ωμb=∂x¯0∂μbT=[Ωp  ΩRe{β}  ΩIm{β}  ΩRe{s¯}  ΩIm{s¯}  ΩRe{φ˜}  ΩIm{φ˜}] ,

(98) Φ=E[[Re{φ˜}Im{φ˜}]⋅[Re{φ˜}Im{φ˜}]T]=12⋅[blkdiag[Re{Φ1(1)+Φ1(2)} Re{Φ2(1)+Φ2(2)} ⋯ Re{ΦN(1)+ΦN(2)}]blkdiag[Im{Φ1(1)−Φ1(2)} Im{Φ2(1)−Φ2(2)} ⋯ Im{ΦN(1)−ΦN(2)}]blkdiag[Im{Φ1(1)+Φ1(2)} Im{Φ2(1)+Φ2(2)} ⋯ Im{ΦN(1)+ΦN(2)}]blkdiag[Re{Φ1(2)−Φ1(1)} Re{Φ2(2)−Φ2(1)} ⋯ Re{ΦN(2)−ΦN(1)}]] .

Note that (98) comes from the statistical assumption in (14). Appendix H provides the proof of (96).

Owing to the second term in the bracket of (96), it is impossible to get a CRB matrix with block diagonality as in (90) by linear transformation. As a result, the CRB matrix for position estimation can only be obtained from (96), although it may be computationally complex. Meanwhile, because the expressions for matrices $\Omega_{p}$, ΩRe{β}, ΩIm{β}, ΩRe{s¯}, and ΩIm{s¯} are given in (80), here we only need to deduce the expressions for matrices ΩRe{φ˜} and ΩIm{φ˜}. Applying (16) and (77) and performing algebraic manipulations gives

(99) {ΩRe{φ˜}=∂x¯0∂(Re{φ˜})T=diag[β⊗1MK×1]⋅blkdiag[s¯′1⊗IM  s¯′2⊗IM  ⋯  s¯′N⊗IM] ,ΩIm{φ˜}=∂x¯0∂(Im{φ˜})T=j⋅∂x¯0∂(Re{φ˜})T=j⋅diag[β⊗1MK×1]⋅blkdiag[s¯′1⊗IM  s¯′2⊗IM  ⋯  s¯′N⊗IM] .

Substituting (97) and (98) into (96) leads to

(100) CRB(μb)=(2σε2⋅Re{ΩpHΩpΩpHΩRe{β}ΩpHΩIm{β}ΩpHΩRe{s¯}ΩpHΩIm{s¯}ΩpHΩRe{φ˜}ΩpHΩIm{φ˜}ΩRe{β}HΩpΩRe{β}HΩRe{β}ΩRe{β}HΩIm{β}ΩRe{β}HΩRe{s¯}ΩRe{β}HΩIm{s¯}ΩRe{β}HΩRe{φ˜}ΩRe{β}HΩIm{φ˜}ΩIm{β}HΩpΩIm{β}HΩRe{β}ΩIm{β}HΩIm{β}ΩIm{β}HΩRe{s¯}ΩIm{β}HΩIm{s¯}ΩIm{β}HΩRe{φ˜}ΩIm{β}HΩIm{φ˜}ΩRe{s¯}HΩpΩRe{s¯}HΩRe{β}ΩRe{s¯}HΩIm{β}ΩRe{s¯}HΩRe{s¯}ΩRe{s¯}HΩIm{s¯}ΩRe{s¯}HΩRe{φ˜}ΩRe{s¯}HΩIm{φ˜}ΩIm{s¯}HΩpΩIm{s¯}HΩRe{β}ΩIm{s¯}HΩIm{β}ΩIm{s¯}HΩRe{s¯}ΩIm{s¯}HΩIm{s¯}ΩIm{s¯}HΩRe{φ˜}ΩIm{s¯}HΩIm{φ˜}ΩRe{φ˜}HΩpΩRe{φ˜}HΩRe{β}ΩRe{φ˜}HΩIm{β}ΩRe{φ˜}HΩRe{s¯}ΩRe{φ˜}HΩIm{s¯}ΩRe{φ˜}HΩRe{φ˜}ΩRe{φ˜}HΩIm{φ˜}ΩIm{φ˜}HΩpΩIm{φ˜}HΩRe{β}ΩIm{φ˜}HΩIm{β}ΩIm{φ˜}HΩRe{s¯}ΩIm{φ˜}HΩIm{s¯}ΩIm{φ˜}HΩRe{φ˜}ΩIm{φ˜}HΩIm{φ˜}}+[OOOΦ−1])−1=(2σε2⋅Re{Z1Z2Z2HZ3}+[OOOΦ−1])−1 ,

where

(101) {Z1=ΩpHΩp ,Z2=[[1  j]⊗(ΩpHΩRe{β})[1  j]⊗(ΩpHΩRe{s¯})[1  j]⊗(ΩpHΩRe{φ˜})] ,Z3=[[1j−j1]⊗(ΩRe{β}HΩRe{β})[1j−j1]⊗(ΩRe{β}HΩRe{s¯})[1j−j1]⊗(ΩRe{β}HΩRe{φ˜})[1j−j1]⊗(ΩRe{s¯}HΩRe{β})[1j−j1]⊗(ΩRe{s¯}HΩRe{s¯})[1j−j1]⊗(ΩRe{s¯}HΩRe{φ˜})[1j−j1]⊗(ΩRe{φ˜}HΩRe{β})[1j−j1]⊗(ΩRe{φ˜}HΩRe{s¯})[1j−j1]⊗(ΩRe{φ˜}HΩRe{φ˜})] .

The details of calculating the matrices in (101) appear in Appendix I. Through the application of the partitioned matrix inversion formula, the CRB matrix for position vector p is given by

(102) CRBe(p)=σε22⋅((Re{Z1})−1+(Re{Z1})−1⋅Re{Z2}⋅(Re{Z3}−Re{Z2H}⋅(Re{Z1})−1⋅Re{Z2}+[OOOσε2Φ−1/2])−1×Re{Z2H}⋅(Re{Z1})−1) .

Note that the subscript “e” in (102) is used to distinguish the matrix CRB(p) for the case where the knowledge of the array manifold is accurate.

**Remark** **16.** *The trace of* ${\mathrm{CRB}}_{e}(p)$ *is the bound for the localization MSE when array model errors exist.*

**Remark** **17.** *It is apparent that the trace of* ${\mathrm{CRB}}_{e}(p)$ *is larger than that of* CRB(p) *as the array model errors increase the uncertainties in parameter estimation.*

**Remark** **18.** *It can be readily proved that* $\mathrm{CRB}(p)={\mathrm{CRB}}_{e}(p)$ *when* $\Phi_{n}^{\left(1\right)}\to O$ *and* $\Phi_{n}^{\left(2\right)}\to O$*. Therefore, the CRB results derived in the presence of array model errors contain those for the case of no array model errors.*

**Remark** **19.** *Although there is no strict proof, it is not hard to conclude that the trace of* P *is greater than that of* ${\mathrm{CRB}}_{e}(p)$*. The reason is that the DPD estimator discussed here does not take the array model errors into account and, thus, it is not statistically efficient for this case. Hence, a comparison of the trace of* ${\mathrm{CRB}}_{e}(p)$ *with that of* P *allows us to decide whether a new DPD method that accounts for the array model errors is necessary to improve the emitter location accuracy.*

## 9. Simulation Results

This section presents a set of Monte Carlo simulations to support the theoretical development in the previous sections. The empirical performances of the DPD method with and without array model errors are given, and they are compared both to the theoretical prediction values given in Section 6 and Section 7 and to the CRBs presented in Section 8. The simulated values are averaged over 5000 independent trials. Moreover, the root-mean-square-error (RMSE), SP of localization, and radius of CEP are used to assess and compare the performance.

### 9.1. Discussion on RMSE of Direct Localization

This subsection focuses on the RMSE of the DPD method. Two sets of experiments are reported to illustrate the usefulness of the obtained results.

#### 9.1.1. The First Set of Experiments

In the first set of experiments, the location estimation is performed on a 2-D plane and a simple array error model is used, which corresponds to case 1 in Section 4 in [44]. Specifically, ${\tilde{\varphi }}_{n}$ follows a circularly symmetric complex Gaussian distribution with second-order moments given by

(103) $$E[{\tilde{\varphi }}_{n}{\tilde{\varphi }}_{n}^{T}]=\Phi_{n}^{\left(1\right)}=O_{M\times M}\;,\;E[{\tilde{\varphi }}_{n}{\tilde{\varphi }}_{n}^{H}]=\Phi_{n}^{\left(2\right)}=\sigma_{\tilde{\varphi }}^{2}I_{M}\;(1\leq n\leq N)\;,$$

where $\sigma_{\tilde{\varphi }}$ is the standard deviation of the array model error.

**Figure 1:**
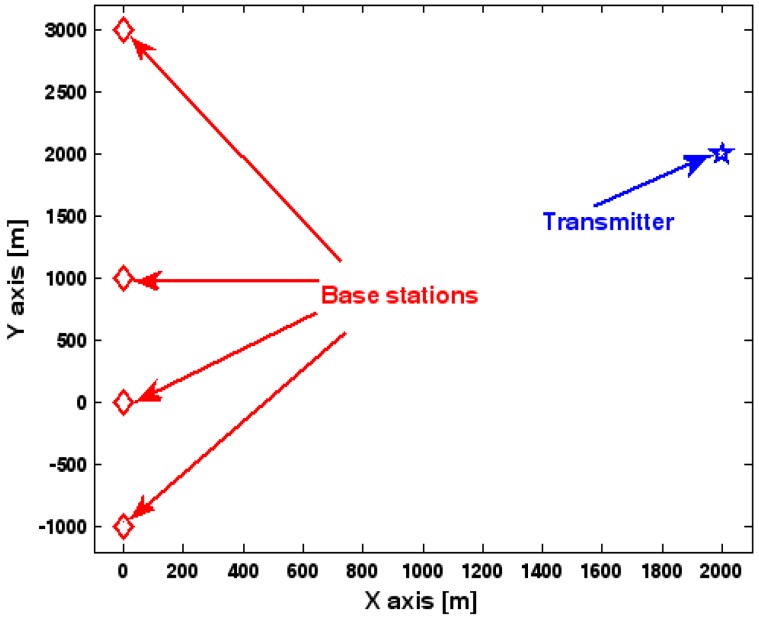
Location geometry for simulation.

**Figure 2:**
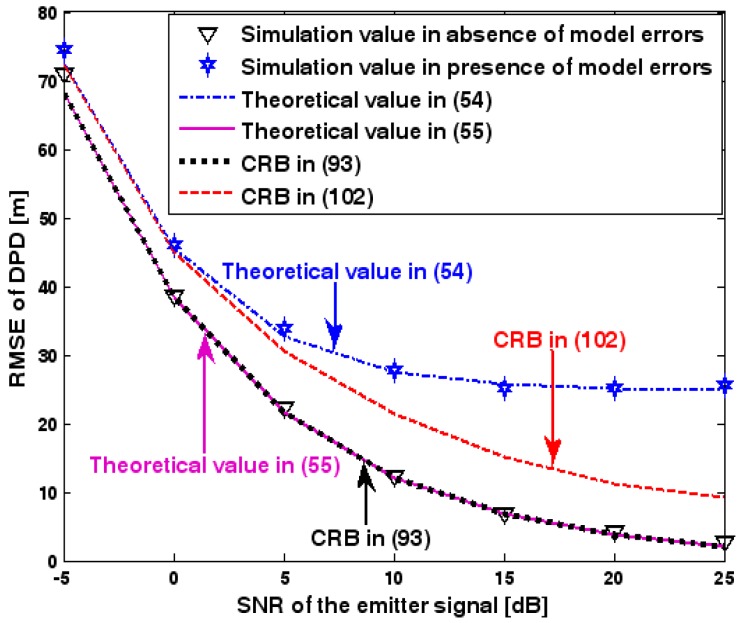
Root-mean-square-error (RMSE) of direct position determination (DPD) versus signal-to-noise ratio (SNR) of the emitter signal.

**Figure 3:**
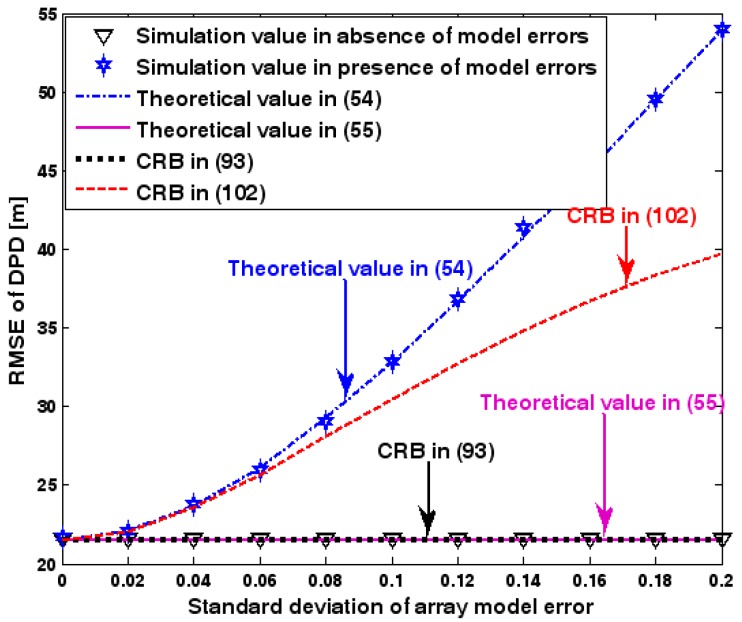
RMSE of DPD versus standard deviation of array model error.

**Figure 4:**
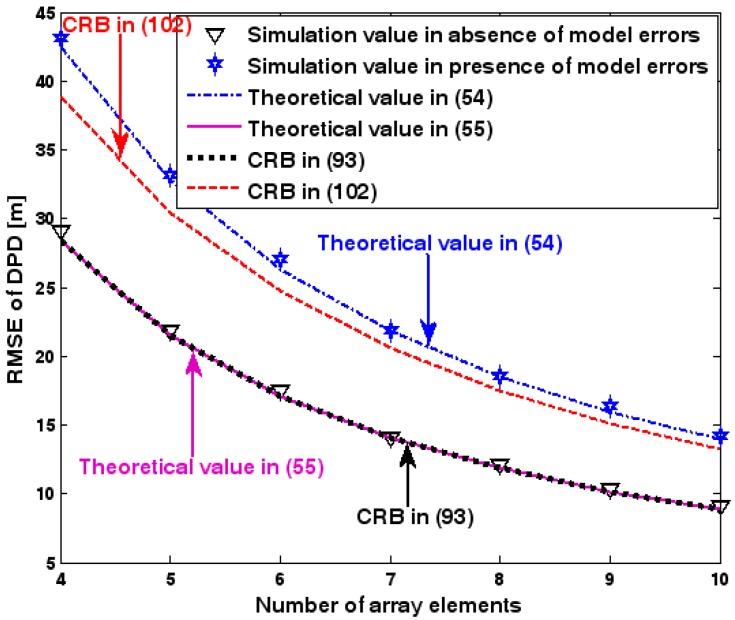
RMSE of DPD versus number of array elements.

**Figure 5:**
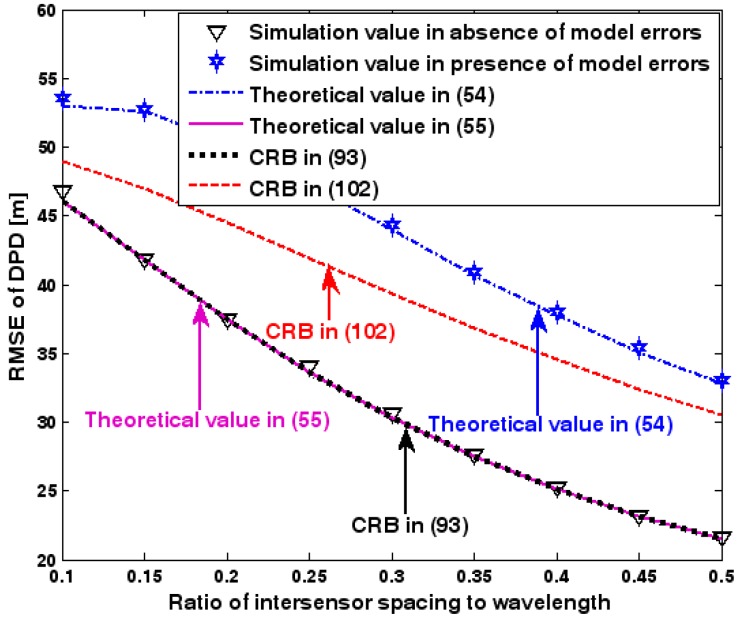
RMSE of DPD versus ratio of intersensor spacing to wavelength.

**Figure 6:**
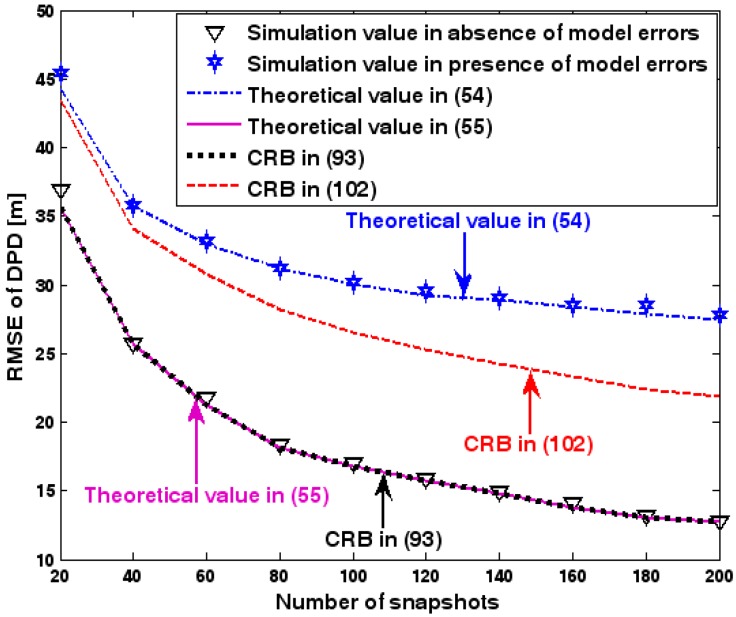
RMSE of DPD versus number of snapshots.

The location geometry of the first set of experiments is shown in Figure 1, where both base stations and transmitter lie on a plane. We consider four base stations with coordinates [0, 1000] m, [0,0] m, [0,1000] m, and [0, 3000] m, while the emitter position is fixed at [2000, 2000] m. The transmitted waveforms are realizations of a normal Gaussian random process, and are unknown to the receivers. Each base station is equipped with a uniform linear array. The channel attenuation magnitude is fixed at 1, and the channel phase is selected at random from a uniform distribution over [−π, π). In addition, unless stated otherwise, we use the settings (1) K=64 samples; (2) SNR of 5 dB; (3) M=5 sensors; (4) $\sigma_{\tilde{\varphi }}=0.1$; and (5) sensor elements are separated by a half wavelength. Figure 2, Figure 3, Figure 4, Figure 5 and Figure 6 display the RMSEs of the DPD method, as functions of the SNR of the emitter signal, the standard deviation of array model error $\sigma_{\tilde{\varphi }}$, the number of array elements M, the ratio of the intersensor spacing to wavelength, and the number of snapshots K.

Figure 2, Figure 3, Figure 4, Figure 5 and Figure 6 reveal that the theoretical RMSE provided by (54) is in close agreement with the simulation result in the presence of array model errors. Consequently, the validity of the theoretical study in Section 6 is confirmed. Furthermore, when array model errors are absent, the empirical RMSE is very close to the CRB given by (92) and the theoretical RMSE in (55), which implies the asymptotical efficiency of the DPD method presented in [29], provided that the array is accurately calibrated. It is also seen that, as expected, the presence of array model errors leads to considerable deteriorations in location accuracy. Furthermore, Figure 2 and Figure 6 show that the RMSE of the DPD method remains approximately constant no matter how much the SNR and sample number increase. The reason for this is that when the SNR or the sample number is large enough, the effects of sensor noise can be neglected and the localization errors are therefore primarily caused by array model errors, whose affects cannot be effectively eliminated in this DPD method yet. Additionally, we find that the RMSE performance in the presence of array uncertainties is significantly greater than the CRB provided by (102), especially when the standard deviation $\sigma_{\tilde{\varphi }}$ increases (see Figure 3). Consequently, a new DPD method that accounts for array model errors is needed to improve the location accuracy.

#### 9.1.2. The Second Set of Experiments

In the second set of experiments, the source location is estimated in 3-D space and we assume that the array error is caused by sensor gain and phase uncertainties, which corresponds to case 2 in Section 4 in [44]. The second-order moments of ${\tilde{\varphi }}_{n}$ can then be expressed as

(104) $$\left\{ \begin{array}{l} E[{\tilde{\varphi }}_{n}{\tilde{\varphi }}_{n}^{T}]=\Phi_{n}^{\left(1\right)}=(\sigma_{{\tilde{\varphi }}_{1}}^{2}-\sigma_{{\tilde{\varphi }}_{2}}^{2})\cdot \mathrm{diag}[a_{n}(p)◉a_{n}(p)]\;,\\ E[{\tilde{\varphi }}_{n}{\tilde{\varphi }}_{n}^{H}]=\Phi_{n}^{\left(2\right)}=(\sigma_{{\tilde{\varphi }}_{1}}^{2}+\sigma_{{\tilde{\varphi }}_{2}}^{2})\cdot \mathrm{diag}[a_{n}(p)◉a_{n}^{*}(p)]\;, \end{array}\right.\;(1\leq n\leq N)\;,$$

where $\sigma_{{\tilde{\varphi }}_{1}}$ and $\sigma_{{\tilde{\varphi }}_{2}}$ are the sensor gain and phase perturbation standard deviation, respectively. Moreover, we assume $\sigma_{{\tilde{\varphi }}_{1}}=2\sigma_{{\tilde{\varphi }}_{2}}$ hereafter, and thus if $\sigma_{{\tilde{\varphi }}_{1}}$ is changed, $\sigma_{{\tilde{\varphi }}_{2}}$ alters accordingly.

**Figure 7:**
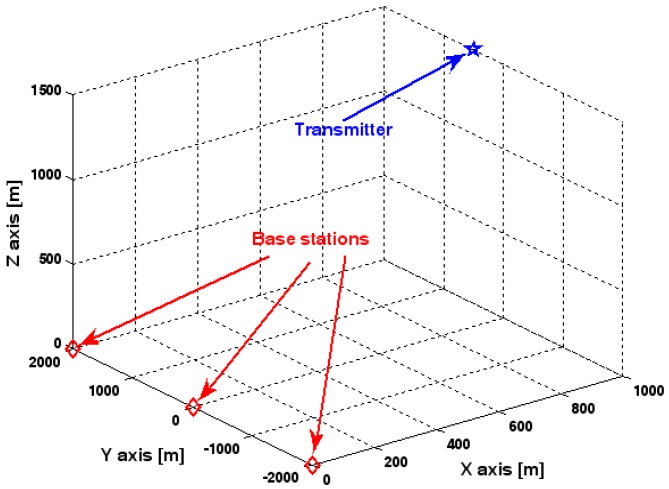
Source location scenario for simulation.

**Figure 8:**
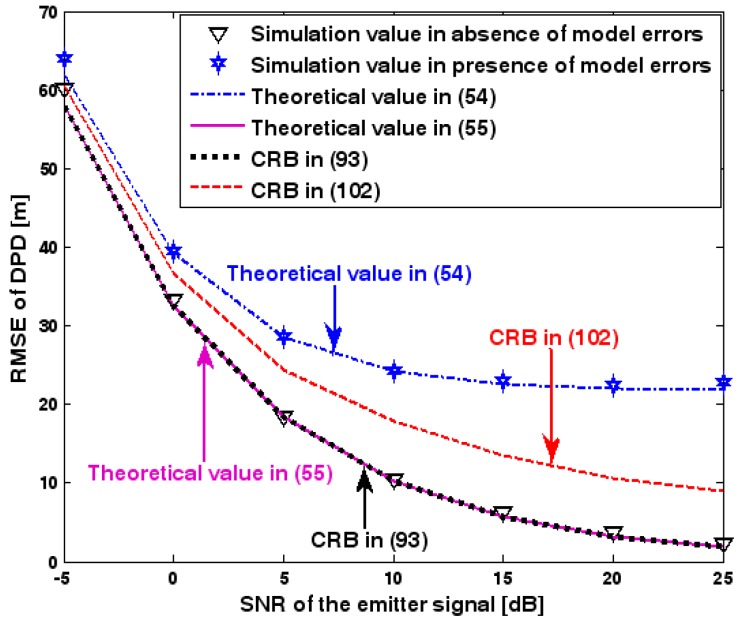
RMSE of DPD as a function of SNR of the emitter signal.

**Figure 9:**
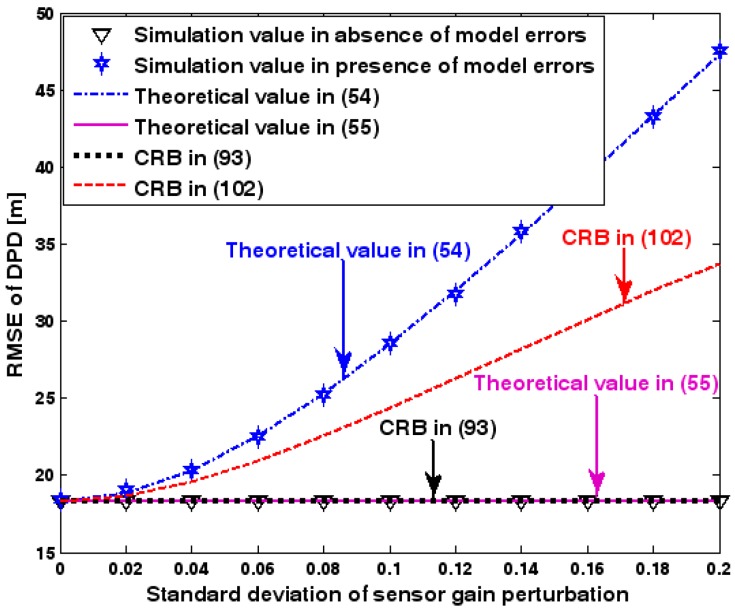
RMSE of DPD as a function of standard deviation of sensor gain perturbation.

**Figure 10:**
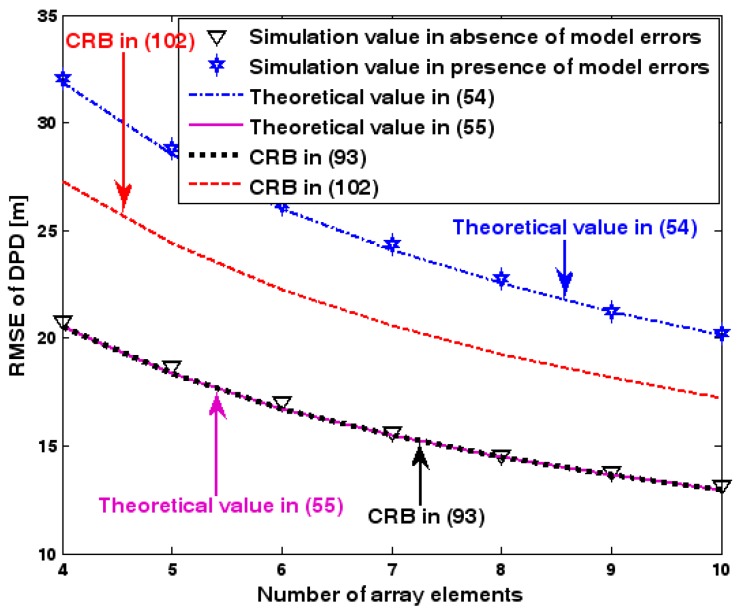
RMSE of DPD as a function of number of array elements.

**Figure 11:**
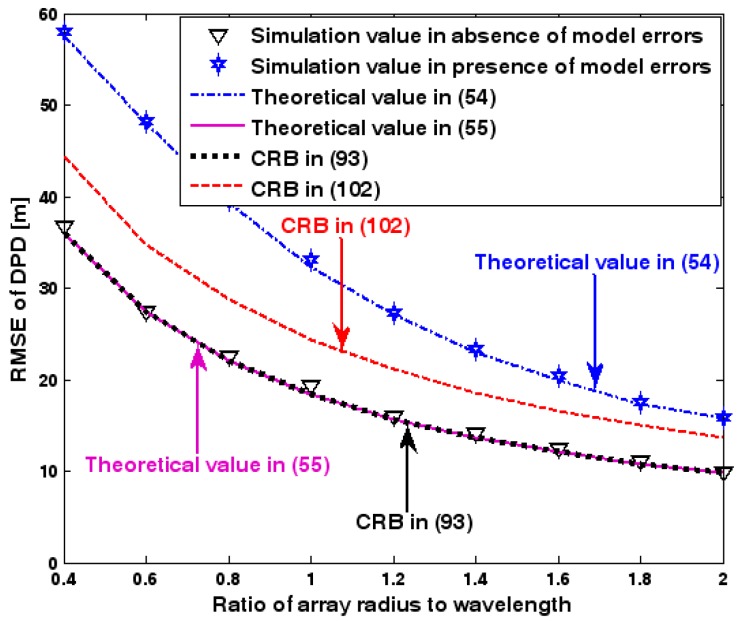
RMSE of DPD as a function of ratio of array radius to wavelength.

**Figure 12:**
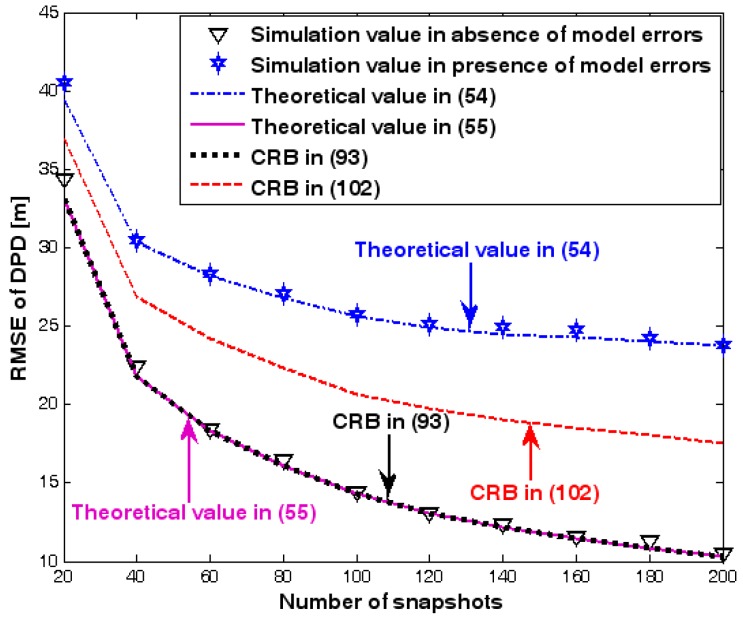
RMSE of DPD as a function of number of snapshots.

Figure 7 illustrates the geometry for the source location in the second set of experiments. Obviously, it depicts a 3-D localization scenario. The source is positioned at [1000, 500, 1500] m, and the coordinates of three base stations are set to [0, 2000, 0] m, [0, 0, 0] m, and [0, −2000, 0] m. Each base station is equipped with a uniform circular array. The envelope of the transmitted signal and array model errors are generated in exactly the same manner as previously. Additionally, unless stated otherwise, we adopt the settings (1) K=64 samples; (2) SNR of 5 dB; (3) M=5 sensors; (4) $\sigma_{{\tilde{\varphi }}_{1}}=0.1$; and (5) an array radius equal to the wavelength. Figure 8, Figure 9, Figure 10, Figure 11 and Figure 12 show the RMSEs of the DPD method by varying the SNR of the emitter signal, standard deviation of sensor gain perturbation $\sigma_{{\tilde{\varphi }}_{1}}$, the number of array elements M, the ratio of array radius to wavelength, and the number of snapshots K.

The results presented in Figure 8, Figure 9, Figure 10, Figure 11 and Figure 12 coincide with the results presented Figure 2, Figure 3, Figure 4, Figure 5 and Figure 6 although the dimensionality of the localization scenario and the model of the array error differ from each other. Owing to limited space, we do not present the results again. We simply highlight that the good agreement between the empirical and theoretical RMSE once again demonstrates the effectiveness of the theoretical development in Section 6.

### 9.2. Discussion on Success Probability of Direct Localization

This subsection focuses on the SP of the DPD method. Two sets of experiments are carried out to validate the obtained probability formulas, and the simulation parameters are the same as those in Section 9.1.

#### 9.2.1. The First Set of Experiments

**Figure 13:**
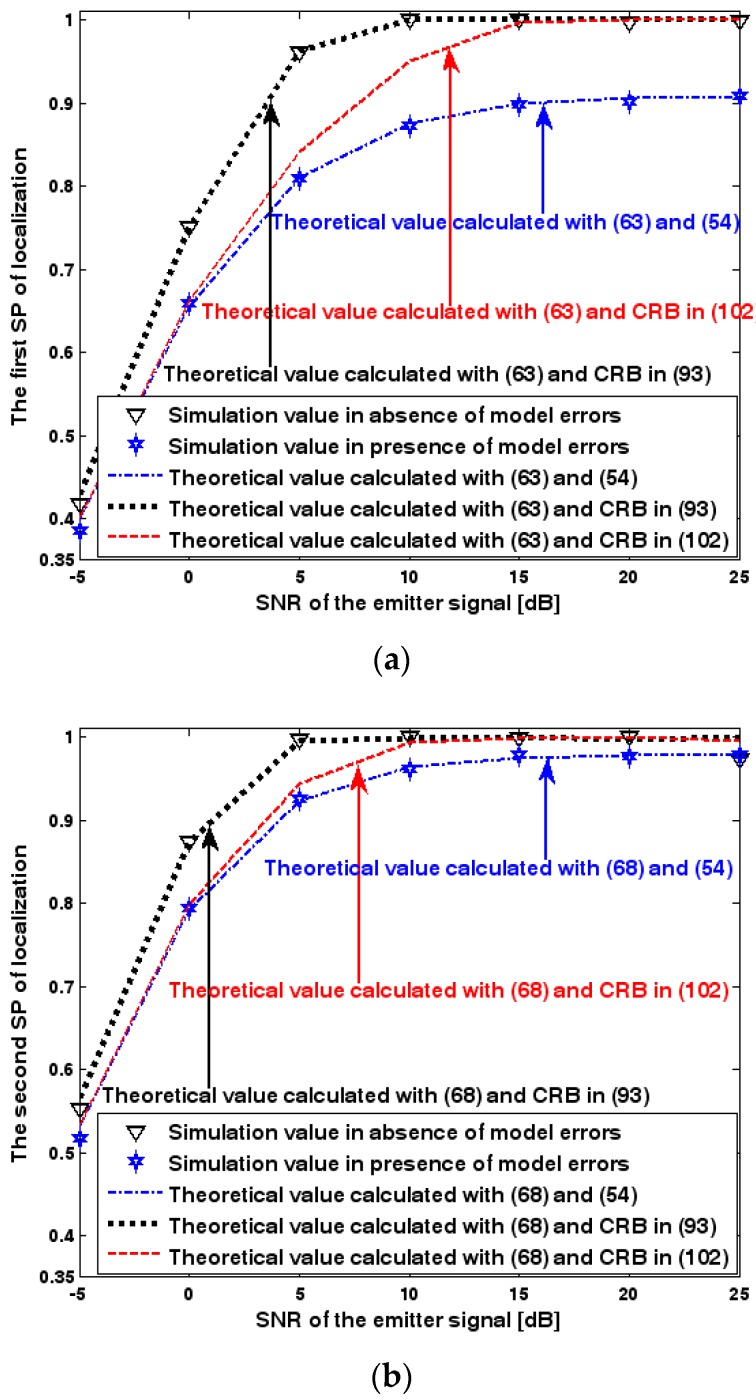
Success probability (SP) of localization versus SNR of the emitter signal. (**a**) The first SP of localization versus SNR of the emitter signal. (**b**) The second SP of localization versus SNR of the emitter signal.

**Figure 14:**
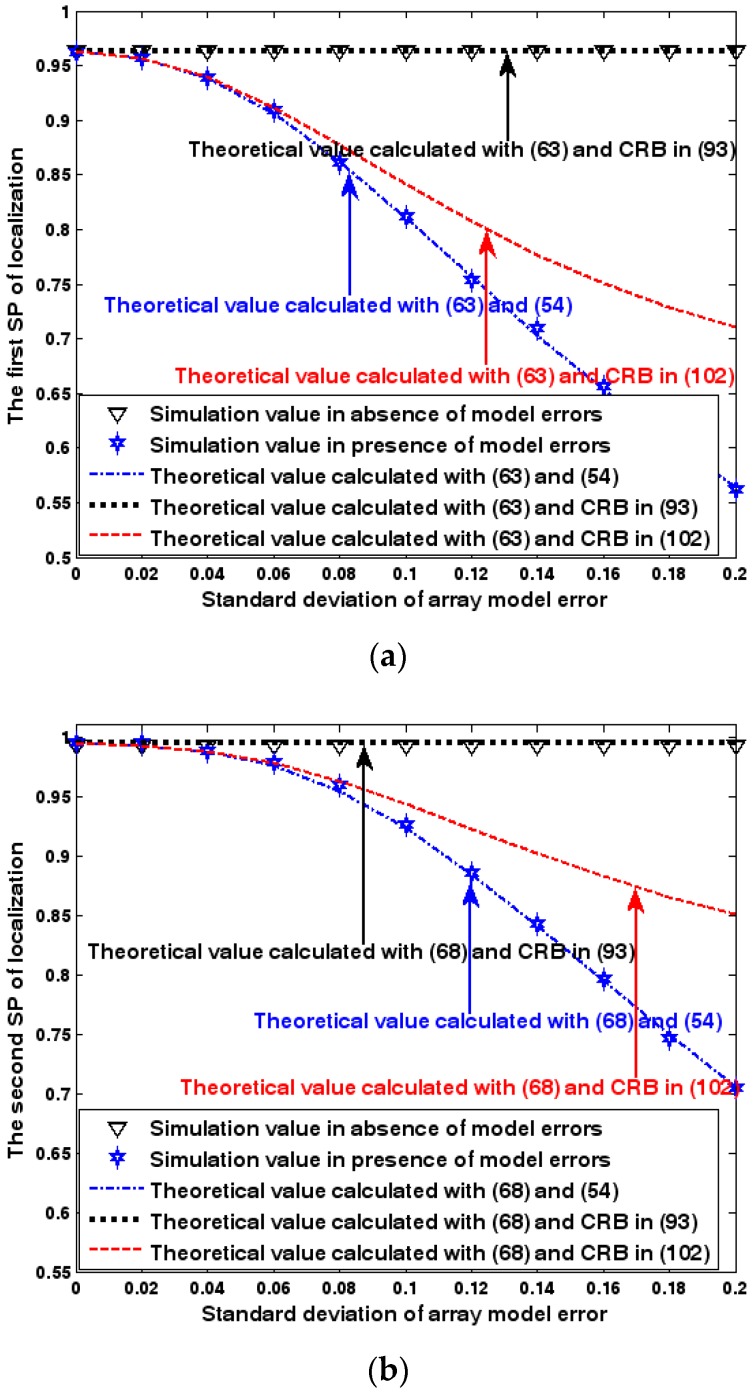
SP of localization versus standard deviation of array model error. (**a**) The first SP of localization versus standard deviation of array model error. (**b**) The second SP of localization versus standard deviation of array model error.

**Figure 15:**
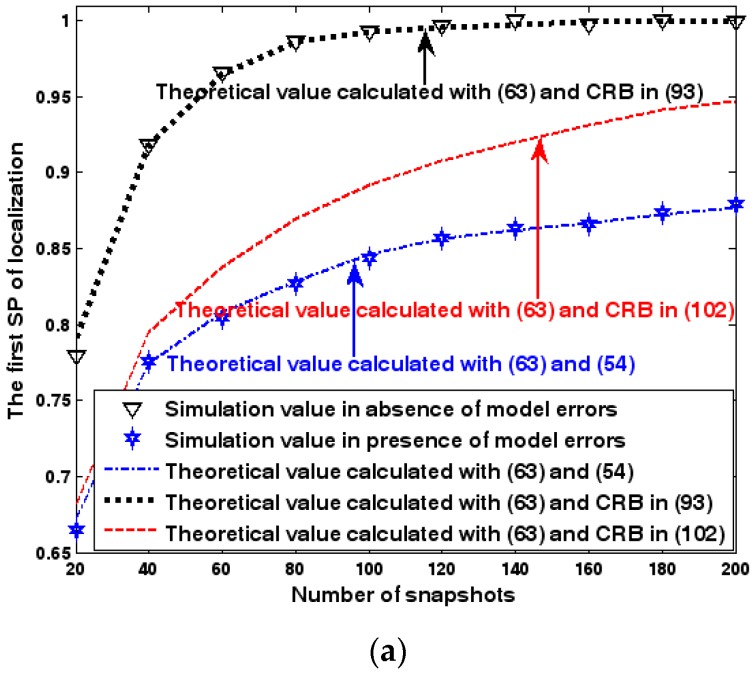

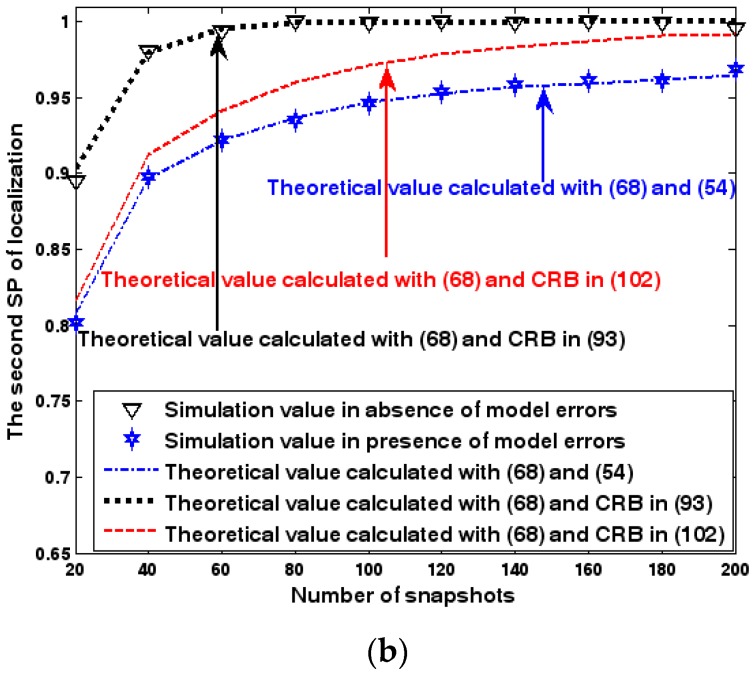
SP of localization versus number of snapshots. (**a**) The first SP of localization versus number of snapshots. (**b**) The second SP of localization versus number of snapshots.

Both the localization scenario and the array error model for the first set of experiments are the same as those in Section 9.1.1. Moreover, the parameters $\Delta_{1}$ and $\Delta_{2}$, which are used to specify the first SP, are set to the same value of 40, and the parameter Δ, which is related to the second SP, is also selected as 40. Because the localization scenario is on a 2-D plane, the theoretical value of the first SP can be obtained with (63). In Figure 13, Figure 14 and Figure 15, we plot the two kinds of SP of the DPD method against the SNR of the emitter signal, standard deviation of the array model error $\sigma_{\tilde{\varphi }}$, and number of snapshots K.

Figure 13, Figure 14 and Figure 15 reveal that there is a close match between the analytical results and the simulation results and hence the validity of (63) and (68) can be supported. Furthermore, the simulated values in the absence of array model errors approach the lower bound calculated with the CRB in (93), which further indicates that the DPD estimator can achieve the CRB accuracy as long as the array is perfectly calibrated. However, when array model errors exist, the empirical values deviate significantly from the lower bound. Moreover, the difference increases with the standard deviation of array model error (see Figure 14). We thus need to develop a new DPD estimator with improved robustness against array model errors. Furthermore, it is seen that the first SP is always smaller than the second SP, which is consistent with the analysis in Remark 11.

#### 9.2.2. The Second Set of Experiments

**Figure 16:**
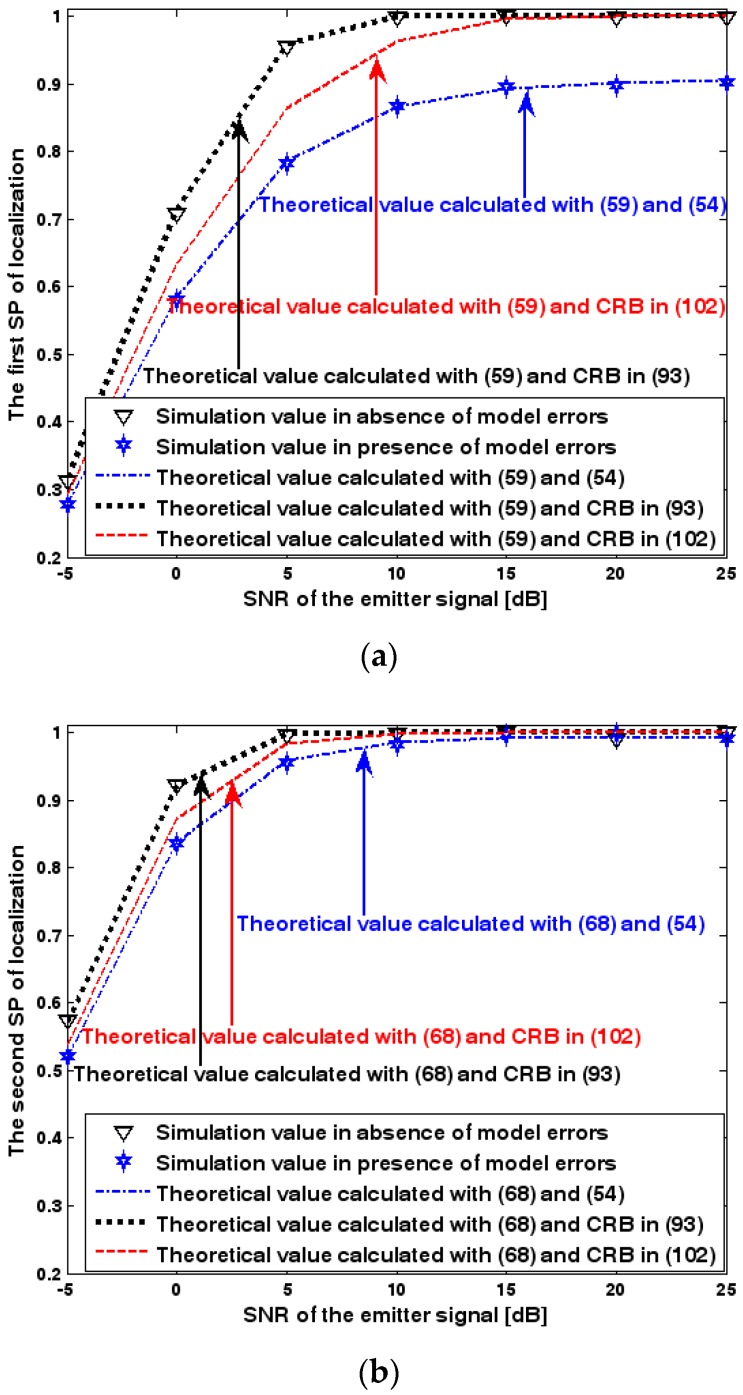
SP of localization as a function of SNR of the emitter signal. (**a**) The first SP of localization as a function of SNR of the emitter signal. (**b**) The second SP of localization as a function of SNR of the emitter signal.

**Figure 17:**
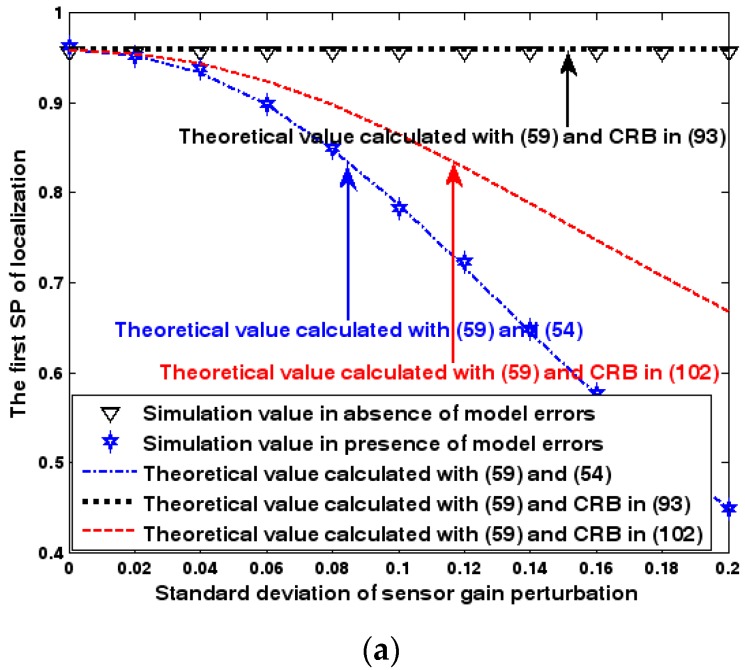

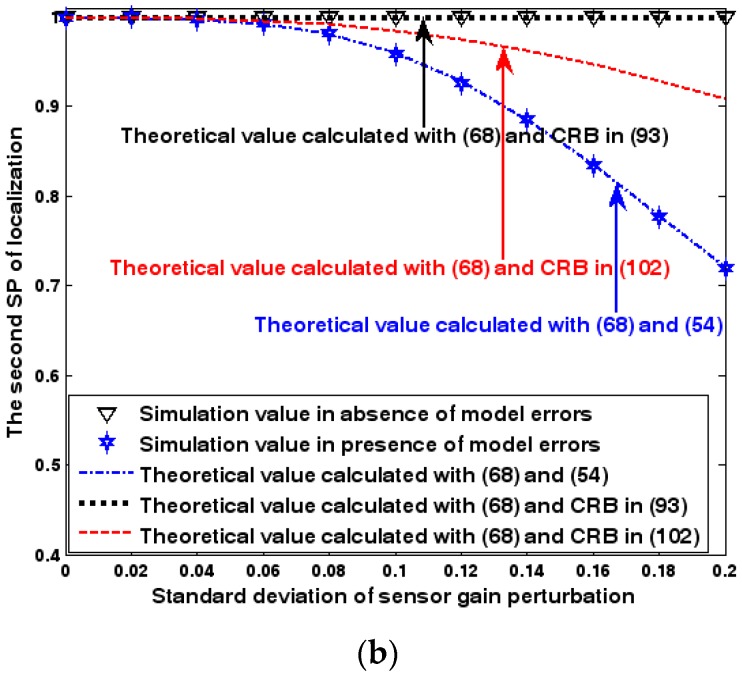
SP of localization as a function of standard deviation of sensor gain perturbation. (**a**) The first SP of localization as a function of standard deviation of sensor gain perturbation. (**b**) The second SP of localization as a function of standard deviation of sensor gain perturbation.

**Figure 18:**
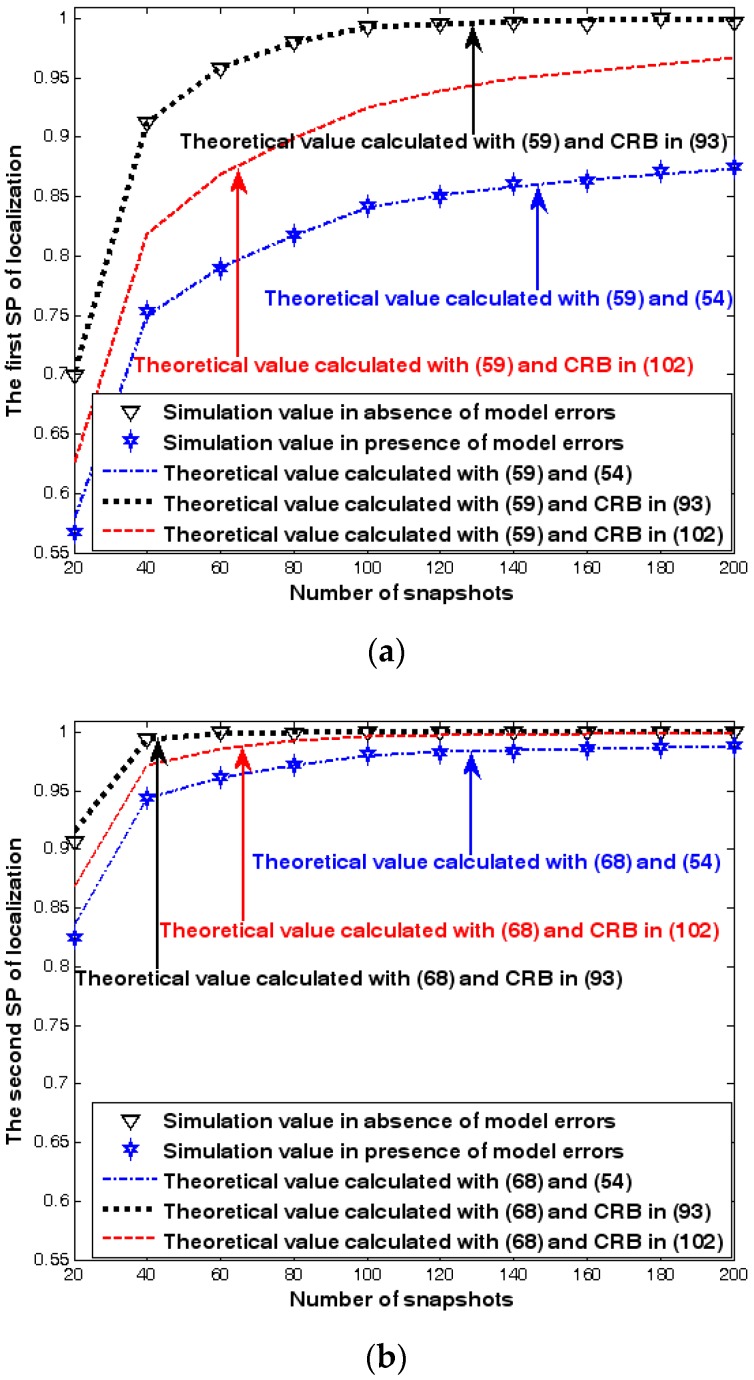
SP of localization as a function of number of snapshots. (**a**) The first SP of localization as a function of number of snapshots. (**b**) The second SP of localization as a function of number of snapshots.

Both the localization scenario and the array error model for the second set of experiments are the same as those in Section 9.1.2. Because the situation is a 3-D localization scenario, the theoretical value of the first SP must be calculated with numerical integration methods. Herein, the Richardson extrapolation algorithm is exploited. Figure 16, Figure 17 and Figure 18 depict the two kinds of SP of the DPD method as functions of the SNR of the emitter signal, standard deviation of sensor gain perturbation $\sigma_{{\tilde{\varphi }}_{1}}$, and number of snapshots K.

For Figure 16, Figure 17 and Figure 18 we make similar observations as for Figure 13, Figure 14 and Figure 15. We simply emphasize that the good agreement between empirical and analytical SP once again validates the probability formulas obtained in Section 7.

### 9.3. Discussion on Radius of CEP

This subsection discusses the radius of CEP of the DPD method. Two simulation experiments are conducted to illustrate the validity of (73), which is used to estimate the radius of CEP. The first and second simulation settings are the same as those in Figure 2 and Figure 8, respectively. In the following two figures, the radius of CEP of the DPD method in the two experiments is plotted as a function of the SNR of the emitter signal.

**Figure 19:**
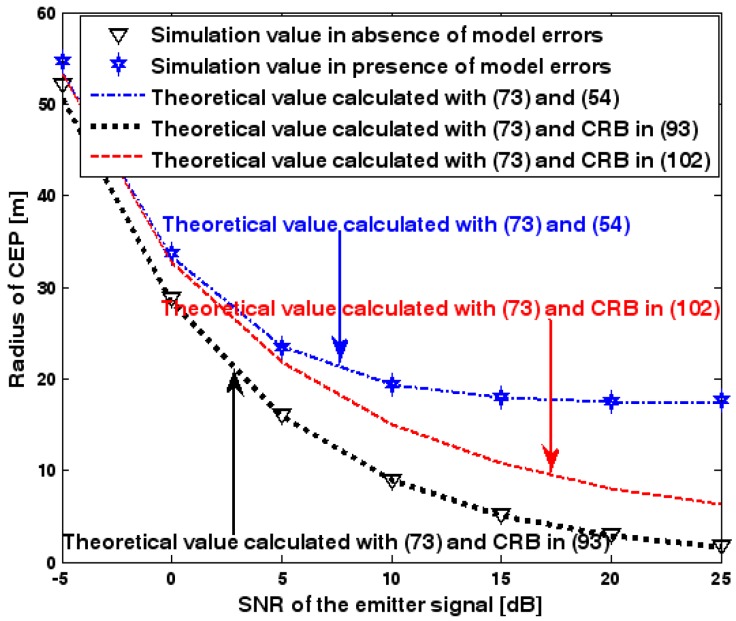
Radius of CEP versus SNR of the emitter signal in the first experiment.

**Figure 20:**
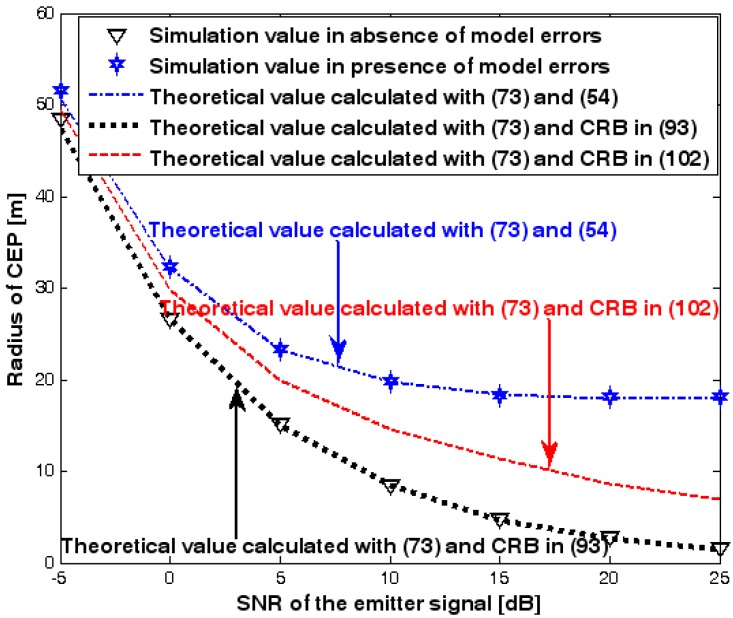
Radius of circular error probable (CEP) versus SNR of the emitter signal in the second experiment.

Figure 19 and Figure 20 show that the simulation results agree well with the analytical results calculated with (73) and therefore the validity of (73) is corroborated. Moreover, we observe that the increase in the radius of CEP due to the array model errors is significant, especially when the SNR of the emitter signal is sufficiently high. Furthermore, when array model errors exist, the radius of CEP remains approximately constant no matter how much the SNR increases. Therefore, a robust DPD method that restrains the uncertainties in an array manifold is required.

## 10. Conclusions

In this paper, the statistical performance of the DPD estimator presented in [29] is analytically studied when array model errors are present as well as signal waveforms are not available. The theoretical analysis begins with a matrix eigen-perturbation result, which can express the perturbation of eigenvalues as a function of the disturbance added to the Hermitian matrix. Then, the first-order asymptotic expression of the localization errors is given, from which the analytical expression for the MSE of the DPD estimator is obtained. Besides, the closed-form expressions for the calculation of the probabilities of a successful localization are also deduced, which can offer another theoretical perspective on the study of the localization accuracy. Additionally, the obtained probability formula can be used to provide a new criterion to estimate the radius of CEP. Finally, the CRB expressions for the position estimation are derived for two cases: (a) array model errors do not exist, and (b) array model errors are present and are drawn from Gaussian distribution. Several simulation experiments are performed to confirm the usefulness of the obtained results. The experimental results show that the uncertainties in the model of the array manifold can seriously deteriorate the source location accuracy of the DPD method. Therefore, our future work is to present a new DPD method that is expected to be more robust against the array model errors.

## Figures and Tables

**Table 1:** Notational conventions.

Notation	Explanation
⊗	Kronecker product
◉	Schur product
diag [⋅]	a diagonal matrix with diagonal entries formed from the vector
blkdiag [⋅]	a block-diagonal matrix formed from the matrices or vectors
${\left[\cdot \right]}^{\text{†}}$	Moore-Penrose inverse of the matrix
$I_{n}$	n×n identity matrix
$i_{n}^{\left(k\right)}$	the *k*th column vector of $I_{n}$
$O_{n\times m}$	n×m matrix of zeros
$1_{n\times 1}$	n×1 vector of ones
$\lambda_{\max}\{\cdot \}$	the largest eigenvalue of the matrix
$||\cdot |{|}_{2}$	Euclidean norm
$<\cdot {>}_{n}$	the *n*th entry of the vector
$<\cdot {>}_{nm}$	the *nm*th entry of the matrix
Re{⋅}	real part of the argument
Im{⋅}	imaginary part of the argument
Pr{⋅}	probability of the given event
E[⋅]	mathematical expectation of the random variable
var[⋅]	variance of the random variable

## Appendix A—Detailed Derivation of Matrices in (30)

It follows from the first equality in (9) and the first equality in (12) that

(A1) $${\dot{A}}_{l}(p)=\frac{\partial A(p)}{\partial <p{>}_{l}}=\left[\begin{array}{c} \frac{\partial A_{1}(p)}{\partial <p{>}_{l}}\\ \frac{\partial A_{2}(p)}{\partial <p{>}_{l}}\\ \vdots \\ \frac{\partial A_{N}(p)}{\partial <p{>}_{l}} \end{array}\right]=\left[\begin{array}{c} \mathrm{blkdiag}\left[\frac{\partial a_{1,1}^{\prime }(p)}{\partial <p{>}_{l}}\;\frac{\partial a_{1,2}^{\prime }(p)}{\partial <p{>}_{l}}\;\cdots \;\frac{\partial a_{1,K}^{\prime }(p)}{\partial <p{>}_{l}}\right]\\ \mathrm{blkdiag}\left[\frac{\partial a_{2,1}^{\prime }(p)}{\partial <p{>}_{l}}\;\frac{\partial a_{2,2}^{\prime }(p)}{\partial <p{>}_{l}}\;\cdots \;\frac{\partial a_{2,K}^{\prime }(p)}{\partial <p{>}_{l}}\right]\\ \vdots \\ \mathrm{blkdiag}\left[\frac{\partial a_{N,1}^{\prime }(p)}{\partial <p{>}_{l}}\;\frac{\partial a_{N,2}^{\prime }(p)}{\partial <p{>}_{l}}\;\cdots \;\frac{\partial a_{N,K}^{\prime }(p)}{\partial <p{>}_{l}}\right] \end{array}\right]\;,$$

(A2) $${\ddot{A}}_{l_{1}l_{2}}(p)=\frac{\partial^{2}A(p)}{\partial <p{>}_{l_{1}}\partial <p{>}_{l_{2}}}=\left[\begin{array}{c} \frac{\partial^{2}A_{1}(p)}{\partial <p{>}_{l_{1}}\partial <p{>}_{l_{2}}}\\ \frac{\partial^{2}A_{2}(p)}{\partial <p{>}_{l_{1}}\partial <p{>}_{l_{2}}}\\ \vdots \\ \frac{\partial^{2}A_{N}(p)}{\partial <p{>}_{l_{1}}\partial <p{>}_{l_{2}}} \end{array}\right]=\left[\begin{array}{c} \mathrm{blkdiag}\left[\frac{\partial^{2}a_{1,1}^{\prime }(p)}{\partial <p{>}_{l_{1}}\partial <p{>}_{l_{2}}}\;\frac{\partial^{2}a_{1,2}^{\prime }(p)}{\partial <p{>}_{l_{1}}\partial <p{>}_{l_{2}}}\;\cdots \;\frac{\partial^{2}a_{1,K}^{\prime }(p)}{\partial <p{>}_{l_{1}}\partial <p{>}_{l_{2}}}\right]\\ \mathrm{blkdiag}\left[\frac{\partial^{2}a_{2,1}^{\prime }(p)}{\partial <p{>}_{l_{1}}\partial <p{>}_{l_{2}}}\;\frac{\partial^{2}a_{2,2}^{\prime }(p)}{\partial <p{>}_{l_{1}}\partial <p{>}_{l_{2}}}\;\cdots \;\frac{\partial^{2}a_{2,K}^{\prime }(p)}{\partial <p{>}_{l_{1}}\partial <p{>}_{l_{2}}}\right]\\ \vdots \\ \mathrm{blkdiag}\left[\frac{\partial^{2}a_{N,1}^{\prime }(p)}{\partial <p{>}_{l_{1}}\partial <p{>}_{l_{2}}}\;\frac{\partial^{2}a_{N,2}^{\prime }(p)}{\partial <p{>}_{l_{1}}\partial <p{>}_{l_{2}}}\;\cdots \;\frac{\partial^{2}a_{N,K}^{\prime }(p)}{\partial <p{>}_{l_{1}}\partial <p{>}_{l_{2}}}\right] \end{array}\right]\;,$$

where

(A3) $$\frac{\partial a_{n,k}^{\prime }(p)}{\partial <p{>}_{l}}=\exp\{-j\omega_{k}\tau_{n}(p)\}\cdot \left(\frac{\partial a_{n}(p)}{\partial <p{>}_{l}}-j\omega_{k}a_{n}(p)\cdot \frac{\partial \tau_{n}(p)}{\partial <p{>}_{l}}\right)\;,$$

(A4) $$\begin{array}{cc} \frac{\partial^{2}{a^{\prime }}_{n,k}(p)}{\partial <p{>}_{l_{1}}\partial <p{>}_{l_{2}}} & =\exp\{-j\omega_{k}\tau_{n}(p)\}\cdot \left(\frac{\partial^{2}a_{n}(p)}{\partial <p{>}_{l_{1}}\partial <p{>}_{l_{2}}}-j\omega_{k}\left(\frac{\partial a_{n}(p)}{\partial <p{>}_{l_{2}}}\cdot \frac{\partial \tau_{n}(p)}{\partial <p{>}_{l_{1}}}+a_{n}(p)\cdot \frac{\partial^{2}\tau_{n}(p)}{\partial <p{>}_{l_{1}}\partial <p{>}_{l_{2}}}\right)\right)\\ & -j\omega_{k}\cdot \exp\{-j\omega_{k}\tau_{n}(p)\}\cdot \frac{\partial \tau_{n}(p)}{\partial <p{>}_{l_{2}}}\cdot \left(\frac{\partial a_{n}(p)}{\partial <p{>}_{l_{1}}}-j\omega_{k}a_{n}(p)\cdot \frac{\partial \tau_{n}(p)}{\partial <p{>}_{l_{1}}}\right)\;. \end{array}$$

## Appendix B—Proof of (34) and (35)

Applying Proposition 1 and (31) leads to

(A5) $$\begin{array}{cc} \hat{J}(\hat{p})= & \lambda_{\max}\{{\hat{B}}^{H}(\hat{p})\hat{B}(\hat{p})\}=\lambda_{\max}\{C_{0}\}+u_{N}^{H}(C_{0}+{\tilde{C}}^{\left(1\right)}+{\tilde{C}}^{\left(2\right)}+o({\left||\tilde{\xi }|\right|}_{2}^{2}))u_{N}\\ & +u_{N}^{H}(C_{0}+{\tilde{C}}^{\left(1\right)}+{\tilde{C}}^{\left(2\right)}+o({\left||\tilde{\xi }|\right|}_{2}^{2}))U_{N}(C_{0}+{\tilde{C}}^{\left(1\right)}+{\tilde{C}}^{\left(2\right)}+o({\left||\tilde{\xi }|\right|}_{2}^{2}))u_{N}+o(||{\tilde{C}}^{\left(1\right)}+{\tilde{C}}^{\left(2\right)}+o({\left||\tilde{\xi }|\right|}_{2}^{2}){\left|\right|}_{2}^{2})\\ = & \lambda_{\max}\{C_{0}\}+u_{N}^{H}({\tilde{C}}^{\left(1\right)}+{\tilde{C}}^{\left(2\right)})u_{N}+u_{N}^{H}{\tilde{C}}^{\left(1\right)}U_{N}{\tilde{C}}^{\left(1\right)}u_{N}+o({\left||\tilde{\xi }|\right|}_{2}^{2})\\ = & \lambda_{N}+{\tilde{\lambda }}_{N}^{\left(1\right)}+{\tilde{\lambda }}_{N}^{\left(2\right)}+o({\left||\tilde{\xi }|\right|}_{2}^{2})\;, \end{array}$$

where ${\tilde{\lambda }}_{N}^{\left(1\right)}$ and ${\tilde{\lambda }}_{N}^{\left(2\right)}$ consist of all first- and second-order error terms, respectively. It then follows that

(A6) $$\left\{ \begin{array}{l} {\tilde{\lambda }}_{N}^{\left(1\right)}=u_{N}^{H}{\tilde{C}}^{\left(1\right)}u_{N}=O(||\tilde{\xi }|{|}_{2})\;,\\ {\tilde{\lambda }}_{N}^{\left(2\right)}=u_{N}^{H}{\tilde{C}}^{\left(2\right)}u_{N}+u_{N}^{H}{\tilde{C}}^{\left(1\right)}U_{N}{\tilde{C}}^{\left(1\right)}u_{N}=O(||\tilde{\xi }|{|}_{2}^{2})\;. \end{array}\right.$$

Inserting the second equality in (32) into the first equality in (A6) leads to

(A7) $${\tilde{\lambda }}_{N}^{\left(1\right)}=u_{N}^{H}(B_{0}^{H}{\tilde{B}}^{\left(1\right)}+{\tilde{B}}^{(1)H}B_{0})u_{N}=u_{N}^{H}B_{0}^{H}{\tilde{B}}^{\left(1\right)}u_{N}+{\left(u_{N}^{H}B_{0}^{H}{\tilde{B}}^{\left(1\right)}u_{N}\right)}^{H}=O(||\tilde{\xi }|{|}_{2})\;.$$

Substituting the second and third equalities in (32) into the second equality in (A6) leads to

(A8) $$\begin{array}{l} {\tilde{\lambda }}_{N}^{\left(2\right)}=u_{N}^{H}({\tilde{B}}^{(1)H}{\tilde{B}}^{\left(1\right)}+B_{0}^{H}{\tilde{B}}^{\left(2\right)}+{\tilde{B}}^{(2)H}B_{0})u_{N}+u_{N}^{H}(B_{0}^{H}{\tilde{B}}^{\left(1\right)}+{\tilde{B}}^{(1)H}B_{0})U_{N}(B_{0}^{H}{\tilde{B}}^{\left(1\right)}+{\tilde{B}}^{(1)H}B_{0})u_{N}\\ \;=u_{N}^{H}B_{0}^{H}{\tilde{B}}^{\left(1\right)}U_{N}B_{0}^{H}{\tilde{B}}^{\left(1\right)}u_{N}+{\left(u_{N}^{H}B_{0}^{H}{\tilde{B}}^{\left(1\right)}U_{N}B_{0}^{H}{\tilde{B}}^{\left(1\right)}u_{N}\right)}^{H}+u_{N}^{H}{\tilde{B}}^{(1)H}(I_{K}+B_{0}U_{N}B_{0}^{H}){\tilde{B}}^{\left(1\right)}u_{N}\\ \;+u_{N}^{H}B_{0}^{H}{\tilde{B}}^{\left(1\right)}U_{N}{\tilde{B}}^{(1)H}B_{0}u_{N}+u_{N}^{H}B_{0}^{H}{\tilde{B}}^{\left(2\right)}u_{N}+{\left(u_{N}^{H}B_{0}^{H}{\tilde{B}}^{\left(2\right)}u_{N}\right)}^{H}=O({\left||\tilde{\xi }|\right|}_{2}^{2})\;. \end{array}$$

Combining (A7) and (A8) completes the proof.

## Appendix C—Proof of (36) to (38)

From the second equality in (29), it follows for any vectors $z_{1}\in C^{K\times 1}$ and $z_{2}\in C^{N\times 1}$ that

(A9) $$z_{1}^{H}{\tilde{B}}^{\left(1\right)}z_{2}=z_{1}^{H}A^{H}(p)\bar{E}z_{2}+z_{1}^{H}A^{H}(p)\tilde{\Psi }z_{2}+\sum_{l=1}^{L}<\tilde{p}{>}_{l}\cdot z_{1}^{H}{\dot{A}}_{l}^{H}(p){\bar{X}}_{0}z_{2}\;.$$

According to the last equality in (17), it can be readily checked that

(A10) $$z_{1}^{H}A^{H}(p)\bar{E}z_{2}=z_{1}^{H}A^{H}(p)\cdot \mathrm{diag}[z_{2}⊗1_{MK\times 1}]\cdot \bar{\varepsilon }={\left(f_{1}[z_{1},z_{2}]\right)}^{H}{\bar{\varepsilon }}_{c}\;,$$

where $f_{1}[z_{1},z_{2}]$ is given in the first equality in (38). With (21), we have

(A11) $$\begin{array}{cc} z_{1}^{H}A^{H}(p)\tilde{\Psi }z_{2} & =z_{1}^{H}A^{H}(p)\cdot \mathrm{blkdiag}[<z_{2}{>}_{1}\cdot (r_{1}⊗I_{M})\;<z_{2}{>}_{2}\cdot (r_{2}⊗I_{M})\;\cdots \;<z_{2}{>}_{N}\cdot (r_{N}⊗I_{M})]\cdot \tilde{\varphi }\\ & ={\left(f_{2}[z_{1},z_{2}]\right)}^{H}{\tilde{\varphi }}_{c}\;, \end{array}$$

where $f_{2}[z_{1},z_{2}]$ is given in the second equality in (38). In addition, it can be easily verified that

(A12) $$\sum_{l=1}^{L}<\tilde{p}{>}_{l}\cdot z_{1}^{H}{\dot{A}}_{l}^{H}(p){\bar{X}}_{0}z_{2}=[z_{1}^{H}{\dot{A}}_{1}^{H}(p){\bar{X}}_{0}z_{2}\;z_{1}^{H}{\dot{A}}_{2}^{H}(p){\bar{X}}_{0}z_{2}\;\cdots \;z_{1}^{H}{\dot{A}}_{L}^{H}(p){\bar{X}}_{0}z_{2}]\cdot \tilde{p}={\left(f_{3}[z_{1},z_{2}]\right)}^{H}\tilde{p}\;,$$

where $f_{3}[z_{1},z_{2}]$ is given in the third equality in (38). Combining (A9) to (A12) yields

(A13) $$z_{1}^{H}{\tilde{B}}^{\left(1\right)}z_{2}={\left(f_{1}[z_{1},z_{2}]\right)}^{H}{\bar{\varepsilon }}_{c}+{\left(f_{2}[z_{1},z_{2}]\right)}^{H}{\tilde{\varphi }}_{c}+{\left(f_{3}[z_{1},z_{2}]\right)}^{H}\tilde{p}\;.$$

It follows easily from (A13) that

(A14) $$u_{N}^{H}B_{0}^{H}{\tilde{B}}^{\left(1\right)}u_{N}={\left(f_{1}[B_{0}u_{N},u_{N}]\right)}^{H}{\bar{\varepsilon }}_{c}+{\left(f_{2}[B_{0}u_{N},u_{N}]\right)}^{H}{\tilde{\varphi }}_{c}+{\left(f_{3}[B_{0}u_{N},u_{N}]\right)}^{H}\tilde{p}\;,$$

which, combined with (23) and (25), gives

(A15) $$\begin{array}{cc} {\left(u_{N}^{H}B_{0}^{H}{\tilde{B}}^{\left(1\right)}u_{N}\right)}^{H} & ={\left(f_{1}[B_{0}u_{N},u_{N}]\right)}^{T}{\bar{\varepsilon }}_{c}^{*}+{\left(f_{2}[B_{0}u_{N},u_{N}]\right)}^{T}{\tilde{\varphi }}_{c}^{*}+{\left(f_{3}[B_{0}u_{N},u_{N}]\right)}^{T}\tilde{p}\\ & ={\left(f_{1}[B_{0}u_{N},u_{N}]\right)}^{T}\Pi_{\bar{\varepsilon }}{\bar{\varepsilon }}_{c}+{\left(f_{2}[B_{0}u_{N},u_{N}]\right)}^{T}\Pi_{\tilde{\varphi }}{\tilde{\varphi }}_{c}+{\left(f_{3}[B_{0}u_{N},u_{N}]\right)}^{T}\tilde{p}\\ & ={\left(\Pi_{\bar{\varepsilon }}{\left(f_{1}[B_{0}u_{N},u_{N}]\right)}^{*}\right)}^{H}{\bar{\varepsilon }}_{c}+{\left(\Pi_{\tilde{\varphi }}{\left(f_{2}[B_{0}u_{N},u_{N}]\right)}^{*}\right)}^{H}{\tilde{\varphi }}_{c}+{\left(f_{3}[B_{0}u_{N},u_{N}]\right)}^{T}\tilde{p}\;. \end{array}$$

Combining (A14), (A15), and the first equality in (35) completes the proof.

## Appendix D—Proof of (39) to (44)

For any vectors $z_{1}\in C^{K\times 1}$ and $z_{2}\in C^{N\times 1}$ and matrix $Z\in C^{N\times K}$, it is straightforward to obtain that

(A16) $$z_{1}^{H}{\tilde{B}}^{\left(1\right)}Z{\tilde{B}}^{\left(1\right)}z_{2}=\sum_{k=1}^{K}\left(z_{1}^{H}{\tilde{B}}^{\left(1\right)}Zi_{K}^{\left(k\right)})(i_{K}^{(k)H}{\tilde{B}}^{\left(1\right)}z_{2}\right)\;.$$

Inserting (A13) into (A16) produces

(A17) $$\begin{array}{l} z_{1}^{H}{\tilde{B}}^{\left(1\right)}Z{\tilde{B}}^{\left(1\right)}z_{2}\\ =\sum_{k=1}^{K}\left({\left(f_{1}[z_{1},Zi_{K}^{\left(k\right)}]\right)}^{H}{\bar{\varepsilon }}_{c}+{\left(f_{2}[z_{1},Zi_{K}^{\left(k\right)}]\right)}^{H}{\tilde{\varphi }}_{c}+{\left(f_{3}[z_{1},Zi_{K}^{\left(k\right)}]\right)}^{H}\tilde{p})({\left(f_{1}[i_{K}^{\left(k\right)},z_{2}]\right)}^{H}{\bar{\varepsilon }}_{c}+{\left(f_{2}[i_{K}^{\left(k\right)},z_{2}]\right)}^{H}{\tilde{\varphi }}_{c}+{\left(f_{3}[i_{K}^{\left(k\right)},z_{2}]\right)}^{H}\tilde{p}\right)\\ ={\bar{\varepsilon }}_{c}^{H}\left(\sum_{k=1}^{K}\Pi_{\bar{\varepsilon }}{\left(f_{1}[z_{1},Zi_{K}^{\left(k\right)}]\right)}^{*}{\left(f_{1}[i_{K}^{\left(k\right)},z_{2}]\right)}^{H}\right){\bar{\varepsilon }}_{c}+{\tilde{\varphi }}_{c}^{H}\left(\sum_{k=1}^{K}\Pi_{\tilde{\varphi }}{\left(f_{2}[z_{1},Zi_{K}^{\left(k\right)}]\right)}^{*}{\left(f_{2}[i_{K}^{\left(k\right)},z_{2}]\right)}^{H}\right){\tilde{\varphi }}_{c}+{\tilde{p}}^{T}\left(\sum_{k=1}^{K}{\left(f_{3}[z_{1},Zi_{K}^{\left(k\right)}]\right)}^{*}{\left(f_{3}[i_{K}^{\left(k\right)},z_{2}]\right)}^{H}\right)\tilde{p}\\ +{\bar{\varepsilon }}_{c}^{H}\left(\sum_{k=1}^{K}\Pi_{\bar{\varepsilon }}({\left(f_{1}[z_{1},Zi_{K}^{\left(k\right)}]\right)}^{*}{\left(f_{2}[i_{K}^{\left(k\right)},z_{2}]\right)}^{H}+{\left(f_{1}[i_{K}^{\left(k\right)},z_{2}]\right)}^{*}{\left(f_{2}[z_{1},Zi_{K}^{\left(k\right)}]\right)}^{H})\right){\tilde{\varphi }}_{c}\\ +{\bar{\varepsilon }}_{c}^{H}\left(\sum_{k=1}^{K}\Pi_{\bar{\varepsilon }}({\left(f_{1}[z_{1},Zi_{K}^{\left(k\right)}]\right)}^{*}{\left(f_{3}[i_{K}^{\left(k\right)},z_{2}]\right)}^{H}+{\left(f_{1}[i_{K}^{\left(k\right)},z_{2}]\right)}^{*}{\left(f_{3}[z_{1},Zi_{K}^{\left(k\right)}]\right)}^{H})\right)\tilde{p}\\ +{\tilde{\varphi }}_{c}^{H}\left(\sum_{k=1}^{K}\Pi_{\tilde{\varphi }}({\left(f_{2}[z_{1},Zi_{K}^{\left(k\right)}]\right)}^{*}{\left(f_{3}[i_{K}^{\left(k\right)},z_{2}]\right)}^{H}+{\left(f_{2}[i_{K}^{\left(k\right)},z_{2}]\right)}^{*}{\left(f_{3}[z_{1},Zi_{K}^{\left(k\right)}]\right)}^{H})\right)\tilde{p}\\ ={\bar{\varepsilon }}_{c}^{H}\cdot F_{a1}[z_{1},Z,z_{2}]\cdot {\bar{\varepsilon }}_{c}+{\tilde{\varphi }}_{c}^{H}\cdot F_{a2}[z_{1},Z,z_{2}]\cdot {\tilde{\varphi }}_{c}+{\tilde{p}}^{T}\cdot F_{a3}[z_{1},Z,z_{2}]\cdot \tilde{p}+{\bar{\varepsilon }}_{c}^{H}\cdot F_{a4}[z_{1},Z,z_{2}]\cdot {\tilde{\varphi }}_{c}\\ +{\bar{\varepsilon }}_{c}^{H}\cdot F_{a5}[z_{1},Z,z_{2}]\cdot \tilde{p}+{\tilde{\varphi }}_{c}^{H}\cdot F_{a6}[z_{1},Z,z_{2}]\cdot \tilde{p}\;, \end{array}$$

where ${\left\{F_{ak}[\cdot \;,\;\cdot ,\cdot ]\right\}}_{1\leq k\leq 6}$ are given in (41).

For any vectors $z_{1}\in C^{N\times 1}$ and $z_{2}\in C^{N\times 1}$ and matrix $Z\in C^{K\times K}$, it can be readily checked that

(A18) $$z_{1}^{H}{\tilde{B}}^{(1)H}Z{\tilde{B}}^{\left(1\right)}z_{2}=\sum_{k=1}^{K}\left(z_{1}^{H}{\tilde{B}}^{(1)H}Zi_{K}^{\left(k\right)})(i_{K}^{(k)H}{\tilde{B}}^{\left(1\right)}z_{2}\right)=\sum_{k=1}^{K}{\left({\left(Zi_{K}^{\left(k\right)}\right)}^{H}{\tilde{B}}^{\left(1\right)}z_{1}\right)}^{H}(i_{K}^{(k)H}{\tilde{B}}^{\left(1\right)}z_{2})\;.$$

Putting (A13) into (A18) gives

(A19) $$\begin{array}{l} z_{1}^{H}{\tilde{B}}^{(1)H}Z{\tilde{B}}^{\left(1\right)}z_{2}\\ =\sum_{k=1}^{K}{\left({\left(f_{1}[Zi_{K}^{\left(k\right)},z_{1}]\right)}^{H}{\bar{\varepsilon }}_{c}+{\left(f_{2}[Zi_{K}^{\left(k\right)},z_{1}]\right)}^{H}{\tilde{\varphi }}_{c}+{\left(f_{3}[Zi_{K}^{\left(k\right)},z_{1}]\right)}^{H}\tilde{p}\right)}^{H}({\left(f_{1}[i_{K}^{\left(k\right)},z_{2}]\right)}^{H}{\bar{\varepsilon }}_{c}+{\left(f_{2}[i_{K}^{\left(k\right)},z_{2}]\right)}^{H}{\tilde{\varphi }}_{c}+{\left(f_{3}[i_{K}^{\left(k\right)},z_{2}]\right)}^{H}\tilde{p})\\ ={\bar{\varepsilon }}_{c}^{H}\left(\sum_{k=1}^{K}f_{1}[Zi_{K}^{\left(k\right)},z_{1}]\cdot {\left(f_{1}[i_{K}^{\left(k\right)},z_{2}]\right)}^{H}\right){\bar{\varepsilon }}_{c}+{\tilde{\varphi }}_{c}^{H}\left(\sum_{k=1}^{K}f_{2}[Zi_{K}^{\left(k\right)},z_{1}]\cdot {\left(f_{2}[i_{K}^{\left(k\right)},z_{2}]\right)}^{H}\right){\tilde{\varphi }}_{c}+{\tilde{p}}^{T}\left(\sum_{k=1}^{K}f_{3}[Zi_{K}^{\left(k\right)},z_{1}]\cdot {\left(f_{3}[i_{K}^{\left(k\right)},z_{2}]\right)}^{H}\right)\tilde{p}\\ +{\bar{\varepsilon }}_{c}^{H}\left(\sum_{k=1}^{K}\left(f_{1}[Zi_{K}^{\left(k\right)},z_{1}]\cdot {\left(f_{2}[i_{K}^{\left(k\right)},z_{2}]\right)}^{H}+\Pi_{{\bar{\varepsilon }}_{c}}{\left(f_{1}[i_{K}^{\left(k\right)},z_{2}]\right)}^{*}{\left(f_{2}[Zi_{K}^{\left(k\right)},z_{1}]\right)}^{T}\Pi_{\tilde{\varphi }}\right)\right){\tilde{\varphi }}_{c}\\ +{\bar{\varepsilon }}_{c}^{H}\left(\sum_{k=1}^{K}\left(f_{1}[Zi_{K}^{\left(k\right)},z_{1}]\cdot {\left(f_{3}[i_{K}^{\left(k\right)},z_{2}]\right)}^{H}+\Pi_{{\bar{\varepsilon }}_{c}}{\left(f_{1}[i_{K}^{\left(k\right)},z_{2}]\right)}^{*}{\left(f_{3}[Zi_{K}^{\left(k\right)},z_{1}]\right)}^{T}\right)\right)\tilde{p}\\ +{\tilde{\varphi }}_{c}^{H}\left(\sum_{k=1}^{K}\left(f_{2}[Zi_{K}^{\left(k\right)},z_{1}]\cdot {\left(f_{3}[i_{K}^{\left(k\right)},z_{2}]\right)}^{H}+\Pi_{\tilde{\varphi }}{\left(f_{2}[i_{K}^{\left(k\right)},z_{2}]\right)}^{*}{\left(f_{3}[Zi_{K}^{\left(k\right)},z_{1}]\right)}^{T}\right)\right)\tilde{p}\\ ={\bar{\varepsilon }}_{c}^{H}\cdot F_{b1}[z_{1},Z,z_{2}]\cdot {\bar{\varepsilon }}_{c}+{\tilde{\varphi }}_{c}^{H}\cdot F_{b2}[z_{1},Z,z_{2}]\cdot {\tilde{\varphi }}_{c}+{\tilde{p}}^{T}\cdot F_{b3}[z_{1},Z,z_{2}]\cdot \tilde{p}+{\bar{\varepsilon }}_{c}^{H}\cdot F_{b4}[z_{1},Z,z_{2}]\cdot {\tilde{\varphi }}_{c}\\ +{\bar{\varepsilon }}_{c}^{H}\cdot F_{b5}[z_{1},Z,z_{2}]\cdot \tilde{p}+{\tilde{\varphi }}_{c}^{H}\cdot F_{b6}[z_{1},Z,z_{2}]\cdot \tilde{p}\;, \end{array}$$

where ${\left\{F_{bk}[\cdot \;,\;\cdot ,\cdot ]\right\}}_{1\leq k\leq 6}$ are given in (42).

For any vectors $z_{1}\in C^{K\times 1}$ and $z_{2}\in C^{K\times 1}$ and matrix $Z\in C^{N\times N}$, it is straightforward to deduce that

(A20) $$z_{1}^{H}{\tilde{B}}^{\left(1\right)}Z{\tilde{B}}^{(1)H}z_{2}=\sum_{n=1}^{N}\left(z_{1}^{H}{\tilde{B}}^{\left(1\right)}Zi_{N}^{\left(n\right)})(i_{N}^{(n)H}{\tilde{B}}^{(1)H}z_{2}\right)=\sum_{n=1}^{N}(z_{1}^{H}{\tilde{B}}^{\left(1\right)}Zi_{N}^{\left(n\right)}){\left(z_{2}^{H}{\tilde{B}}^{\left(1\right)}i_{N}^{\left(n\right)}\right)}^{H}\;.$$

The substitution of (A13) into (A20) leads to

(A21) $$\begin{array}{l} z_{1}^{H}{\tilde{B}}^{\left(1\right)}Z{\tilde{B}}^{(1)H}z_{2}\\ =\sum_{n=1}^{N}({\left(f_{1}[z_{1},Zi_{N}^{\left(n\right)}]\right)}^{H}{\bar{\varepsilon }}_{c}+{\left(f_{2}[z_{1},Zi_{N}^{\left(n\right)}]\right)}^{H}{\tilde{\varphi }}_{c}+{\left(f_{3}[z_{1},Zi_{N}^{\left(n\right)}]\right)}^{H}\tilde{p}){\left({\left(f_{1}[z_{2},i_{N}^{\left(n\right)}]\right)}^{H}{\bar{\varepsilon }}_{c}+{\left(f_{2}[z_{2},i_{N}^{\left(n\right)}]\right)}^{H}{\tilde{\varphi }}_{c}+{\left(f_{3}[z_{2},i_{N}^{\left(n\right)}]\right)}^{H}\tilde{p}\right)}^{H}\\ ={\bar{\varepsilon }}_{c}^{H}\left(\sum_{n=1}^{N}f_{1}[z_{2},i_{N}^{\left(n\right)}]\cdot {\left(f_{1}[z_{1},Zi_{N}^{\left(n\right)}]\right)}^{H}\right){\bar{\varepsilon }}_{c}+{\tilde{\varphi }}_{c}^{H}\left(\sum_{n=1}^{N}f_{2}[z_{2},i_{N}^{\left(n\right)}]\cdot {\left(f_{2}[z_{1},Zi_{N}^{\left(n\right)}]\right)}^{H}\right){\tilde{\varphi }}_{c}+{\tilde{p}}^{T}\left(\sum_{n=1}^{N}f_{3}[z_{2},i_{N}^{\left(n\right)}]\cdot {\left(f_{3}[z_{1},Zi_{N}^{\left(n\right)}]\right)}^{H}\right)\tilde{p}\\ +{\bar{\varepsilon }}_{c}^{H}\left(\sum_{n=1}^{N}\left(f_{1}[z_{2},i_{N}^{\left(n\right)}]\cdot {\left(f_{2}[z_{1},Zi_{N}^{\left(n\right)}]\right)}^{H}+\Pi_{{\bar{\varepsilon }}_{c}}{\left(f_{1}[z_{1},Zi_{N}^{\left(n\right)}]\right)}^{*}{\left(f_{2}[z_{2},i_{N}^{\left(n\right)}]\right)}^{T}\Pi_{{\tilde{\varphi }}_{c}}\right)\right){\tilde{\varphi }}_{c}\\ +{\bar{\varepsilon }}_{c}^{H}\left(\sum_{n=1}^{N}\left(f_{1}[z_{2},i_{N}^{\left(n\right)}]\cdot {\left(f_{3}[z_{1},Zi_{N}^{\left(n\right)}]\right)}^{H}+\Pi_{{\bar{\varepsilon }}_{c}}{\left(f_{1}[z_{1},Zi_{N}^{\left(n\right)}]\right)}^{*}{\left(f_{3}[z_{2},i_{N}^{\left(n\right)}]\right)}^{T}\right)\right)\tilde{p}\\ +{\tilde{\varphi }}_{c}^{H}\left(\sum_{n=1}^{N}\left(f_{2}[z_{2},i_{N}^{\left(n\right)}]\cdot {\left(f_{3}[z_{1},Zi_{N}^{\left(n\right)}]\right)}^{H}+\Pi_{{\tilde{\varphi }}_{c}}{\left(f_{2}[z_{1},Zi_{N}^{\left(n\right)}]\right)}^{*}{\left(f_{3}[z_{2},i_{N}^{\left(n\right)}]\right)}^{T}\right)\right)\tilde{p}\\ ={\bar{\varepsilon }}_{c}^{H}\cdot F_{c1}[z_{1},Z,z_{2}]\cdot {\bar{\varepsilon }}_{c}+{\tilde{\varphi }}_{c}^{H}\cdot F_{c2}[z_{1},Z,z_{2}]\cdot {\tilde{\varphi }}_{c}+{\tilde{p}}^{T}\cdot F_{c3}[z_{1},Z,z_{2}]\cdot \tilde{p}+{\bar{\varepsilon }}_{c}^{H}\cdot F_{c4}[z_{1},Z,z_{2}]\cdot {\tilde{\varphi }}_{c}\\ +{\bar{\varepsilon }}_{c}^{H}\cdot F_{c5}[z_{1},Z,z_{2}]\cdot \tilde{p}+{\tilde{\varphi }}_{c}^{H}\cdot F_{c6}[z_{1},Z,z_{2}]\cdot \tilde{p}\;, \end{array}$$

where ${\left\{F_{ck}[\cdot \;,\;\cdot ,\cdot ]\right\}}_{1\leq k\leq 6}$ are given in (43).

For any vectors $z_{1}\in C^{K\times 1}$ and $z_{2}\in C^{N\times 1}$, it can be easily verified from the third equality in (29) that

(A22) $$z_{1}^{H}{\tilde{B}}^{\left(2\right)}z_{2}=\sum_{l=1}^{L}<\tilde{p}{>}_{l}\cdot z_{1}^{H}{\dot{A}}_{l}^{H}(p)\bar{E}z_{2}+\sum_{l=1}^{L}<\tilde{p}{>}_{l}\cdot z_{1}^{H}{\dot{A}}_{l}^{H}(p)\tilde{\Psi }z_{2}+\frac{1}{2}\cdot \sum_{l_{1}=1}^{L}\sum_{l_{2}=1}^{L}<\tilde{p}{>}_{l_{1}}\cdot <\tilde{p}{>}_{l_{2}}\cdot z_{1}^{H}{\ddot{A}}_{l_{1}l_{2}}^{H}(p){\bar{X}}_{0}z_{2}\;.$$

According to the last equality in (17), we get

(A23) $$\begin{array}{l} \sum_{l=1}^{L}<\tilde{p}{>}_{l}\cdot z_{1}^{H}{\dot{A}}_{l}^{H}(p)\bar{E}z_{2}={\bar{\varepsilon }}^{T}\cdot \mathrm{diag}[z_{2}⊗1_{MK\times 1}]\cdot [{\dot{A}}_{1}^{*}(p)z_{1}^{*}\;{\dot{A}}_{2}^{*}(p)z_{1}^{*}\;\cdots \;{\dot{A}}_{L}^{*}(p)z_{1}^{*}]\cdot \tilde{p}\\ ={\bar{\varepsilon }}_{c}^{H}\cdot G_{1}[z_{1},z_{2}]\cdot \tilde{p}\;, \end{array}$$

where $G_{1}[z_{1},z_{2}]$ is given in the first equality in (44). It follows from (21) that

(A24) $$\begin{array}{l} \sum_{l=1}^{L}<\tilde{p}{>}_{l}\cdot z_{1}^{H}{\dot{A}}_{l}^{H}(p)\tilde{\Psi }z_{2}\\ ={\tilde{\varphi }}^{T}\cdot \mathrm{blkdiag}[<z_{2}{>}_{1}\cdot (r_{1}^{T}⊗I_{M})\;<z_{2}{>}_{2}\cdot (r_{2}^{T}⊗I_{M})\;\cdots \;<z_{2}{>}_{N}\cdot (r_{N}^{T}⊗I_{M})]\cdot [{\dot{A}}_{1}^{*}(p)z_{1}^{*}\;{\dot{A}}_{2}^{*}(p)z_{1}^{*}\;\cdots \;{\dot{A}}_{L}^{*}(p)z_{1}^{*}]\cdot \tilde{p}\\ ={\tilde{\varphi }}_{c}^{H}\cdot G_{2}[z_{1},z_{2}]\cdot \tilde{p}\;, \end{array}$$

where $G_{2}[z_{1},z_{2}]$ is given in the second equality in (44). In addition, it can be easily verified that

(A25) $$\begin{array}{l} \frac{1}{2}\cdot \sum_{l_{1}=1}^{L}\sum_{l_{2}=1}^{L}<\tilde{p}{>}_{l_{1}}\cdot <\tilde{p}{>}_{l_{2}}\cdot z_{1}^{H}{\ddot{A}}_{l_{1}l_{2}}^{H}(p){\bar{X}}_{0}z_{2}\\ =\frac{1}{2}\cdot {\tilde{p}}^{T}\cdot \left[\begin{array}{cccc} z_{1}^{H}{\ddot{A}}_{11}^{H}(p){\bar{X}}_{0}z_{2} & z_{1}^{H}{\ddot{A}}_{12}^{H}(p){\bar{X}}_{0}z_{2} & \cdots & z_{1}^{H}{\ddot{A}}_{1L}^{H}(p){\bar{X}}_{0}z_{2}\\ z_{1}^{H}{\ddot{A}}_{21}^{H}(p){\bar{X}}_{0}z_{2} & z_{1}^{H}{\ddot{A}}_{22}^{H}(p){\bar{X}}_{0}z_{2} & \cdots & z_{1}^{H}{\ddot{A}}_{2L}^{H}(p){\bar{X}}_{0}z_{2}\\ \vdots & \vdots & \ddots & \vdots \\ z_{1}^{H}{\ddot{A}}_{L1}^{H}(p){\bar{X}}_{0}z_{2} & z_{1}^{H}{\ddot{A}}_{L2}^{H}(p){\bar{X}}_{0}z_{2} & \cdots & z_{1}^{H}{\ddot{A}}_{LL}^{H}(p){\bar{X}}_{0}z_{2} \end{array}\right]\cdot \tilde{p}={\tilde{p}}^{T}\cdot G_{3}[z_{1},z_{2}]\cdot \tilde{p}\;, \end{array}$$

where $G_{3}[z_{1},z_{2}]$ is given in the third equality in (44). Combining (A22) to (A25) gives

(A26) $$z_{1}^{H}{\tilde{B}}^{\left(2\right)}z_{2}={\bar{\varepsilon }}_{c}^{H}\cdot G_{1}[z_{1},z_{2}]\cdot \tilde{p}+{\tilde{\varphi }}_{c}^{H}\cdot G_{2}[z_{1},z_{2}]\cdot \tilde{p}+{\tilde{p}}^{T}\cdot G_{3}[z_{1},z_{2}]\cdot \tilde{p}\;.$$

With (A17) we have

(A27) $$\begin{array}{l} u_{N}^{H}B_{0}^{H}{\tilde{B}}^{\left(1\right)}U_{N}B_{0}^{H}{\tilde{B}}^{\left(1\right)}u_{N}\\ ={\bar{\varepsilon }}_{c}^{H}\cdot F_{a1}[B_{0}u_{N},U_{N}B_{0}^{H},u_{N}]\cdot {\bar{\varepsilon }}_{c}+{\tilde{\varphi }}_{c}^{H}\cdot F_{a2}[B_{0}u_{N},U_{N}B_{0}^{H},u_{N}]\cdot {\tilde{\varphi }}_{c}+{\tilde{p}}^{T}\cdot F_{a3}[B_{0}u_{N},U_{N}B_{0}^{H},u_{N}]\cdot \tilde{p}\\ +{\bar{\varepsilon }}_{c}^{H}\cdot F_{a4}[B_{0}u_{N},U_{N}B_{0}^{H},u_{N}]\cdot {\tilde{\varphi }}_{c}+{\bar{\varepsilon }}_{c}^{H}\cdot F_{a5}[B_{0}u_{N},U_{N}B_{0}^{H},u_{N}]\cdot \tilde{p}+{\tilde{\varphi }}_{c}^{H}\cdot F_{a6}[B_{0}u_{N},U_{N}B_{0}^{H},u_{N}]\cdot \tilde{p}\;, \end{array}$$

which, together with (23) and (25), gives

(A28) $$\begin{array}{l} {\left(u_{N}^{H}B_{0}^{H}{\tilde{B}}^{\left(1\right)}U_{N}B_{0}^{H}{\tilde{B}}^{\left(1\right)}u_{N}\right)}^{H}\\ ={\bar{\varepsilon }}_{c}^{H}{\left(F_{a1}[B_{0}u_{N},U_{N}B_{0}^{H},u_{N}]\right)}^{H}{\bar{\varepsilon }}_{c}+{\tilde{\varphi }}_{c}^{H}{\left(F_{a2}[B_{0}u_{N},U_{N}B_{0}^{H},u_{N}]\right)}^{H}{\tilde{\varphi }}_{c}+{\tilde{p}}^{T}{\left(F_{a3}[B_{0}u_{N},U_{N}B_{0}^{H},u_{N}]\right)}^{H}\tilde{p}\\ +{\bar{\varepsilon }}_{c}^{T}{\left(F_{a4}[B_{0}u_{N},U_{N}B_{0}^{H},u_{N}]\right)}^{*}{\tilde{\varphi }}_{c}^{*}+{\bar{\varepsilon }}_{c}^{T}{\left(F_{a5}[B_{0}u_{N},U_{N}B_{0}^{H},u_{N}]\right)}^{*}\tilde{p}+{\tilde{\varphi }}_{c}^{T}{\left(F_{a6}[B_{0}u_{N},U_{N}B_{0}^{H},u_{N}]\right)}^{*}\tilde{p}\\ ={\bar{\varepsilon }}_{c}^{H}{\left(F_{a1}[B_{0}u_{N},U_{N}B_{0}^{H},u_{N}]\right)}^{H}{\bar{\varepsilon }}_{c}+{\tilde{\varphi }}_{c}^{H}{\left(F_{a2}[B_{0}u_{N},U_{N}B_{0}^{H},u_{N}]\right)}^{H}{\tilde{\varphi }}_{c}+{\tilde{p}}^{T}{\left(F_{a3}[B_{0}u_{N},U_{N}B_{0}^{H},u_{N}]\right)}^{H}\tilde{p}\\ +{\bar{\varepsilon }}_{c}^{H}\Pi_{\bar{\varepsilon }}{\left(F_{a4}[B_{0}u_{N},U_{N}B_{0}^{H},u_{N}]\right)}^{*}\Pi_{\tilde{\varphi }}{\tilde{\varphi }}_{c}+{\bar{\varepsilon }}_{c}^{H}\Pi_{\bar{\varepsilon }}{\left(F_{a5}[B_{0}u_{N},U_{N}B_{0}^{H},u_{N}]\right)}^{*}\tilde{p}+{\tilde{\varphi }}_{c}^{H}\Pi_{\tilde{\varphi }}{\left(F_{a6}[B_{0}u_{N},U_{N}B_{0}^{H},u_{N}]\right)}^{*}\tilde{p}\;. \end{array}$$

Applying (A19), it can be shown that

(A29) $$\begin{array}{l} u_{N}^{H}{\tilde{B}}^{(1)H}(I_{K}+B_{0}U_{N}B_{0}^{H}){\tilde{B}}^{\left(1\right)}u_{N}\\ ={\bar{\varepsilon }}_{c}^{H}\cdot F_{b1}[u_{N},I_{K}+B_{0}U_{N}B_{0}^{H},u_{N}]\cdot {\bar{\varepsilon }}_{c}+{\tilde{\varphi }}_{c}^{H}\cdot F_{b2}[u_{N},I_{K}+B_{0}U_{N}B_{0}^{H},u_{N}]\cdot {\tilde{\varphi }}_{c}\\ +{\tilde{p}}^{T}\cdot F_{b3}[u_{N},I_{K}+B_{0}U_{N}B_{0}^{H},u_{N}]\cdot \tilde{p}+{\bar{\varepsilon }}_{c}^{H}\cdot F_{b4}[u_{N},I_{K}+B_{0}U_{N}B_{0}^{H},u_{N}]\cdot {\tilde{\varphi }}_{c}\\ +{\bar{\varepsilon }}_{c}^{H}\cdot F_{b5}[u_{N},I_{K}+B_{0}U_{N}B_{0}^{H},u_{N}]\cdot \tilde{p}+{\tilde{\varphi }}_{c}^{H}\cdot F_{b6}[u_{N},I_{K}+B_{0}U_{N}B_{0}^{H},u_{N}]\cdot \tilde{p}\;. \end{array}$$

According to (A21), we have

(A30) $$\begin{array}{l} u_{N}^{H}B_{0}^{H}{\tilde{B}}^{\left(1\right)}U_{N}{\tilde{B}}^{(1)H}B_{0}u_{N}\\ ={\bar{\varepsilon }}_{c}^{H}\cdot F_{c1}[B_{0}u_{N},U_{N},B_{0}u_{N}]\cdot {\bar{\varepsilon }}_{c}+{\tilde{\varphi }}_{c}^{H}\cdot F_{c2}[B_{0}u_{N},U_{N},B_{0}u_{N}]\cdot {\tilde{\varphi }}_{c}+{\tilde{p}}^{T}\cdot F_{c3}[B_{0}u_{N},U_{N},B_{0}u_{N}]\cdot \tilde{p}\\ +{\bar{\varepsilon }}_{c}^{H}\cdot F_{c4}[B_{0}u_{N},U_{N},B_{0}u_{N}]\cdot {\tilde{\varphi }}_{c}+{\bar{\varepsilon }}_{c}^{H}\cdot F_{c5}[B_{0}u_{N},U_{N},B_{0}u_{N}]\cdot \tilde{p}+{\tilde{\varphi }}_{c}^{H}\cdot F_{c6}[B_{0}u_{N},U_{N},B_{0}u_{N}]\cdot \tilde{p}\;. \end{array}$$

Additionally, it follows from (A26) that

(A31) $$u_{N}^{H}B_{0}^{H}{\tilde{B}}^{\left(2\right)}u_{N}={\bar{\varepsilon }}_{c}^{H}\cdot G_{1}[B_{0}u_{N},u_{N}]\cdot \tilde{p}+{\tilde{\varphi }}_{c}^{H}\cdot G_{2}[B_{0}u_{N},u_{N}]\cdot \tilde{p}+{\tilde{p}}^{T}\cdot G_{3}[B_{0}u_{N},u_{N}]\cdot \tilde{p}\;,$$

which, together with (23) and (25), gives

(A32) $$\begin{array}{cc} {\left(u_{N}^{H}B_{0}^{H}{\tilde{B}}^{\left(2\right)}u_{N}\right)}^{H} & ={\bar{\varepsilon }}_{c}^{T}{\left(G_{1}[B_{0}u_{N},u_{N}]\right)}^{*}\tilde{p}+{\tilde{\varphi }}_{c}^{T}{\left(G_{2}[B_{0}u_{N},u_{N}]\right)}^{*}\tilde{p}+{\tilde{p}}^{T}{\left(G_{3}[B_{0}u_{N},u_{N}]\right)}^{*}\tilde{p}\\ & ={\bar{\varepsilon }}_{c}^{H}\Pi_{\bar{\varepsilon }}{\left(G_{1}[B_{0}u_{N},u_{N}]\right)}^{*}\tilde{p}+{\tilde{\varphi }}_{c}^{H}\Pi_{\tilde{\varphi }}{\left(G_{2}[B_{0}u_{N},u_{N}]\right)}^{*}\tilde{p}+{\tilde{p}}^{T}{\left(G_{3}[B_{0}u_{N},u_{N}]\right)}^{*}\tilde{p}\;. \end{array}$$

Combining (A27) to (A32) and the second equality in (35) completes the proof.

## Appendix E—Proof of Proposition 2

First introduce the event ${z^{\prime }}_{2}\in \{z_{2}|z_{1}\leq \alpha_{1}\}$. The joint probability can then be expressed as

(A33) $$\mathrm{Pr}\{z_{1}\leq \alpha_{1}\;,\;z_{2}\leq \alpha_{2}\}=\mathrm{Pr}\{z_{1}\leq \alpha_{1}\}\cdot \mathrm{Pr}\{{z^{\prime }}_{2}\leq \alpha_{2}\}\;.$$

It is obvious that

(A34) $$\begin{array}{cc} \mathrm{Pr}\{z_{1}\leq \alpha_{1}\} & =\int_{-\infty }^{\alpha_{1}}\frac{1}{\sqrt{2\pi v_{11}}}\cdot \exp\{-{\left(t-m_{1}\right)}^{2}/(2v_{11})\}\cdot dt\\ & =\int_{-\infty }^{(\alpha_{1}-m_{1})/\sqrt{v_{11}}}\frac{1}{\sqrt{2\pi }}\cdot \exp\{-t^{2}/2\}\cdot dt=\Gamma_{0}[\alpha_{10}/\sqrt{v_{11}}]\;. \end{array}$$

Additionally, random variable $z_{2}$ can be decomposed with classical minimum-MSE theory into

(A35) $$z_{2}=-\frac{1}{v_{11}}\cdot z_{0}+\frac{v_{12}}{v_{11}}\cdot (z_{1}-m_{1})+m_{2}\;,$$

where $z_{0}$ is drawn from a zero-mean Gaussian distribution, independent of $z_{1}$, with variance

(A36) $$\mathrm{var}[z_{0}]=E[z_{0}^{2}]=v_{11}(v_{11}v_{22}-v_{12}^{2})\;.$$

According to (A35), it can be verified that

(A37) $$\begin{array}{ll} E[z_{2}|z_{1}\leq \alpha_{1}] & =-\frac{1}{v_{11}}\cdot E[z_{0}|z_{1}\leq \alpha_{1}]+\frac{v_{12}}{v_{11}}\cdot E[(z_{1}-m_{1})|z_{1}\leq \alpha_{1}]+m_{2}\\ & =\frac{v_{12}}{v_{11}}\cdot E[z_{10}|z_{10}\leq \alpha_{10}]+m_{2}\;, \end{array}$$

(A38) $$\begin{array}{l} E[z_{2}^{2}|z_{1}\leq \alpha_{1}]=\frac{1}{v_{11}^{2}}\cdot E[z_{0}^{2}|z_{1}\leq \alpha_{1}]+\frac{v_{12}^{2}}{v_{11}^{2}}\cdot E[{\left(z_{1}-m_{1}\right)}^{2}|z_{1}\leq \alpha_{1}]-\frac{2m_{2}}{v_{11}}\cdot E[z_{0}|z_{1}\leq \alpha_{1}]\\ \;-\frac{2v_{12}}{v_{11}^{2}}\cdot E[z_{0}(z_{1}-m_{1})|z_{1}\leq \alpha_{1}]+\frac{2m_{2}v_{12}}{v_{11}}\cdot E[(z_{1}-m_{1})|z_{1}\leq \alpha_{1}]+m_{2}^{2}\\ \;=\frac{v_{11}v_{22}-v_{12}^{2}}{v_{11}}+\frac{v_{12}^{2}}{v_{11}^{2}}\cdot E[z_{10}^{2}|z_{10}\leq \alpha_{10}]+\frac{2m_{2}v_{12}}{v_{11}}\cdot E[z_{10}|z_{10}\leq \alpha_{10}]+m_{2}^{2}\;, \end{array}$$

where $z_{10}=z_{1}-m_{1}$ and $\alpha_{10}=\alpha_{1}-m_{1}$. Applying the incomplete moment theory presented in [28], we get

(A39) $$E[z_{10}|z_{10}\leq \alpha_{10}]=\frac{\int_{-\infty }^{\alpha_{10}}\frac{t}{\sqrt{2\pi v_{11}}}\cdot \exp\{-t^{2}/(2v_{11})\}\cdot dt}{\mathrm{Pr}\{z_{10}<\alpha_{10}\}}=-\frac{\sqrt{v_{11}}\cdot \exp\{-\alpha_{10}^{2}/(2v_{11})\}}{\sqrt{2\pi }\cdot \Gamma_{0}[\alpha_{10}/\sqrt{v_{11}}]}\;,$$

(A40) $$E[z_{10}^{2}|z_{10}\leq \alpha_{10}]=\frac{\int_{-\infty }^{\alpha_{10}}\frac{t^{2}}{\sqrt{2\pi v_{11}}}\cdot \exp\{-t^{2}/(2v_{11})\}\cdot dt}{\mathrm{Pr}\{z_{10}<\alpha_{10}\}}=v_{11}-\sqrt{\frac{v_{11}}{2\pi }}\cdot \frac{\alpha_{10}\cdot \exp\{-\alpha_{10}^{2}/(2v_{11})\}}{\Gamma_{0}[\alpha_{10}/\sqrt{v_{11}}]}\;.$$

Inserting (A39) back into (A37) yields

(A41) $$E[{\bar{z}}_{2}]=E[z_{2}|z_{1}\leq \alpha_{1}]=m_{2}-\frac{v_{12}\cdot \exp\{-\alpha_{10}^{2}/(2v_{11})\}}{\sqrt{2\pi v_{11}}\cdot \Gamma_{0}[\alpha_{10}/\sqrt{v_{11}}]}\;.$$

Furthermore, substituting (A39) and (A40) into (A38) leads to

(A42) $$\begin{array}{cc} E[{\bar{z}}_{2}^{2}]=E[z_{2}^{2}|z_{1}\leq \alpha_{1}] & =\frac{v_{11}v_{22}-v_{12}^{2}}{v_{11}}+\frac{v_{12}^{2}}{v_{11}^{2}}\cdot E[z_{10}^{2}|z_{10}\leq \alpha_{10}]+\frac{2m_{2}v_{12}}{v_{11}}\cdot E[z_{10}|z_{10}\leq \alpha_{10}]+m_{2}^{2}\\ & =v_{22}-\frac{v_{12}^{2}}{\sqrt{2\pi v_{11}}}\cdot \frac{\alpha_{10}\cdot \exp\{-\alpha_{10}^{2}/(2v_{11})\}}{v_{11}\cdot \Gamma_{0}[\alpha_{10}/\sqrt{v_{11}}]}-\frac{2m_{2}v_{12}}{\sqrt{2\pi v_{11}}}\cdot \frac{\exp\{-\alpha_{10}^{2}/(2v_{11})\}}{\Gamma_{0}[\alpha_{10}/\sqrt{v_{11}}]}+m_{2}^{2}\;, \end{array}$$

which together with (A41) gives

(A43) $$\begin{array}{cc} \mathrm{var}[{\bar{z}}_{2}] & =E[{\bar{z}}_{2}^{2}]-{\left(E[{\bar{z}}_{2}]\right)}^{2}\\ & =v_{22}-\frac{v_{12}^{2}}{\sqrt{2\pi }v_{11}\cdot \Gamma_{0}[\alpha_{10}/\sqrt{v_{11}}]}\cdot \left(\frac{\alpha_{10}\cdot \exp\{-\alpha_{10}^{2}/(2v_{11})\}}{\sqrt{v_{11}}}+\frac{\exp\{-\alpha_{10}^{2}/v_{11}\}}{\sqrt{2\pi }\cdot \Gamma_{0}[\alpha_{10}/\sqrt{v_{11}}]}\;\right)\;. \end{array}$$

Applying (A41) and (A43) produces

(A44) $$\begin{array}{ll} \mathrm{Pr}\{{z^{\prime }}_{2}\leq \alpha_{2}\} & =\int_{-\infty }^{\alpha_{2}}\frac{1}{\sqrt{2\pi \cdot \mathrm{var}[{\bar{z}}_{2}]}}\cdot \exp\{-{\left(t-E[{\bar{z}}_{2}]\right)}^{2}/(2\cdot \mathrm{var}[{\bar{z}}_{2}])\}\cdot dt\\ & =\int_{-\infty }^{(\alpha_{2}-E[{\bar{z}}_{2}])/\sqrt{\mathrm{var}[{\bar{z}}_{2}]}}\frac{1}{\sqrt{2\pi }}\cdot \exp\{-t^{2}/2\}\cdot dt=\Gamma_{0}[(\alpha_{2}-E[{\bar{z}}_{2}])/\sqrt{\mathrm{var}[{\bar{z}}_{2}]}]\;. \end{array}$$

Combining (A33), (A34), and (A44) completes the proof.

## Appendix F—Proof of (62)

Making use of simple properties of probability, it can be readily verified that

(A45) $$\begin{array}{l} \mathrm{Pr}\{|<\tilde{p}{>}_{1}|\leq \Delta_{1}\;,\;|<\tilde{p}{>}_{2}|\leq \Delta_{2}\}=\mathrm{Pr}\{-\Delta_{1}\leq \;<\tilde{p}{>}_{1}\;\leq \Delta_{1}\;,\;-\Delta_{2}\leq \;<\tilde{p}{>}_{2}\;\leq \Delta_{2}\}\\ =\mathrm{Pr}\{-\Delta_{1}\leq \;<\tilde{p}{>}_{1}\;\leq \Delta_{1}\}-\mathrm{Pr}\{-\Delta_{1}\leq \;<\tilde{p}{>}_{1}\;\leq \Delta_{1}\;,\;<\tilde{p}{>}_{2}\;\geq \Delta_{2}\}-\mathrm{Pr}\{-\Delta_{1}\leq \;<\tilde{p}{>}_{1}\;\leq \Delta_{1}\;,\;<\tilde{p}{>}_{2}\;\leq -\Delta_{2}\}\;. \end{array}$$

Likewise, we have

(A46) $$\begin{array}{l} \mathrm{Pr}\{-\Delta_{1}\leq \;<\tilde{p}{>}_{1}\;\leq \Delta_{1}\;,\;<\tilde{p}{>}_{2}\;\geq \Delta_{2}\}\\ =\mathrm{Pr}\{<\tilde{p}{>}_{2}\;\geq \Delta_{2}\}-\mathrm{Pr}\{<\tilde{p}{>}_{1}\;\geq \Delta_{1}\;,\;<\tilde{p}{>}_{2}\;\geq \Delta_{2}\}-\mathrm{Pr}\{<\tilde{p}{>}_{1}\;\leq -\Delta_{1}\;,\;<\tilde{p}{>}_{2}\;\geq \Delta_{2}\}\\ =\mathrm{Pr}\{<\tilde{p}{>}_{2}\;\geq \Delta_{2}\}-\mathrm{Pr}\{-<\tilde{p}{>}_{1}\;\leq -\Delta_{1}\;,\;-<\tilde{p}{>}_{2}\;\leq -\Delta_{2}\}-\mathrm{Pr}\{<\tilde{p}{>}_{1}\;\leq -\Delta_{1}\;,\;-<\tilde{p}{>}_{2}\;\leq -\Delta_{2}\}\;, \end{array}$$

(A47) $$\begin{array}{l} \mathrm{Pr}\{-\varepsilon_{1}\leq \;<\tilde{p}{>}_{1}\;\leq \Delta_{1}\;,\;<\tilde{p}{>}_{2}\;\leq -\Delta_{2}\}\\ =\mathrm{Pr}\{<\tilde{p}{>}_{2}\;\leq -\Delta_{2}\}-\mathrm{Pr}\{<\tilde{p}{>}_{1}\;\geq \Delta_{1}\;,\;<\tilde{p}{>}_{2}\;\leq -\Delta_{2}\}-\mathrm{Pr}\{<\tilde{p}{>}_{1}\;\leq -\Delta_{1}\;,\;<\tilde{p}{>}_{2}\;\leq -\Delta_{2}\}\\ =\mathrm{Pr}\{<\tilde{p}{>}_{2}\;\leq -\Delta_{2}\}-\mathrm{Pr}\{-<\tilde{p}{>}_{1}\;\leq -\Delta_{1}\;,\;<\tilde{p}{>}_{2}\;\leq -\Delta_{2}\}-\mathrm{Pr}\{<\tilde{p}{>}_{1}\;\leq -\Delta_{1}\;,\;<\tilde{p}{>}_{2}\;\leq -\Delta_{2}\}\;. \end{array}$$

Inserting (A46) and (A47) back into (A45) yields (62).

## Appendix G—Detailed Derivation of Matrices in (92)

Performing algebraic manipulation, and using (80), we have

(A48) $$\Omega_{p}^{H}\Omega_{p}=\sum_{n=1}^{N}\sum_{k=1}^{K}|\beta_{n}{|}^{2}\cdot |{\bar{s}}_{k}{|}^{2}\cdot {\left(\frac{\partial a_{n,k}^{\prime }(p)}{\partial p^{T}}\right)}^{H}\cdot \frac{\partial a_{n,k}^{\prime }(p)}{\partial p^{T}}\;,$$

(A49) ΩRe{s¯}HΩRe{s¯}=∑n=1N|βn|2⋅AnH(p)An(p)=∑n=1N|βn|2⋅diag[||an,1′(p)||22   ||an,2′(p)||22   ⋯   ||an,K′(p)||22] ,

(A50) ΩRe{β}HΩRe{β}=diag[∑k=1K|s¯k|2⋅||a1,k′(p)||22∑k=1K|s¯k|2⋅||a2,k′(p)||22⋯∑k=1K|s¯k|2⋅||aN,k′(p)||22] ,

(A51) ΩpHΩRe{s¯}=[∑n=1N|βn|2⋅(∂an,1′(p)∂pT)Han,1′(p)∑n=1N|βn|2⋅(∂an,2′(p)∂pT)Han,2′(p)⋯∑n=1N|βn|2⋅(∂an,K′(p)∂pT)Han,K′(p)]⋅diag[s¯∗] ,

(A52) ΩpHΩRe{β}=[∑k=1K|s¯k|2⋅(∂a1,k′(p)∂pT)Ha1,k′(p)∑k=1K|s¯k|2⋅(∂a2,k′(p)∂pT)Ha2,k′(p)⋯∑k=1K|s¯k|2⋅(∂aN,k′(p)∂pT)HaN,k′(p)]⋅diag[β∗] ,

(A53) ΩRe{β}HΩRe{s¯}=diag[β]⋅[A1H(p)A1(p)s¯A2H(p)A2(p)s¯⋯ANH(p)AN(p)s¯]H .

Firstly, inserting (A48), (A49), and (A51) into the first equality in (92) yields

(A54) V1,1=ΩpHΩp−ΩpHΩRe{s¯}(ΩRe{s¯}HΩRe{s¯})−1ΩRe{s¯}HΩp=∑n=1N∑k=1K|βn|2⋅|s¯k|2⋅(∂an,k′(p)∂pT)H⋅∂an,k′(p)∂pT−∑k=1K(|s¯k|2∑n=1N|βn|2⋅||an,k′(p)||22)(∑n1=1N∑n2=1N|βn1βn2|2⋅(∂an1,k′(p)∂pT)Han1,k′(p)an2,k′H(p)⋅∂an2,k′(p)∂pT) .

Secondly, substituting (A49), (A51), (A52), and (A53) into the second equality in (92) gives

(A55) V1,2=[1  j]⊗(ΩpHΩRe{β}−ΩpHΩRe{s¯}(ΩRe{s¯}HΩRe{s¯})−1ΩRe{s¯}HΩRe{β})=[1  j]⊗[V1,2(1)  V1,2(2)  ⋯  V1,2(N)] ,

where

(A56) $$V_{1,2}^{\left(n\right)}=\sum_{k=1}^{K}\left|{\bar{s}}_{k}{|}^{2}\cdot {\left(\frac{\partial a_{n,k}^{\prime }(p)}{\partial p^{T}}\right)}^{H}a_{n,k}^{\prime }(p\right)-\sum_{k=1}^{K}\frac{\beta_{n}^{*}\cdot |{\bar{s}}_{k}{|}^{2}\cdot ||a_{n,k}^{\prime }(p)|{|}_{2}^{2}}{\sum_{n_{1}=1}^{N}|\beta_{n_{1}}{|}^{2}\cdot ||a_{n_{1},k}^{\prime }(p)|{|}_{2}^{2}}\cdot \sum_{n_{2}=1}^{N}\left|\beta_{n_{2}}{|}^{2}\cdot {\left(\frac{\partial a_{n_{2},k}^{\prime }(p)}{\partial p^{T}}\right)}^{H}a_{n_{2},k}^{\prime }(p\right)\;(1\leq n\leq N)\;.$$

Finally, putting (A49), (A50), and (A53) into the third equality in (92) leads to

(A57) V1,3=[1j−j1]⊗(ΩRe{β}HΩRe{β}−ΩRe{β}HΩRe{s¯}(ΩRe{s¯}HΩRe{s¯})−1ΩRe{s¯}HΩRe{β})=[1j−j1]⊗(diag[∑k=1K|s¯k|2⋅||a1,k′(p)||22∑k=1K|s¯k|2⋅||a2,k′(p)||22⋯∑k=1K|s¯k|2⋅||aN,k′(p)||22]−diag[β]⋅[A1H(p)A1(p)s¯A2H(p)A2(p)s¯⋯ANH(p)AN(p)s¯]H×diag[(∑n=1N|βn|2⋅||an,1′(p)||22)−1(∑n=1N|βn|2⋅||an,2′(p)||22)−1⋯(∑n=1N|βn|2⋅||an,K′(p)||22)−1]×[A1H(p)A1(p)s¯A2H(p)A2(p)s¯⋯ANH(p)AN(p)s¯]⋅diag[β∗]) .

## Appendix H—Proof of (96)

We start by introducing a real array model error vector φ˜r=[ReT{φ˜}  ImT{φ˜}]T with probability density function given by

(A58) $$f_{{\tilde{\varphi }}_{r}}(z)={\left(2\pi \right)}^{-MN}\cdot |\det[\Phi ]{|}^{-1/2}\cdot \exp\{-z^{T}\Phi^{-1}z/2\}\;.$$

When the deterministic and stochastic parameters coexist, the Fisher information matrix (FIM) for vector $\eta_{b}$ is given by [68,69],

(A59) $$<\mathrm{FISH}(\eta_{b}){>}_{ij}=E\left[\frac{\partial^{2}f_{\mathrm{ml}}(\eta_{b}|\bar{x})}{\partial <\eta_{b}{>}_{i}\partial <\eta_{b}{>}_{j}}\right]+E\left[\frac{1}{2}\cdot \frac{\partial^{2}{\tilde{\varphi }}^{T}\Phi^{-1}\tilde{\varphi }}{\partial <\eta_{b}{>}_{i}\partial <\eta_{b}{>}_{j}}\right]\;,$$

where $f_{\mathrm{ml}}(\eta_{b}|\bar{x})$ is the ML function of the compound data vector $\bar{x}$. Combining (A59) and the results in [66,67], the FIM for vector $\mu_{b}$ can be expressed as

(A60) <FISH(μb)>ij=2σε2⋅<Re{ΩμbHΩμb}>ij+<Φ−1>ij⋅δ(i,j) ,

where δ(i,j) is an indicator function such that δ(i,j)=1 if both i and j correspond to the element in ${\tilde{\varphi }}_{r}$, and δ(i,j)=0 otherwise. It follows from (A60) that

(A61) CRB(μb)=(FISH(μb))−1=(2σε2⋅Re{ΩμbHΩμb}+[OOOΦ−1])−1 ,

which completes the proof.

## Appendix I—Detailed Derivation of Matrices in (101)

Note that matrices $\Omega_{p}^{H}\Omega_{p}$, ΩRe{β}HΩRe{β}, ΩRe{s¯}HΩRe{s¯}, ΩpHΩRe{β}, ΩpHΩRe{s¯}, and ΩRe{β}HΩRe{s¯} are given in (A48) to (A53). Therefore, to calculate the matrices in (101), we only need to derive the expressions for matrices ΩRe{φ˜}HΩRe{φ˜}, ΩpHΩRe{φ˜}, ΩRe{β}HΩRe{φ˜}, and ΩRe{s¯}HΩRe{φ˜}. It follows from (99) that

(A62) ΩRe{φ˜}HΩRe{φ˜}=blkdiag[|β1|2⋅||s¯||22⋅IM|β2|2⋅||s¯||22⋅IM⋯|βN|2⋅||s¯||22⋅IM] ,

(A63) ΩpHΩRe{φ˜}=[∑k=1K|β1|2⋅|s¯k|2⋅exp{−jωkτ1(p)}⋅(∂a′1,k(p)∂pT)H∑k=1K|β2|2⋅|s¯k|2⋅exp{−jωkτ2(p)}⋅(∂a′2,k(p)∂pT)H⋯⋯∑k=1K|βN|2⋅|s¯k|2⋅exp{−jωkτN(p)}⋅(∂a′N,k(p)∂pT)H] ,

(A64) ΩRe{β}HΩRe{φ˜}=blkdiag[||s¯||22⋅β1a1H(p)||s¯||22⋅β2a2H(p)⋯||s¯||22⋅βNaNH(p)] ,

(A65) ΩRe{s¯}HΩRe{φ˜}=[|β1|2⋅A1H(p)(s¯′1⊗IM)|β2|2⋅A2H(p)(s¯′2⊗IM)⋯|βN|2⋅ANH(p)(s¯′N⊗IM)]=[|β1|2⋅s¯a1H(p)|β2|2⋅s¯a2H(p)⋯|βN|2⋅s¯aNH(p)] .
